# Hydrogen-bonded host–guest systems are stable in ionic liquids

**DOI:** 10.1038/s41598-020-71803-3

**Published:** 2020-09-22

**Authors:** Teresa Naranjo, Rubén Álvarez-Asencio, Patricia Pedraz, Belén Nieto-Ortega, Sara Moreno-Da Silva, Enrique Burzurí, Mark W. Rutland, Emilio M. Pérez

**Affiliations:** 1 IMDEA Nanociencia, Faraday 9, Campus UAM, 28049 Madrid, Spain; 2 RISE Research Institutes of Sweden, Bioscience and Materials, 114 28 Stockholm, Sweden; 3 Division of Surface and Corrosion Science, KTH Royal Institute of Technology, Drottning Kristinas 51, 100 44 Stockholm, Sweden

**Keywords:** Chemistry, Supramolecular chemistry

## Abstract

We show that H-bonded host–guest systems associate in ionic liquids (ILs), pure salts with melting point below room temperature, in which dipole–dipole electrostatic interactions should be negligible in comparison with dipole-charge interactions. Binding constants (*K*_a_) obtained from titrations of four H-bonded host–guest systems in two organic solvents and two ionic liquids yield smaller yet comparable *K*_a_ values in ionic liquids than in organic solvents. We also detect the association event using force spectroscopy, which confirms that the binding is not solely due to (de)solvation processes. Our results indicate that classic H-bonded host–guest supramolecular chemistry takes place in ILs. This implies that strong H-bonds are only moderately affected by surroundings composed entirely of charges, which can be interpreted as an indication that the balance of Coulombic to covalent forces in strong H-bonds is not tipped towards the former.

## Introduction

Current approximations to covalent bonding have proven remarkably accurate based on their good match with state-of-the-art molecule imaging techniques^1–3^. Meanwhile, supramolecular chemistry has advanced greatly in accurately determining the free energy of binding between molecular species (i.e. the association constant)^4,5^. From these data, supramolecular chemists have established “reaction” patterns, much like in the early days of covalent chemistry. This noncovalent reactivity has already been used successfully to make some of the most outstanding synthetic architectures^6,7^, and even mimic some of life’s most fundamental features, like recognition and self-replication^8^. However, despite the enormous advances in supramolecular chemistry as a synthetic tool^9^, chemists still argue about the nature, or even the existence^10^, of particular intermolecular interactions. Specifically, the nature of H-bond is the subject of a heated debate. H-bonds have traditionally been understood as a largely electrostatic interaction^11^, but the question of a relevant covalent contribution to H-bonding keeps resurfacing^12–14^.

More recently, Weinhold and Klein presented the ultimate argument in favor of covalent-dominated H-bonds: anti-electrostatic H-bonds between two molecular species with identical charge^15^. Their conclusions were immediately refuted by Frenking and Caramori^16^, and later re-claimed by Weinhold and Klein^17^. From the experimental point of view, Flood and co-workers have provided irrefutable evidence for the existence of stabilizing anion-anion H-bonds in solution, within macrocyclic cavities^18^. Adding to this debate, Moriarty and co-workers have used non-contact atomic force microscopy to image H-bonded networks with sub-molecular resolution, and concluded that the short-range energy force and potential energy landscapes of intermolecular H-bonds were “surprisingly similar” to intramolecular C–C bonds^19^.

Motivated by this debate, in 2011 the IUPAC moved away from a definition of H-bond that included the very explicit “best considered as an electrostatic interaction” to the much more neutral “is an attractive interaction between a hydrogen atom from a molecule or a molecular fragment X–H in which X is more electronegative than H, and an atom or a group of atoms in the same or a different molecule, in which there is evidence of bond formation”^20^.

The trouble in putting this ongoing debate to rest is most likely due to the difficulty in measuring the parameters that define a noncovalent bond -strength and directionality- in solution, where the interaction of the solvent molecules with the solute(s), and with themselves, are of identical nature and similar magnitude^21^, and are often the driving force behind assembly, particularly in water^22^. Cleverly designed structural variation schemes have been used to overcome these limitations. For example, Hunter has used “mutant” and “double-mutant” cycles, in which the structure of H-bonding donors and acceptors is changed in order to dissect the different contributions to the overall thermodynamic stability of the H-bond^23^.

## Results and discussion

In the present manuscript we compare the stability of several host–guest pairs in organic solvents and ionic liquids (ILs). ILs are pure salts that are liquid under ambient conditions^24^. As liquids composed entirely of ions, they are the ideal playground to test the contribution of Coulombic interactions to H-bonding. In principle, in ILs dipole–dipole electrostatic interactions (proportional to r^−6^ for randomly oriented dipoles and r^−3^ once oriented) should be negligible in comparison with dipole-point charge interactions (proportional to r^−4^ for random orientation and r^−2^ once oriented). This purely electrostatic picture has been proven true for weak H-bonds in water, which exists in non self-associated form in ionic liquids, H-bonded to the anions, forming anion–H–O–H–anion associates^25^.

**Figure 1:**
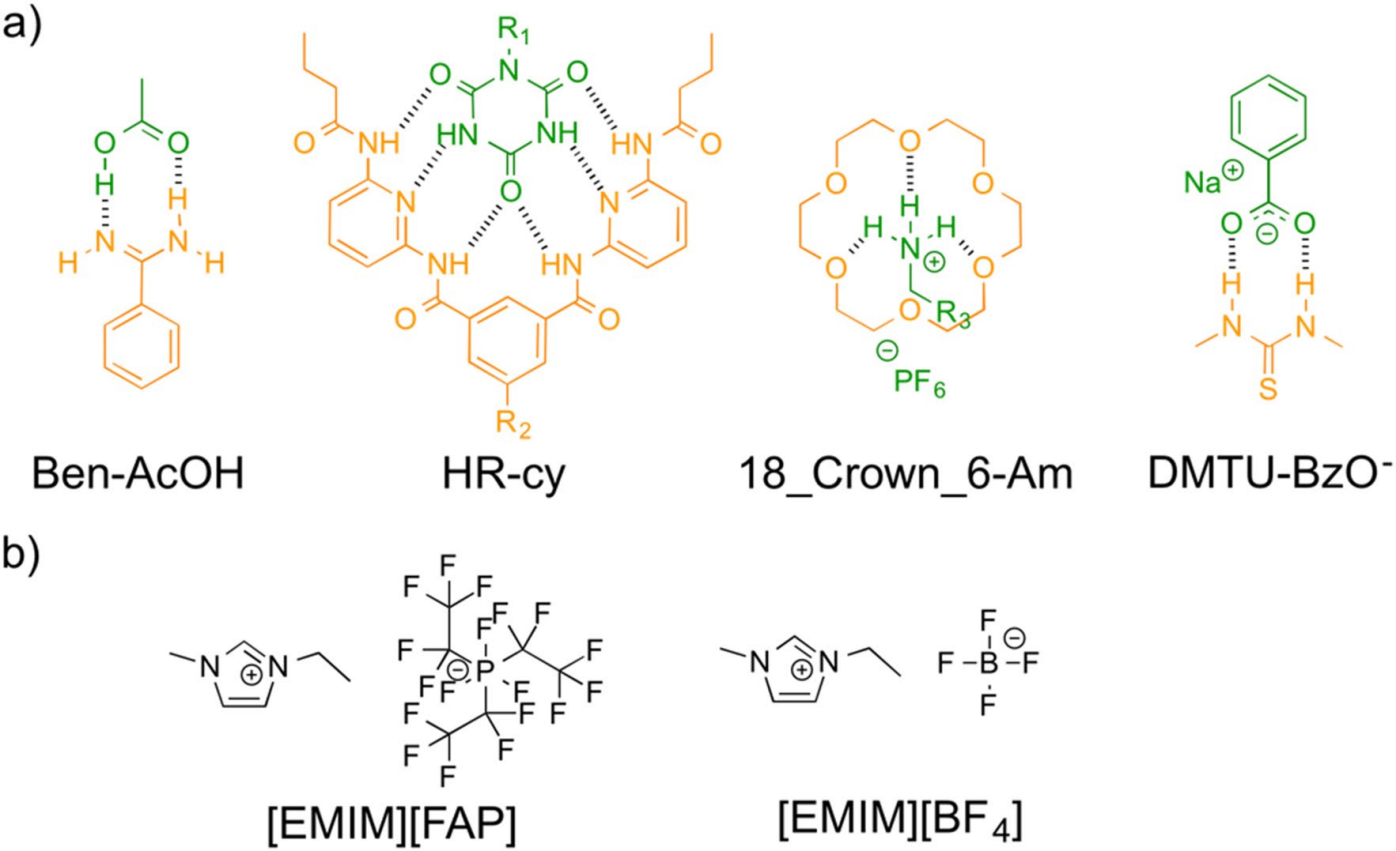
(**a**) Chemical structure of the host–guest systems under study and (**b**) chemical structure of the two ionic liquids used: 1-ethyl-3-methylimidazolium tris(pentafluoroethyl) trifluoro phosphate ([EMIM][FAP]) and 1-ethyl-3-methylimidazolium tetrafluoroborate ([EMIM][BF_4_]).

The ILs selected for this study are 1-ethyl-3-methylimidazolium tris(pentafluoroethyl) trifluoro phosphate ([EMIM][FAP]), 1-ethyl-3-methylimidazolium tetrafluoroborate ([EMIM][BF_4_]) which are represented in Fig. 1b. Organic solvents (chloroform and acetonitrile) were selected to ensure complete solubility of all species in the host–guest binding equilibrium. The van der Waals radius of the anions of these ILs is sufficiently small to guarantee that purely electrostatic dipole–dipole interactions should be negligible compared to dipole–charge interactions (see the Supporting Information). Therefore, from a purely electrostatic view of H-bonding, this fact should strongly affect the *K*_a_ values of the host–guest systems under study. In accordance with this, the polarities of the ILs, according to the ${\text{ E}}_{{\text{T}}}^{{\text{N}}}$ scale, are much larger than the ones corresponding to the organic solvents^26–28^.

**Figure 2:**
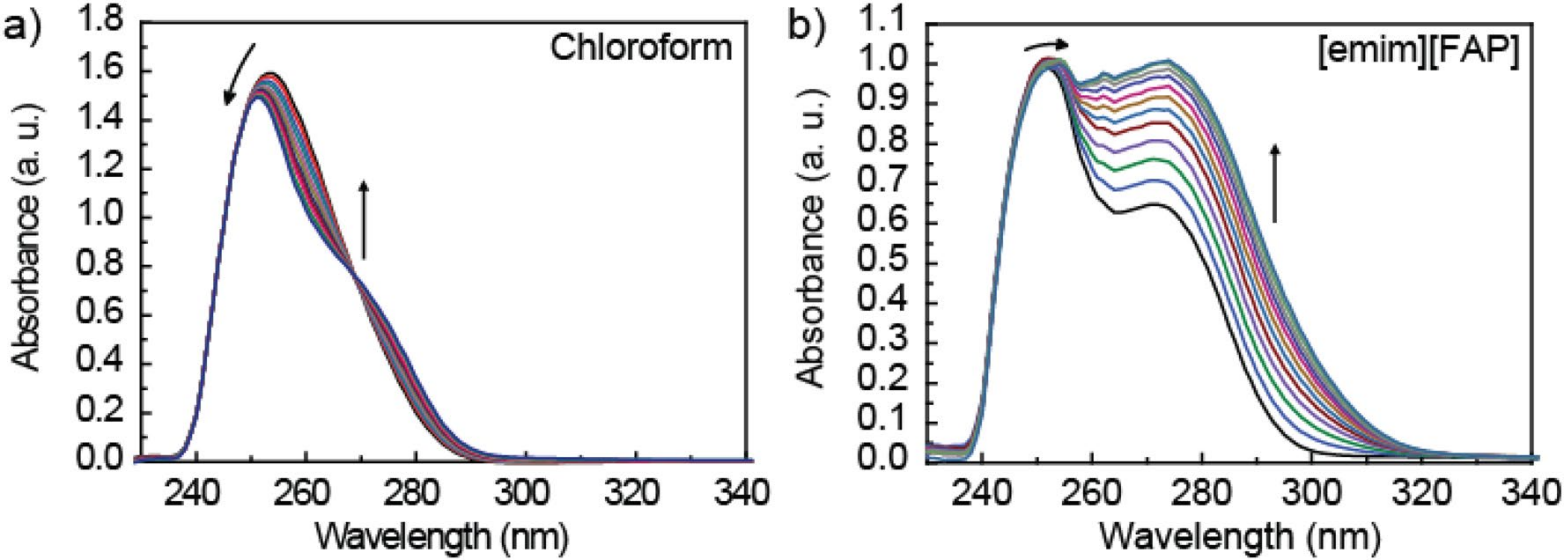
Spectroscopic changes observed in the UV–vis titration of the Ben-AcOH couple in (**a**) chloroform and (**b**) [EMIM][FAP]. UV/vis spectra of Benzamidine (6.5 × 10^−4^ M) on addition of AcOH (0 → 3 equiv.). Titrations were carried out at room temperature and at constant concentration of the host.

The hydrogen bonded host–guest systems have been thoroughly studied previously, are synthetically accessible, and span a large range of *K*_a_. In particular, we chose the Hamilton receptor-cyanuric acid derivative pair (HR-cy in Fig. 1, neutral–neutral), benzamidine-acetic acid (Ben-AcOH in Fig. 1, neutral–neutral), N,N′-dimethyl thiourea-benzoate (DMTU-BzO^−^ in Fig. 1, neutral-anion) and the 1,4,7,10,13,16-hexaoxacyclooctadecane-ammonium derivative (18_Crown6-Am in Fig. 1, neutral-cation). We first investigated the *K*_a_ of the host–guest systems in solution, using UV–vis titrations. Very briefly, we analyze the UV–vis spectrum of a solution of the host with increasing concentration of the guest. To eliminate dilution effects, we work at constant concentration of the host. The spectral data were fitted using Reactlab Equilibria global fitting software (see the Supporting Information). All data fitted satisfactorily to 1:1 binding model. An example of the UV–vis titration results for the first couple, Ben-AcOH is shown in Fig. 2. The evolution of the spectra in chloroform and [EMIM][FAP] during the titration follow similar trends, and are clearly indicative of association. In the titrations performed in chloroform, we observe a depletion of the Ben absorption maximum centered at 254 nm, which is accompanied by a blue-shift to 250 nm. These changes are concomitant with the appearance of a new absorption band at around 272 nm, with a clear isosbestic point at 269 nm. In the case of titrations performed using [EMIM][FAP] as solvent, we observe that the absorption band at 271 nm, indicative of Ben H-bonding, is already present in the absence of AcOH guest, clearly indicating that the Ben is initially H-bonded to itself forming a dimer, and/or to the IL. Upon addition of AcOH, the intensity of this band increases significantly and is red-shifted to 274 nm, while the UV maximum at 251 nm is slightly red-shifted to 254 nm. These changes occur without the formation of a clear isosbestic point, in accordance with a picture where Ben coexists as free species and H-bonded both to the IL and to the AcOH guest. However, it is clear from the evolution of the spectra during the titration that the AcOH guest is the strongest binder, and displaces the equilibrium towards Ben-AcOH host–guest complexes. This is also reflected in the good fit to a 1:1 binding mode. The calculated binding constants are in the range previously reported for amidine-carboxylic acid derivatives^29,30^. The somewhat unexpected fact that we observe a larger binding constant in CH_3_CN compared to CHCl_3_ is most likely due to a larger degree of proton transfer in the more polar solvent^30^. Finally, note that a significant concentration of the AcOH homodimer is expected to form in pure AcOH (vide IR section below)^31–33^, but should be a small fraction in dilute solution (log *K*_d _≈ 2 in CHCl_3_)^34^. We have nevertheless also carried out the fittings including the dimerization equilibrium (see the Supporting Information). The fittings are not improved, and the main conclusion, that binding is approximately as strong in organic solvents as in ILs, holds true.

To facilitate the analysis of the UV–vis spectra of the second and third pairs, that is, HR-cy and 18_Crown6-Am, cy and Am, they were tagged with a UV–vis dye, [Ru(bpy)_3_][PF6]_2_. In all cases, the changes in absorption spectra during titrations in organic solvents were qualitatively similar to those observed in ILs and fitted well to 1:1 binding equilibria (see the Supporting Information)^35^. The values of log *K*_a_ obtained from these titrations are summarized in Table 1. In all cases, *K*_a_ in ILs were generally slightly lower but of comparable order to those found in organic solvents.

**Table 1:** Summary of *K*_a_ values obtained from fittings to a 1:1 binding model.

	Ben-AcOH^a^	HR-cy	Crown-Am	DMTU-BzO^−^
log *K*_a_ (CH_3_CN)	4.4 ± 0.1	6.5 ± 0.4	3.8 ± 0.3	4.4 ± 0.3
log *K*_a_ (CHCl_3_)	3.4 ± 0.2	–	–	4.6 ± 0.1
log *K*_a_ ([EMIM][FAP])	3.3 ± 0.2	5.8 ± 0.2	3.5 ± 0.4	4.6 ± 0.3
log *K*_a_ ([EMIM][BF_4_])	2.9 ± 0.3	5.9 ± 0.3	2.9 ± 0.2	4.2 ± 0.3

Values not reported could not be determined due to solubility issues.

^a^The Ben-AcOH system has also been fitted considering the dimerization of AcOH, see the Supporting Information.

**Figure 3:**
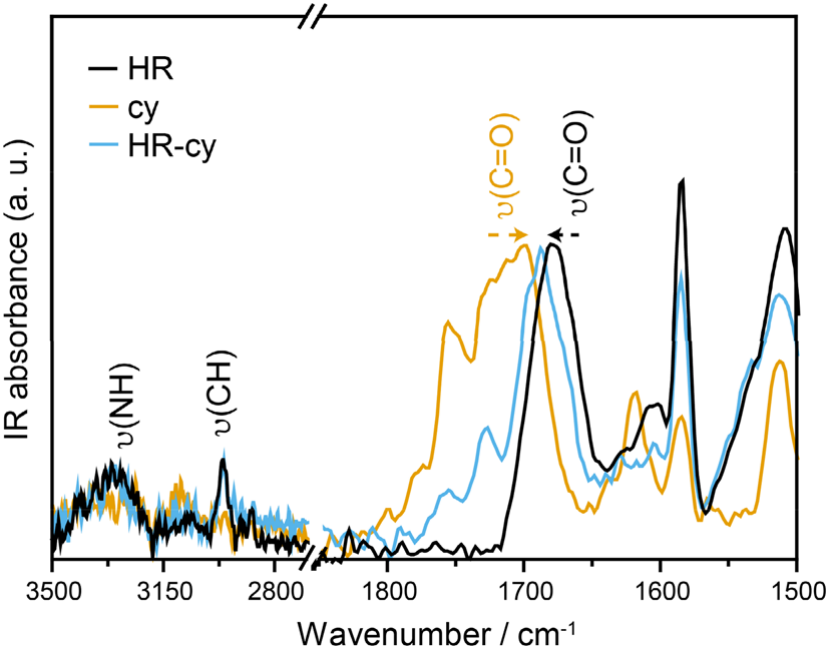
ATR-IR spectra in [EMIM][FAP] of HR (black), cy (orange) and the 1:1 molar mixture of both compounds, HR-cy (blue) at 7.0 10^−3^ M. The spectrum of the solvent ([EMIM][FAP]) has been subtracted.

Spectroscopic characterization of the host–guest systems in ILs indicates their formation through H-bonds, rather than unspecific solvophobic interactions. Figure 3 represents the solvent-corrected attenuated total reflection Fourier transform IR (ATR-FTIR) spectra of HR, cy and the 1:1 molar mixture of both compounds, HR-cy, at 7.0 × 10^−3^ M using [EMIM][FAP] as solvent. HR presents an ATR-FTIR spectra with the most intense bands in the region of 1,700–1,400 cm^−1^. The most interesting band is the ν(C=O) carbonyl stretching normal mode, which appears at 1,679 cm^−1^ in the HR, indicating a weak C=O double bond character. In the case of the cyanuric acid, we observe a broad band centered at 1,714 cm^−1^ with a lower intensity band at higher wavenumber at 1,766 cm^−1^. These bands can be assigned to the different C=O moieties in the molecular structure. Interestingly, in the IR spectrum of the 1:1 HR-cy mixture, in which the solvent has been subtracted, we observe a broad band with several shoulders. Thus, the IR spectrum of the mixture is not the arithmetic sum of the parent spectra. We can associate the shoulder at 1,696 cm^−1^ with a carbonyl from the cy that is downshifted upon formation of the HR-cy couple. On the other hand, the more intense peak can be associated with a carbonyl from the HR whose double bond character has been reinforced after the association event. The less intense band at 1,729 cm^−1^ can be associated to a carbonyl of the cy that does not interact with the HR (see chemical structure of the HR-cy complex). All these changes are in very good agreement with the formation of the expected HR-cy dimer via H-bonds. Unfortunately, the low intensity of the signals in the 3,500–3,000 cm^−1^ prevents analysis of this region. A similar analysis for the Ben-AcOH couple can be found in the Supporting Information. All the ATR-FTIR data point clearly to H-bonding in the host–guest systems.

**Figure 4:**
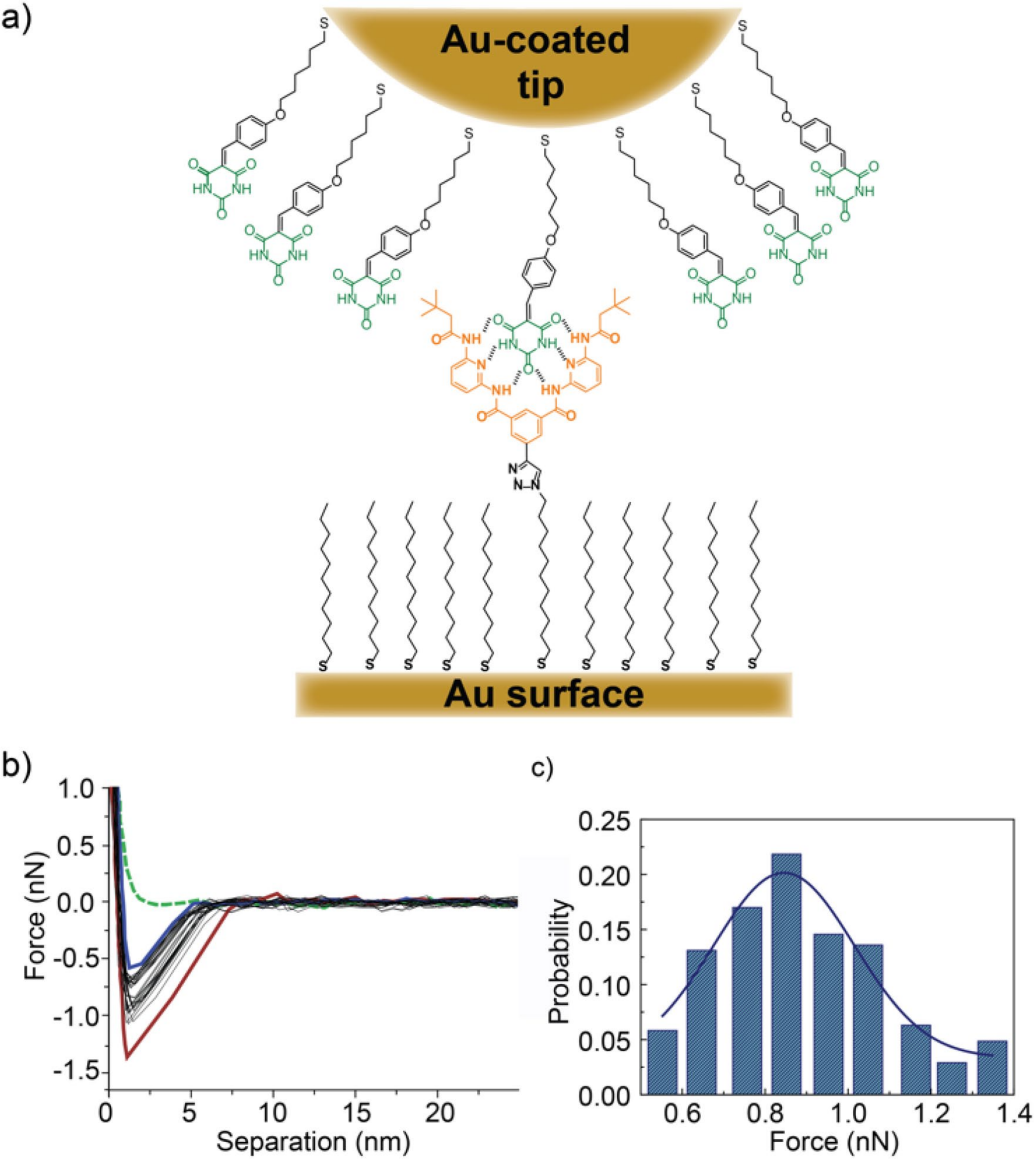
(**a**) Schematic representation of AFM-SMFS of HR-cy derivative. Cy guest (in green) is immobilized in a gold-coated AFM tip, while the gold-coated surface is functionalized with the HR (in orange). (**b**) Representative force/distance curves obtained in [EMIM][FAP], which characterizes the interaction that occurs between HR-cy couple. The force profiles represent the interaction forces upon approach (green dash trace) and retraction (twenty aligned black traces, showing a purple trace which represents the lowest breaking force and a red trace which corresponds to the highest breaking force) as a function of the separation distance between the surface and the tip. Approaching the sample surface with the AFM probe, no interaction forces were detected, when the retraction take place we can observe a binding event, getting a dissociation force of 845 ± 17 pN. (**c**) Force histogram fitting to a Gaussian distribution, obtained for 206 curves in ten different regions.

We note that, to a first and stoichiometrically wrong approximation, association constants are a measurement of the relative combined stabilities of the host/guest + IL/IL and the host/IL guest/IL systems. That is, strong IL/IL interactions can lead to host–guest association even if the interaction of both host and guest with the IL is larger than with each other. To gather direct information of the relative strength of the host–guest H-bonding event in the different media, we turned to force spectroscopy (FS)^36–38^. In particular, we focused on the HR-cy pair, as benchmark data on SMFS in o-dichlorobenzene (o-DCB) are available from Bassani et al.^39^. We decorated a gold-coated AFM tip with the cy guest, and a gold surface with the HR host, using thiol-Au chemistry (Fig. 4a). Force-distance measurements were recorded at velocities of 250 nm/s. After reaching the maximal force, the two surfaces remained in contact for a time interval of 5 s, after which the direction of the piezo movement was reversed and the AFM probe was retracted from the surface. Force-distance curves were collected from several different positions on the substrate and around 60 force curves were measured at each position.

We first carried out measurements in o-DCB. From the statistical analysis of 120 measurements we obtain a binding strength of 434 pN for the HR-cy host–guest system, with upper and lower bounds at 164 and 839 pN, respectively (see the Supporting Information). This value is in good agreement with that found by Bassani et al.^39^ The retraction curves on the HR-cy system in [EMIM][FAP] also clearly showed binding events (Fig. 4b), in agreement with the bulk data. The strength of the binding event from the raw data was calculated to be 845 ± 17 pN for 206 binding events, with upper and lower bounds at 1,400 and 500 pN, respectively, significantly larger than that found in o-DCB (Fig. 4c). These results are explained in light of the control experiments. For the thiol-functionalized tip and HR-functionalized gold surface, we obtained a peak in the retraction curve that amounts to 661 ± 22 pN, for 250 measurements (see the Supporting Information). These results show that there is a nonspecific interaction between the tip and the first few layers of IL, in close contact with the gold surface. Subtracting this interaction from the HR-cy results, we obtain a force for the HR-cy binding event of ca. 184 ± 28 pN, which is smaller, but within the same order of magnitude of that obtained in the nonpolar o-DCB. The FS data are therefore in perfect agreement with the bulk titration experiments, and confirm beyond reasonable doubt that the H-bonded HR-cy host–guest species exists in IL, and its stability is similar to that in nonpolar organic solvents^40^.

In summary, our data show that H-bonded host–guest association occurs in ILs. The complexes show similar stabilities in ILs as in organic solvents. Moreover, direct measurement of the host–guest interaction indicates that strong H-bonds are not (completely) shielded in ILs, where electrostatic interactions are expected to be considerably less significant. This is opposed to the findings for weaker H-bonds, such as those in water, which are substituted by O–H–anion interactions in ILs^25^. We interpret the results of our experiments as pointing towards a significant covalent contribution to strong H-bonds, which should not be affected by the IL charges, and away from a purely electrostatic view of H-bonding^41^. However, we note that this work is just another piece towards completion of a very complicated puzzle. Further, and hopefully combined, experimental and theoretical developments^42–48^ will be required before the nature of noncovalent interactions can be fully elucidated.

## Supplementary information

Supplementary Information.
