# Supplementary material for: Hydrogen-bonded host–guest systems are stable in ionic liquids
Source: Sci Rep. 2020 Sep 22;10:15414. doi: 10.1038/s41598-020-71803-3 (PMC7508985; doi:10.1038/s41598-020-71803-3)
Supplement: Supplementary file 1 — Supplementary Information. [file 41598_2020_71803_MOESM1_ESM.pdf]

# Hydrogen-bonded Host-Guest Systems Are Stable in Ionic Liquids

Teresa Naranjo,[a] Rubén Álvarez-Asencio,[b] Patricia Pedraz,[a] Belén Nieto-Ortega,[a] Sara Moreno-Da Silva,[a] Enrique Burzurí,[a] Mark W. Rutland[b,c,*] and Emilio M. Pérez[a,*]

[a] *IMDEA Nanociencia, Faraday 9, Campus UAM, 28049 Madrid, Spain; E-mail: emilio.perez@imdea.org.*

[b] *RISE Research Institutes of Sweden, Bioscience and Materials, Stockholm, SE-114 28, Sweden.*

[c] *KTH Royal Institute of Technology, Division of Surface and Corrosion Science, Drottning Kristinas 51, SE-100 44 Stockholm, Sweden. E-mail: mark@kth.se.*

**Supplementary Information**

**Contents:**

**1.** Experimental Approach
**2.** Synthesis of the HR-cy couple
**3.** Synthesis of Am derivative
**4.** Synthesis HR-barb couple for AFM studies
**5.** Description of UV/Vis titration experiments
**6.** Description of ATR-IR experiments with RTIL
**7.** AFM Tip and surface functionalization
**8.** SAM characterization
**9.** AFM measurements

## General

All solvents were dried according to standard procedures. Reagents were used as purchased. All air-sensitive reactions were carried out under argon atmosphere. Flash chromatography was performed using silica gel (Merck, Kieselgel 60, 230-240 mesh, or Scharlau 60, 230-240 mesh). Analytical thin layer chromatographies (TLC) were performed using aluminium-coated Merck Kieselgel 60 F254 plates. NMR spectra were recorded on a BrukerAvance 400 ($^1$H: 400 MHz; $^{13}$C: 100 MHz), spectrometer at 298 K, unless otherwise stated, using partially deuterated solvents as internal standards. Coupling constants ($J$) are denoted in Hz and chemical shifts ($\delta$) in ppm. Multiplicities are denoted as follows: s = singlet, d = doublet, t = triplet, m = multiplet, br = broad. Fast Atom Bombardment (FAB) and Matrix-assisted Laser desorption ionization (coupled to

a Time-Of-Flight analyzer) experiments (MALDI-TOF) were recorded on a VS AutoSpec spectrometer and a Bruker ULTRAFLEX III spectrometer, respectively.

## 1. Experimental approach

First, let´s briefly overview the purely electrostatic part of supramolecular interactions. In Table S1 are summarized the energy values of the types of electrostatic interactions that will coexist in our experimental design. The orientation of the dipoles and the ions can vary due to thermal effects, a weighted angle average must be calculated in order to estimate the average net interaction energy; this consideration has been taken into account in formulas of ion-diopole and dipole-dipole interaction.[1]

Table S1 summarizes the electrostatic interactions, and associated energy V (r), between different species that might coexist in our experimental design: ion-ion, ion-dipole and dipole-dipole couples. The relative orientation (angle θ) between dipoles and ions or other dipoles fluctuates due to thermal effects. The average interaction energy $\langle V(r)\rangle$ is therefore calculated as a weighted angle average by integrating over all possible θ values.

| Interaction | Energy | Scheme |
|---|---|---|
| **Ion pairing** | $V(r) = -\frac{q_1 q_2}{4\pi\varepsilon_0 r}$ | 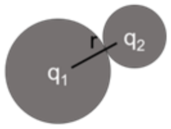  |
| **Ion – Dipole** | $\langle V(r)\rangle = -\frac{(q_2\mu_1)^2}{3k_B T(4\pi\varepsilon_0)^2}\frac{1}{r^4}$ | 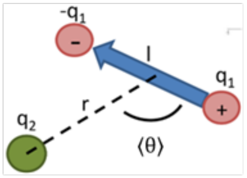  |
| **Dipole - Dipole** | $\langle V(r)\rangle = -\frac{1}{3}\frac{1}{k_B T}\left(\frac{\mu_1\mu_2}{4\pi\varepsilon_0}\right)^2\frac{1}{r^6}$ | 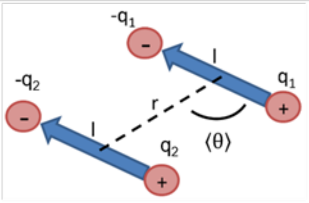  |

**Table S1**. Electrostatic interaction energy between ion-ion, ion-dipole and dipole-dipole couples where *r* is the distance between species, *q* is the charge of the ion, $\varepsilon_0$ is the dielectric constant and *μ* is the dipole moment. The dipole is represented by a vector directed form the positive to the negative charge.

We have calculated the dipole ($\mu_1$)-dipole ($\mu_2$) energy interaction of a simple hydrogen bonded system described in Figure S1a and compared it with the dipole ($\mu_1$)-ion ($q$) interaction where $q$ = 1e$^-$. The result is shown in Figure S1b. For a given $r$ value, the dipole-ion interaction is preferred over the dipole-dipole interaction for any realistic value of $r$. This is the case for example for the average dipole-dipole bond distance ($r$ = 2.5 Å) of dipoles $\mu_1$ and $\mu_2$ in Figure S1, marked with a green line in Figure S1b.

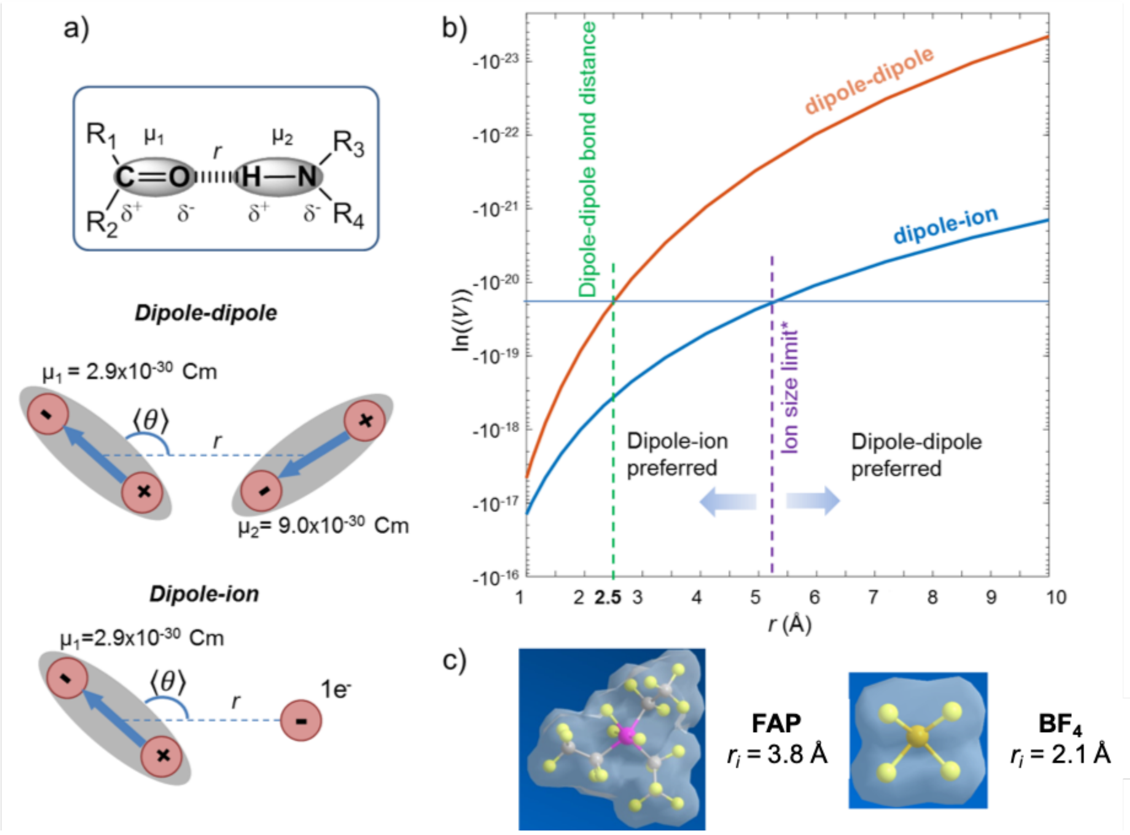


**Figure S1**. a) Hydrogen bonded system selected for the theoretical calculations, showing $q$, $\mu_1$ and $\mu_2$ values b) Theoretical electrostatic energy $\langle V(r)\rangle$ versus distance $r$ obtained for dipole-dipole and dipole-ion interaction. c) FAP and $BF_4$ anions, showing their ionic radius $r_i$.[2-4]

If the size of the ion is now taken into account, there is a minimum dipole-ion distance below which ion and dipole cannot get closer (in addition to the van der Waals radius). This distance can be larger than the dipole-dipole H-bond distance. Assuming for simplicity a localized charge in the ion, there is therefore an ion size limit over which the dipole-dipole interactions become dominant (purple line in Figure S1b). The size of the anions used in this work (FAP and $BF_4$) lies below the calculated ion size limit (see Figure S1c). It is therefore expected that the amine dipole will prefer to bond with the anions instead of with the other dipoles. According to this, from a purely electrostatic

point of view, the association constants of the host-guest systems under study would be strongly affected by the presence of ILs.

## 2. Synthesis of the HR-cy couple

### 2.a. Cy synthesis

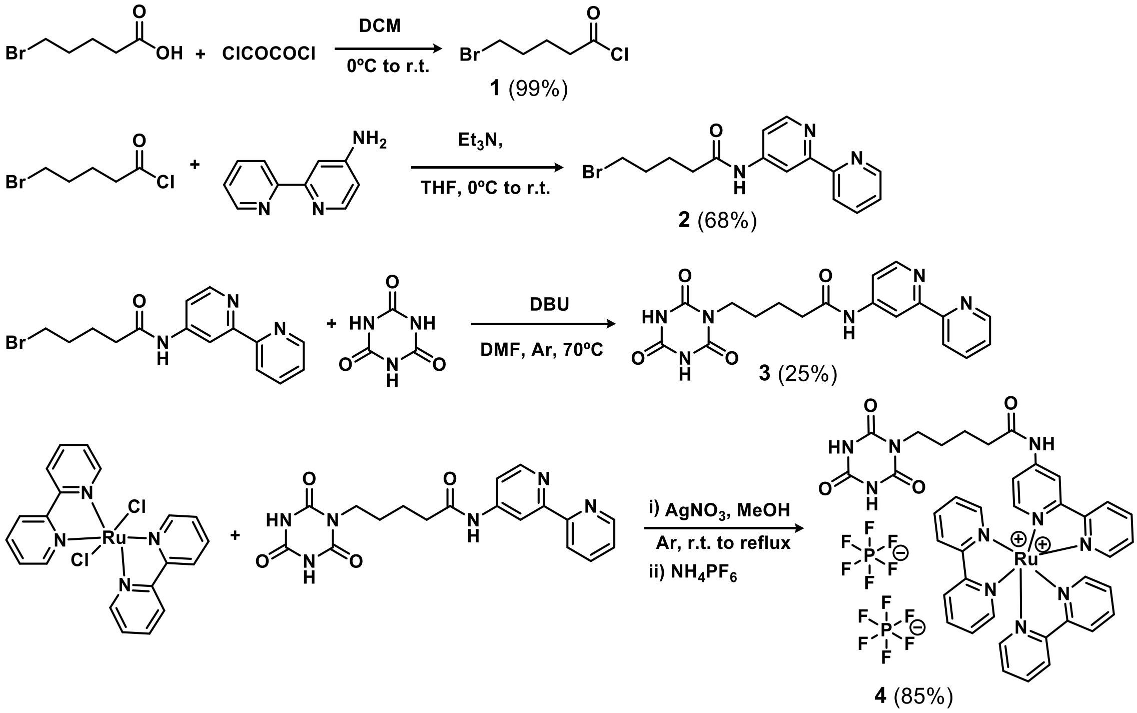


### 5-bromopentanoyl chloride

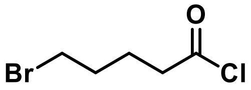

**Compound 1**

5-Bromovaleric acid (200 mg 1.1 mmol) was suspended in anhydrous DCM (4.4 mL), 2 drops of DMF were added and oxalyl chloride (0.12 mL, 1.4 mmol) was added dropwise over a period of 15 min. The reaction was stirred for two hours, then the solvent was removed under reduced pressure and the crude material was directly used in the next step reaction without further purification.

***N*-[(2,2'-bipyridin)-4-yl]-5-bromopentanamide**

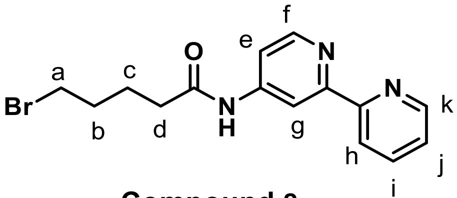


**Compound 2**

A solution of compound **1** (219 mg, 1.1 mmol) in dry THF (4.5 mL) was added dropwise to a solution of [2,2'-bipyridin]-4-amine (179 mg, 1.05 mmol) and TEA (0.17 mL, 1.26 mmol) in dry THF (4.5 mL) at 0ºC under Ar atmosphere. The solution was stirred at r.t. overnight. Then a white residue was filtered off and the solvent removed under reduced pressure. Purification by column chromatography on silica gel (DCM/MeOH 15/1) give compound **2** as yellow oil, 250 mg, 68% yield. $^1$H NMR ($CDCl_3$): $\delta$ = 8.64 (d, 1H, *J* = 4.6 Hz, $H_k$), 8.55 (d, 1H, *J* = 5.7 Hz, $H_f$), 8.49 (s, 1H, NH), 8.40 (d, 1H, *J* = 2.0 Hz, $H_g$), 8.33 (d, 1H, *J* = 8.0 Hz, $H_h$), 8.01 (dd, 1H, *J* = 2.0, 5.7 Hz, $H_e$), 7.83 (dt, 1H, *J* = 1.7, 7.7 Hz, $H_i$), 7.34 (ddd, 1H, *J* = 0.8, 4.8,7.4 Hz, $H_j$), 3.44 (t, 2H, *J* = 6.2 Hz, $CH_2$-$H_a$), 2.52 (t, 2H, *J* = 7.2 Hz, $CH_2$-$H_d$); 1.91 (m, 4H, $CH_2$-$H_b$, $CH_2$-$H_c$); $^{13}$C NMR ($CDCl_3$): $\delta$ = 172.2, 154.9, 153.4, 149.3, 148.3, 148.1, 137.4, 124.6, 121.4, 113.9, 110.8, 44.5, 36.7, 33.1, 23.6 ppm.

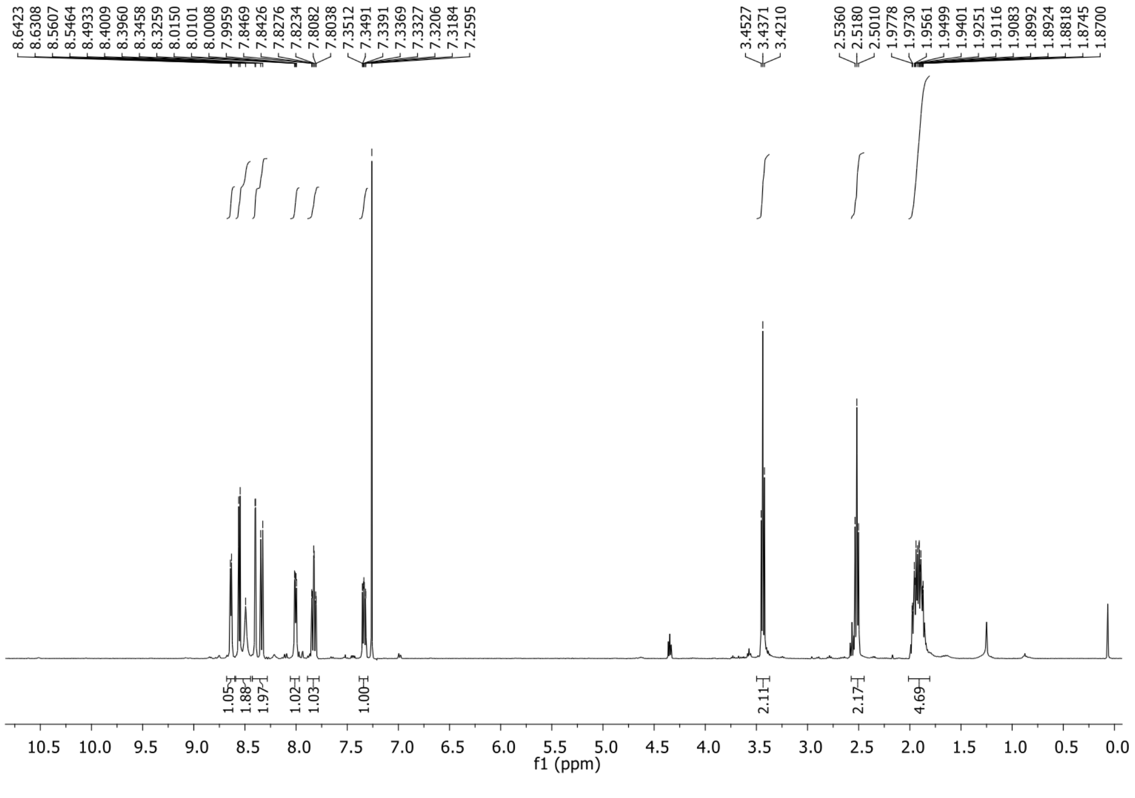

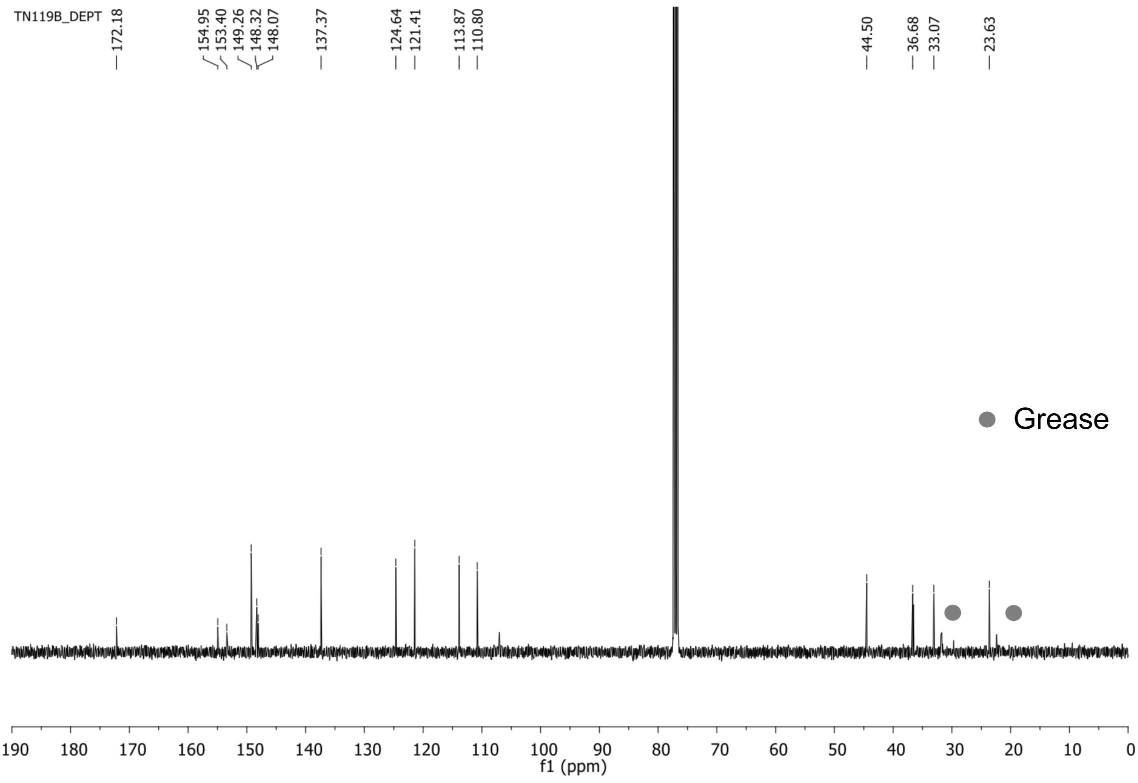


**N-([2,2'-bipyridin]-4-yl)-5-(2,4,6-trioxo-1,3,5-triazinan-1-yl)pentanamide**

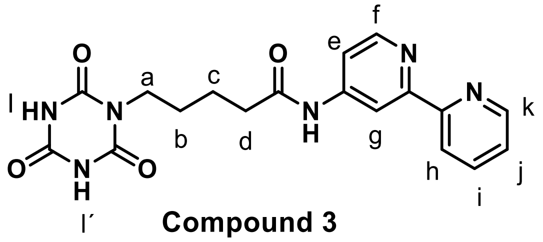

**Compound 3**

To a solution of cyanuric acid (309 mg, 2.39 mmol) in DMF (5mL) was added compound **2** (200 mg, 0.60 mmol) and 1,8-diazabicycloundec-7-ene (95 mg, 2.39 mmol). The reaction mixture was heated under 70 ºC overnight, poured into the water, and extracted with ethyl acetate. The organic layer was washed with water to eliminate the excess of cyanuric acid, dried with $MgSO_4$ and filtered. The solvent was removed under reduced pressure and the crude material was purified by column chromatography using DCM : MeOH 15:1 as a eluent to give **3** as a white solid, 596 mg, 25%.$^{1}$H NMR ([D6]dimethyl sulfoxide): $\delta$ = 11.35 (br s, 2H, NH-cy), 10,41 (s, 1H, NH-amide), 8.68 (d, 1H, $J$ = 4.0 Hz, $H_k$), 8.61 (d, 1H, $J$ = 2.0 Hz, $H_g$), 8.51 (s, 1H, $J$ = 5.5 Hz, $H_f$), 8.37 (d, 1H, $J$ = 8.0 Hz, $H_h$), 7.93 (dt, 1H, $J$ = 1.8, 7.7 Hz, $H_i$), 7.67 (dd, 1H, $J$ = 2.1, 4.0 Hz, $H_e$), 7.44 (dd, 1H, $J$ = 1, 4.8 Hz, $H_j$), 3.66 (t, 2H, $J$ = 6.6 Hz, $CH_2$-$H_a$), 2.40 (t, 2H, $J$ = 6.8 Hz, $CH_2$-$H_d$); 1.59 (m, 4H, $CH_2$-$H_b$, $CH_2$-$H_c$); $^{13}$C NMR ([D6]dimethyl sulfoxide): $\delta$ = 172.3, 156.1, 155.2, 150.1 (2C), 149.2, 148.6, 146.8, 137.2, 124.2, 120.4, 113.2, 109.8, 40.2, 36.1, 26.9, 21.9 ppm. MS m/z: calculated for $C_{18}H_{18}N_6O_4$ $[M+H]^+$ 383.1 found FAB 383.2.

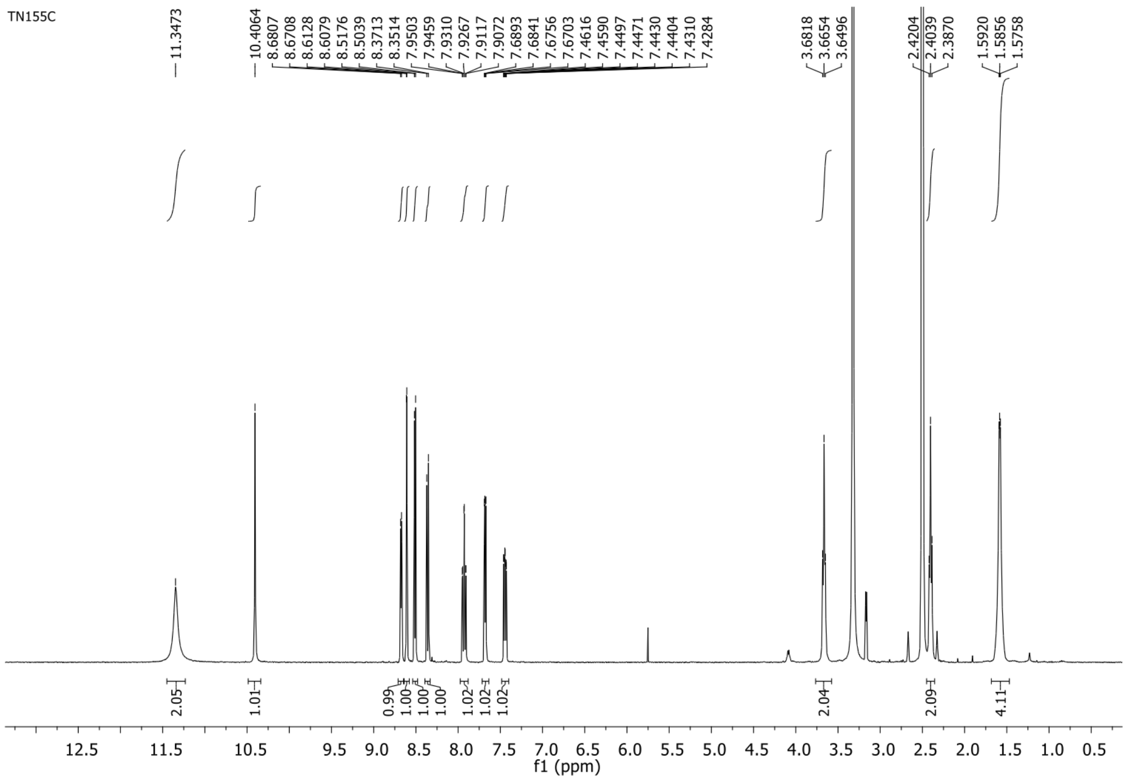
TN155C
11.3473
10.4064
8.6807
8.6708
8.6128
8.6079
8.5176
8.5039
8.3713
8.3514
7.9503
7.9459
7.9310
7.9267
7.9117
7.9072
7.6893
7.6841
7.6756
7.6703
7.4616
7.4590
7.4497
7.4471
7.4430
7.4404
7.4310
7.4284
3.6818
3.6654
3.6496
2.4204
2.4039
2.3870
1.5920
1.5856
1.5758
2.05
1.01
0.99
1.00
1.00
1.00
1.02
1.02
1.02
2.04
2.09
4.11
f1 (ppm)

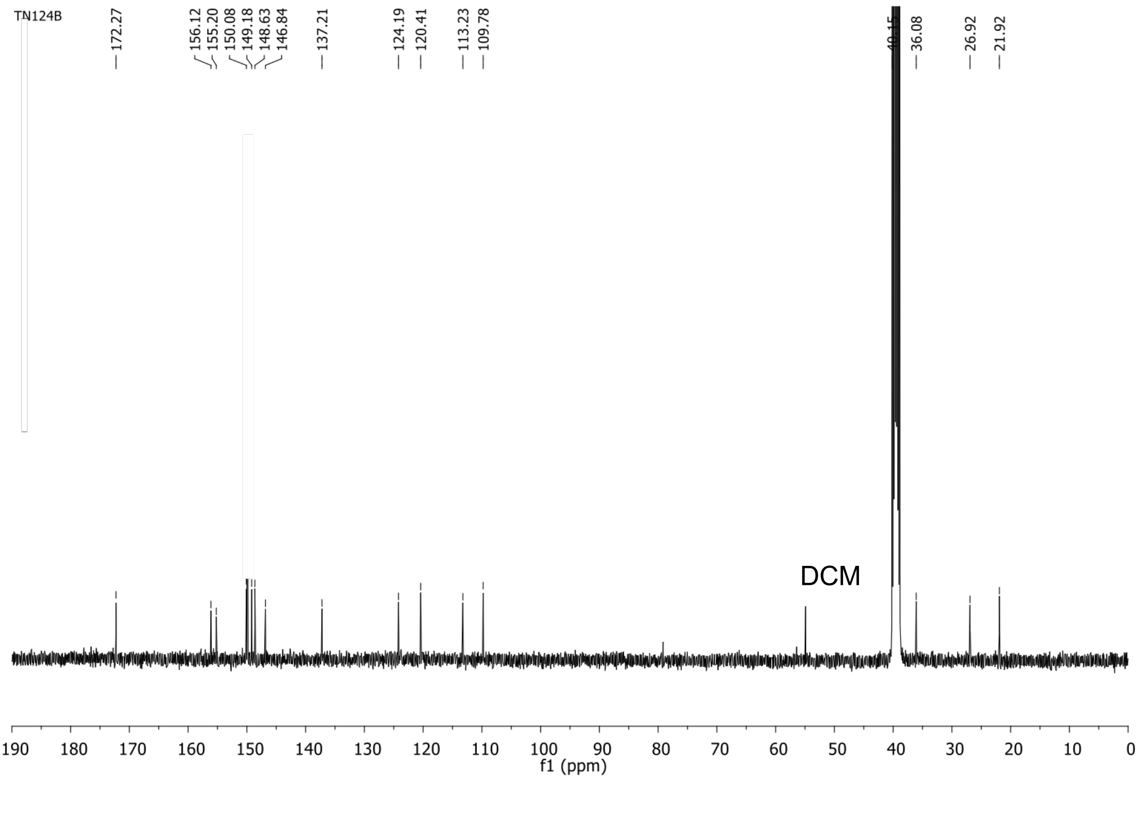
TN124B
172.27
156.12
155.20
150.08
149.18
148.63
146.84
137.21
124.19
120.41
113.23
109.78
36.08
26.92
21.92
DCM
f1 (ppm)

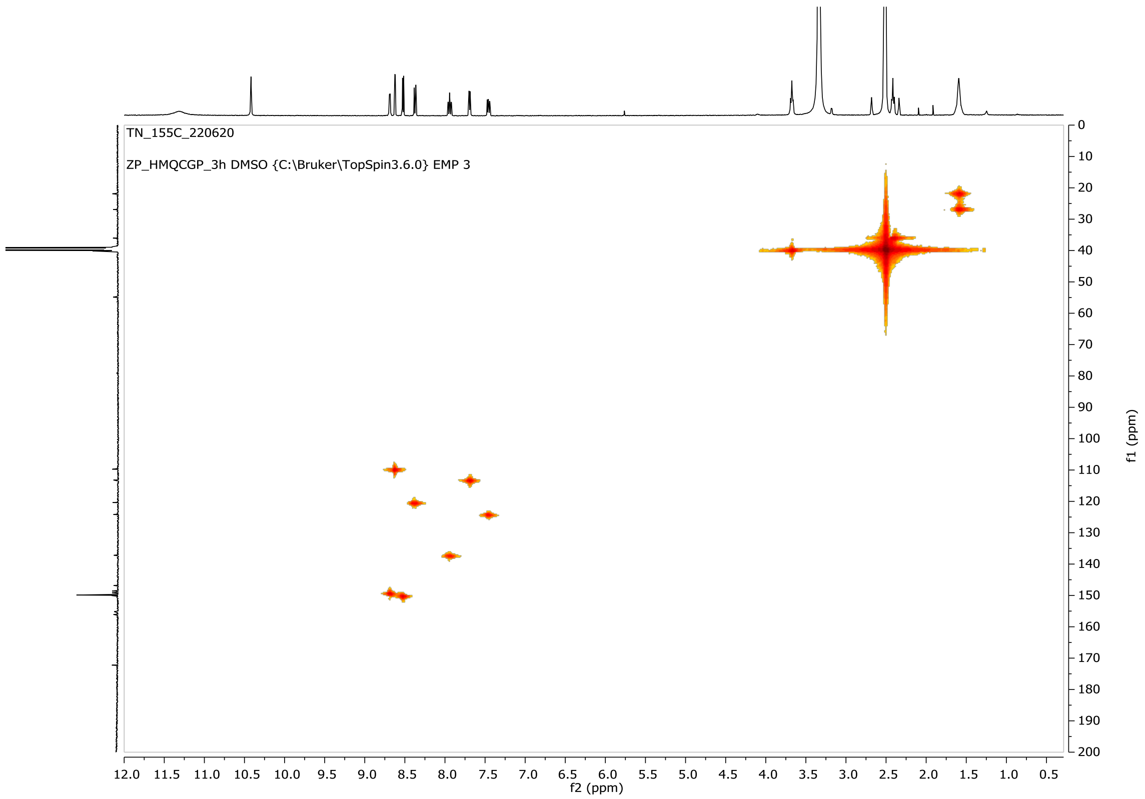
TN_155C_220620
ZP_HMQCGP_3h DMSO {C:\Bruker\TopSpin3.6.0} EMP 3
f1 (ppm)
f2 (ppm)

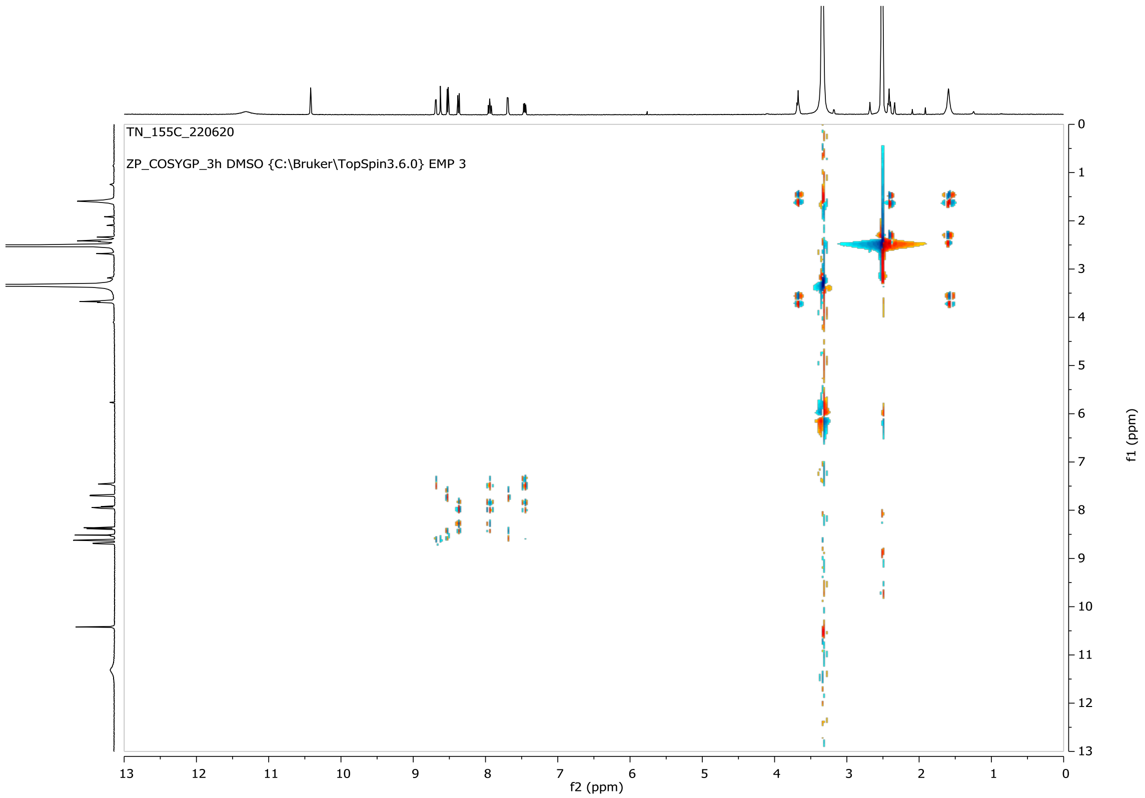
TN_155C_220620
ZP_COSYGP_3h DMSO {C:\Bruker\TopSpin3.6.0} EMP 3
f1 (ppm)
f2 (ppm)

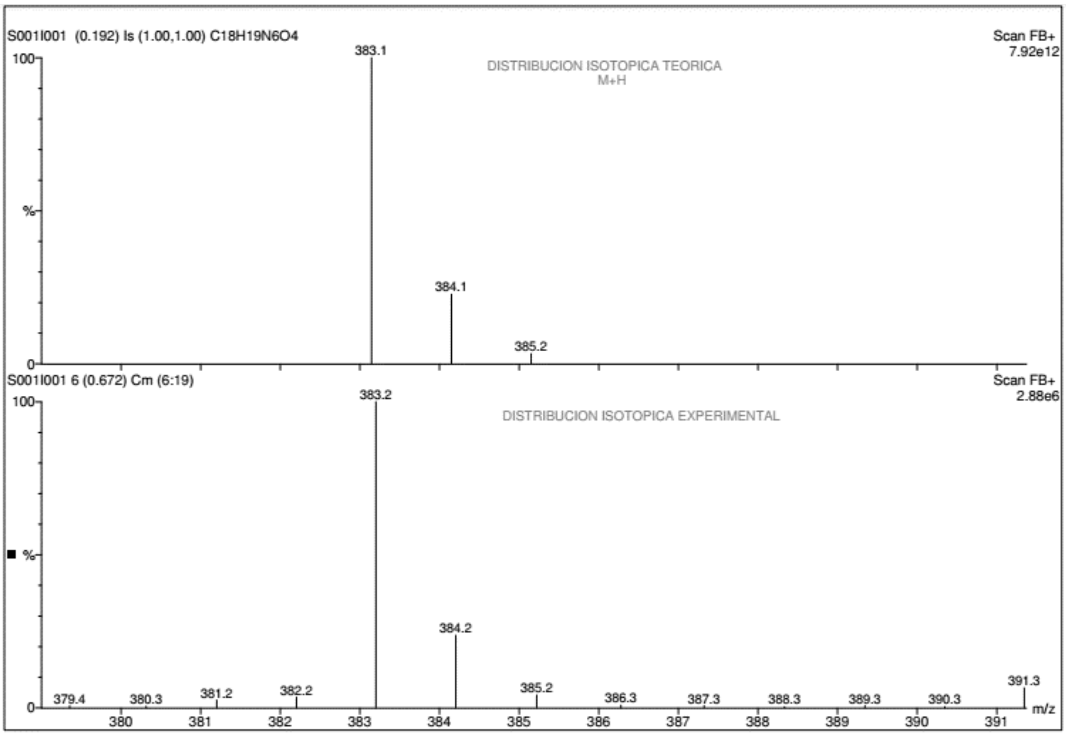


**(Bipyridine)$_2$-(bipyridine-*N*-([2,2'-bipyridin]-4-yl)-5-(2,4,6-trioxo-1,3,5-triazinan-1-yl) pentanamide) ruthenium hexafluorophosphate**

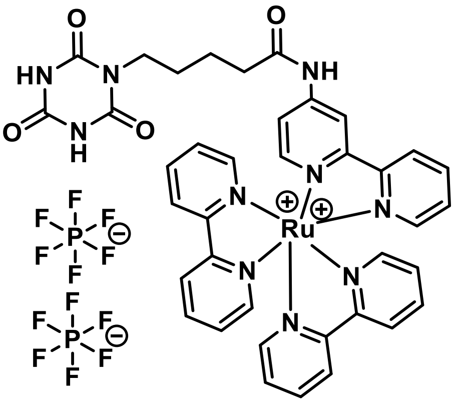

**Compound 4**

[Ru(bpy)$_2$Cl$_2$] (54 mg, 0.11 mmol) was reacted with silver nitrate (37 mg, 0.22 mmol) in $CH_3OH$ (5.4 mL) for 3 hours at room temperature under an Ar atmosphere. The suspension was filtered in order to remove the silver salt, and the filtrate was added to compound **3** (47 mg, 0.12 mmol). The solution was heated at reflux in the dark overnight under an Ar atmosphere. The reaction mixture was allowed to reach room temperature and the solvent was evaporated. The remaining solid was re-dissolved in a minimum amount of $CH_3OH$, and the desired compound was precipitated by dropwise addition of a saturated aqueous solution of ammonium hexafluorophosphate. The precipitate was filtered, washed with water then $Et_2O$ and dried under vacuum to yield 100 mg (85%) of the desired hexafluorophosphate ruthenium complex (compound **4**) as a orange solid.

$^{1}$H NMR ($CD_3CN$): δ = 8.95 (br s, 1H), 8.83 (br s, 1H), 8.81 (d, 2H, *J* =2.0 Hz, Ar-H), 8.47 (d, 4H, *J* = 8.2 Hz, Ar-H), 8.36 (d, 1H, *J* = 6.7 Hz, Ar-H), 8.03 (m, 4H, Ar-H), 7.79 (d, 1H, *J* = 5.6 Hz, Ar-H), 7.71 (m, 3H, Ar-H), 7.41 (m, 6H, Ar-H), 3.74 (t, 2H, *J* = 5.3 Hz, $CH_2$), 2.45 (t, 2H, *J* = 6.8 Hz, $CH_2$), 1.65 (m, 4H, $2CH_2$). $^{13}$C NMR ($CD_3CN$): δ = 174.1, 158.3, 158.2 (2C), 158.1 (2C), 158.0, 152.8, 152.8, 152.7, 152.7 (2C), 152.6, 152.6, 150.6 (2C), 148.8, 148.5, 138.8, 138.6 (3C), 128.6, 128.5, 128.5 (3C), 125.2, 125.2 (2C), 125.1, 118.3, 117.4, 114.7, 41.7, 37.3, 27.9, 22.7 ppm. MS m/z: calculated. for $C_{38}H_{34}F_6N_{10}O_4PRu$ $[M+PF_6]^+$ 941.1 found MALDI-TOF 941.2; calculated for $C_{38}H_{34}N_{10}O_4Ru$ $[M]^+$ 795.2 found MALDI-TOF 795.2.

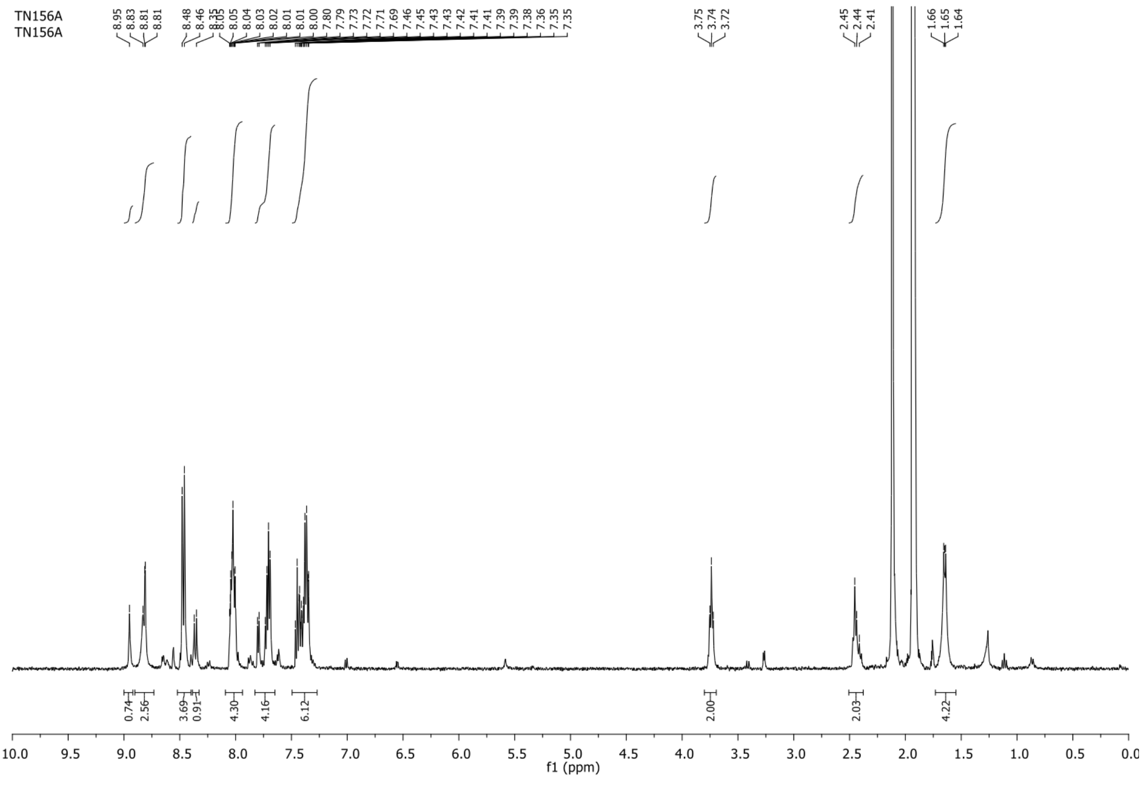

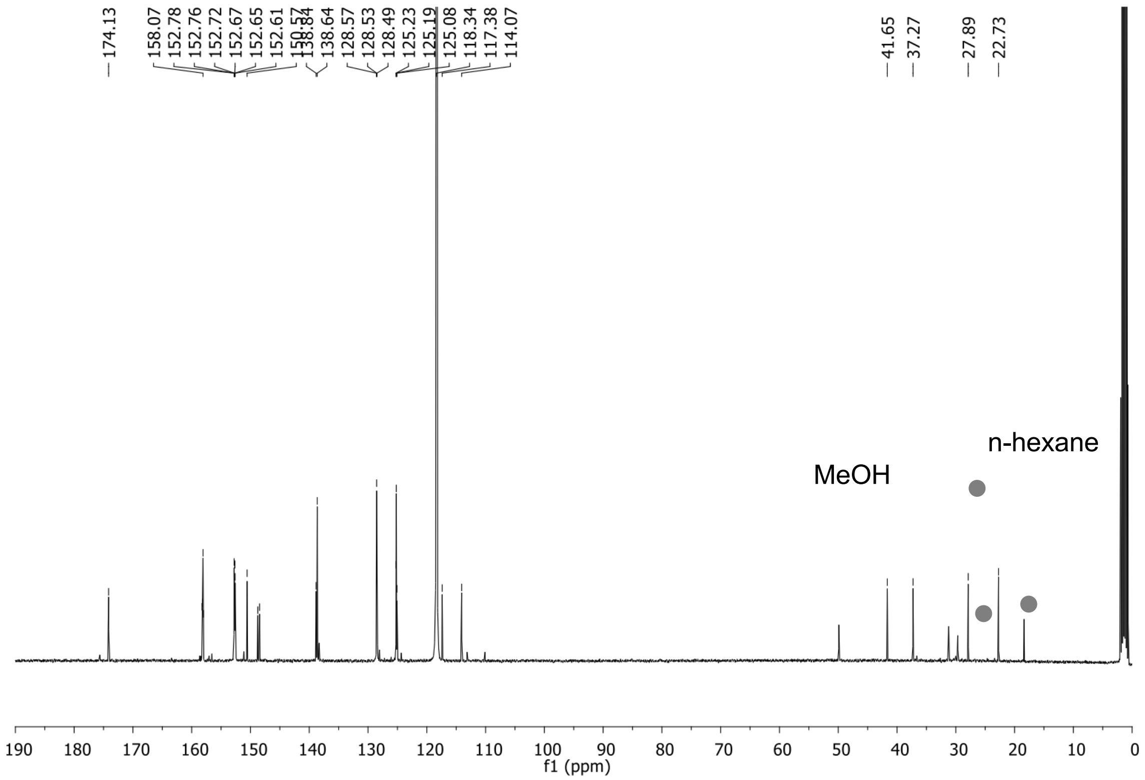
174.13
158.07
152.78
152.76
152.72
152.67
152.65
152.61
150.57
138.84
138.64
128.57
128.53
128.49
125.23
125.19
125.08
118.34
117.38
114.07
41.65
37.27
27.89
22.73
MeOH
n-hexane
f1 (ppm)

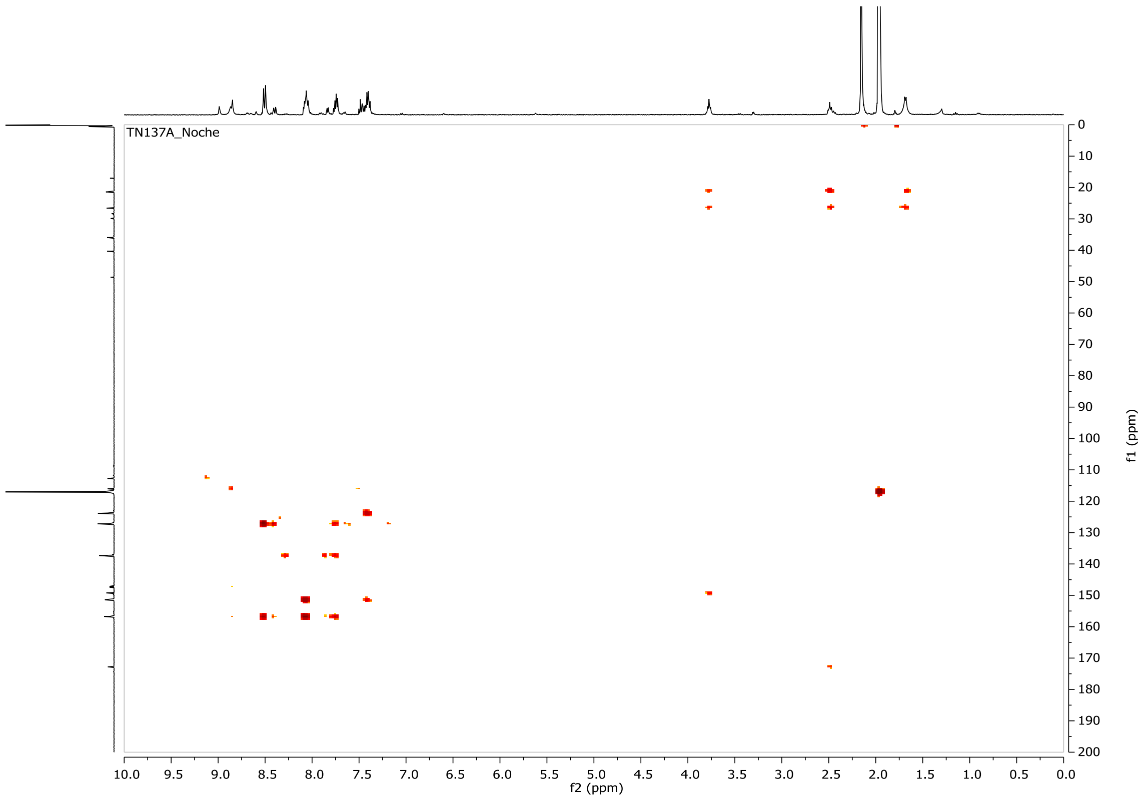
TN137A_Noche
f1 (ppm)
f2 (ppm)

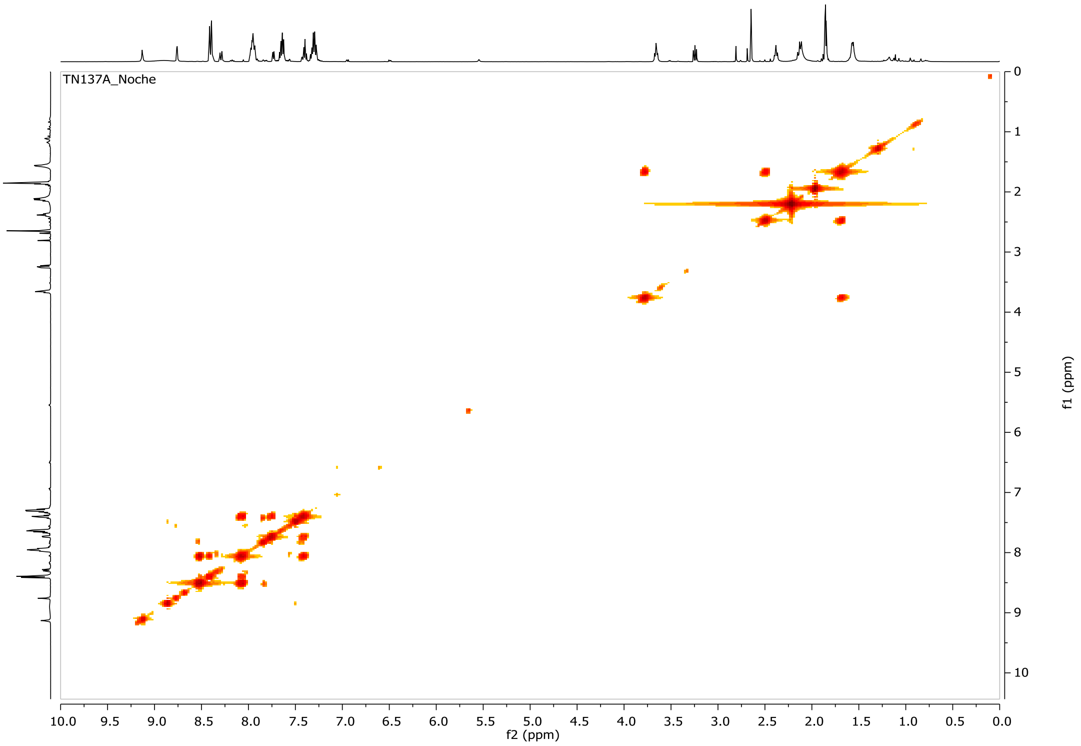
TN137A_Noche
f2 (ppm)
f1 (ppm)

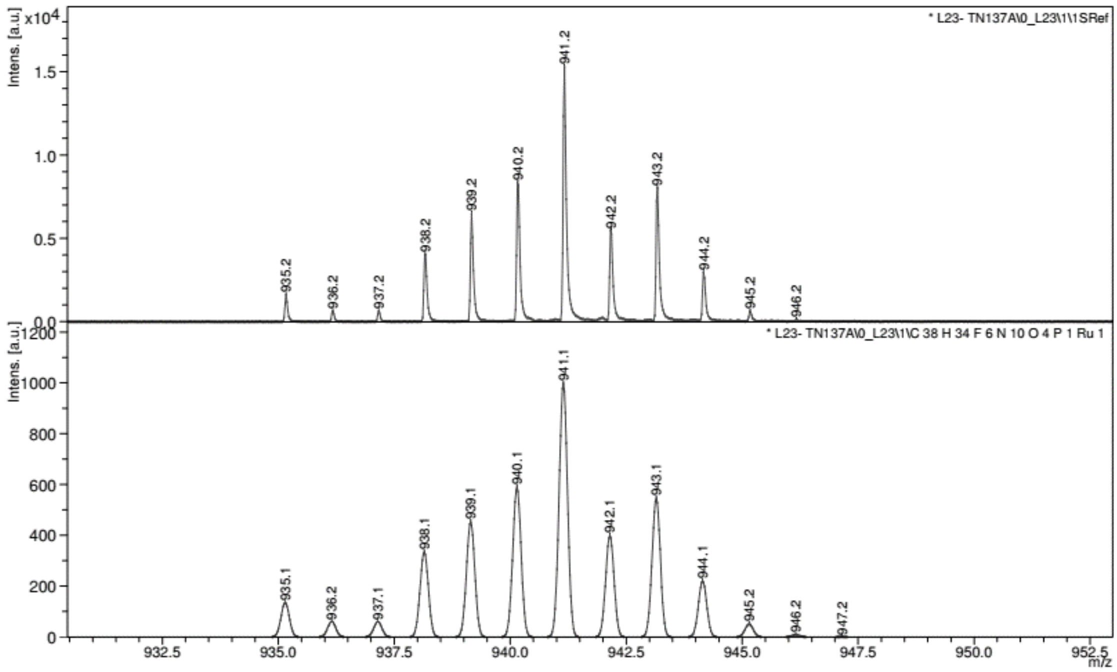
* L23- TN137A\0_L23\1\1SRef
* L23- TN137A\0_L23\1\C 38 H 34 F 6 N 10 O 4 P 1 Ru 1
Intens. [a.u.]
m/z

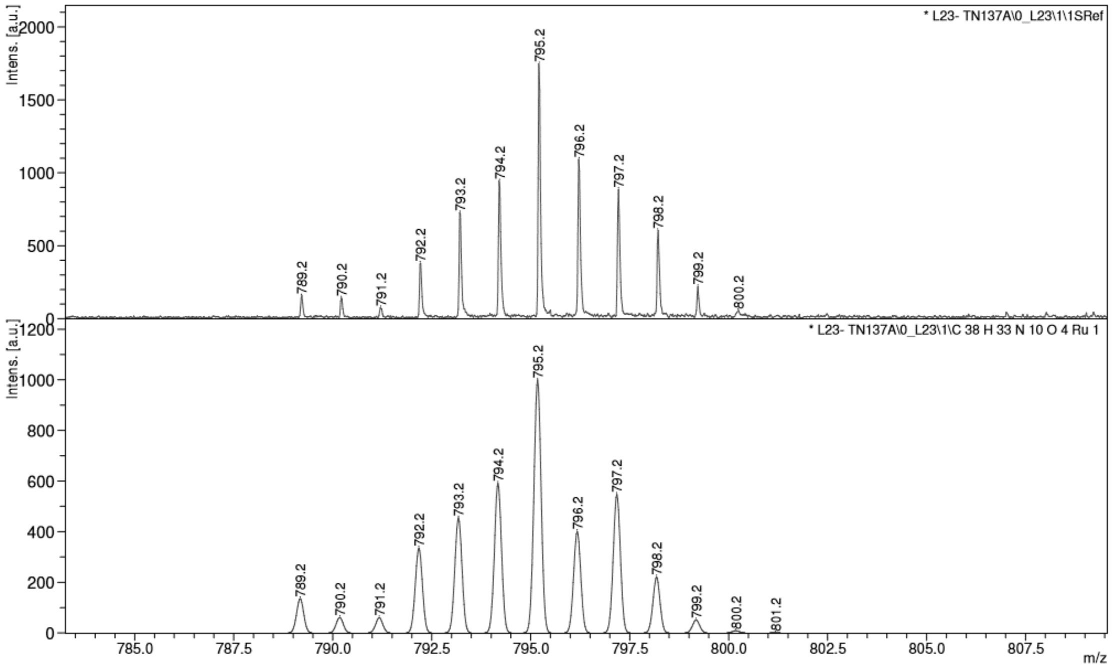
* L23- TN137A\0_L23\1\1SRef
Intens. [a.u.]
789.2
790.2
791.2
792.2
793.2
794.2
795.2
796.2
797.2
798.2
799.2
800.2
* L23- TN137A\0_L23\1\C 38 H 33 N 10 O 4 Ru 1
801.2
m/z

## 2b. HR synthesis

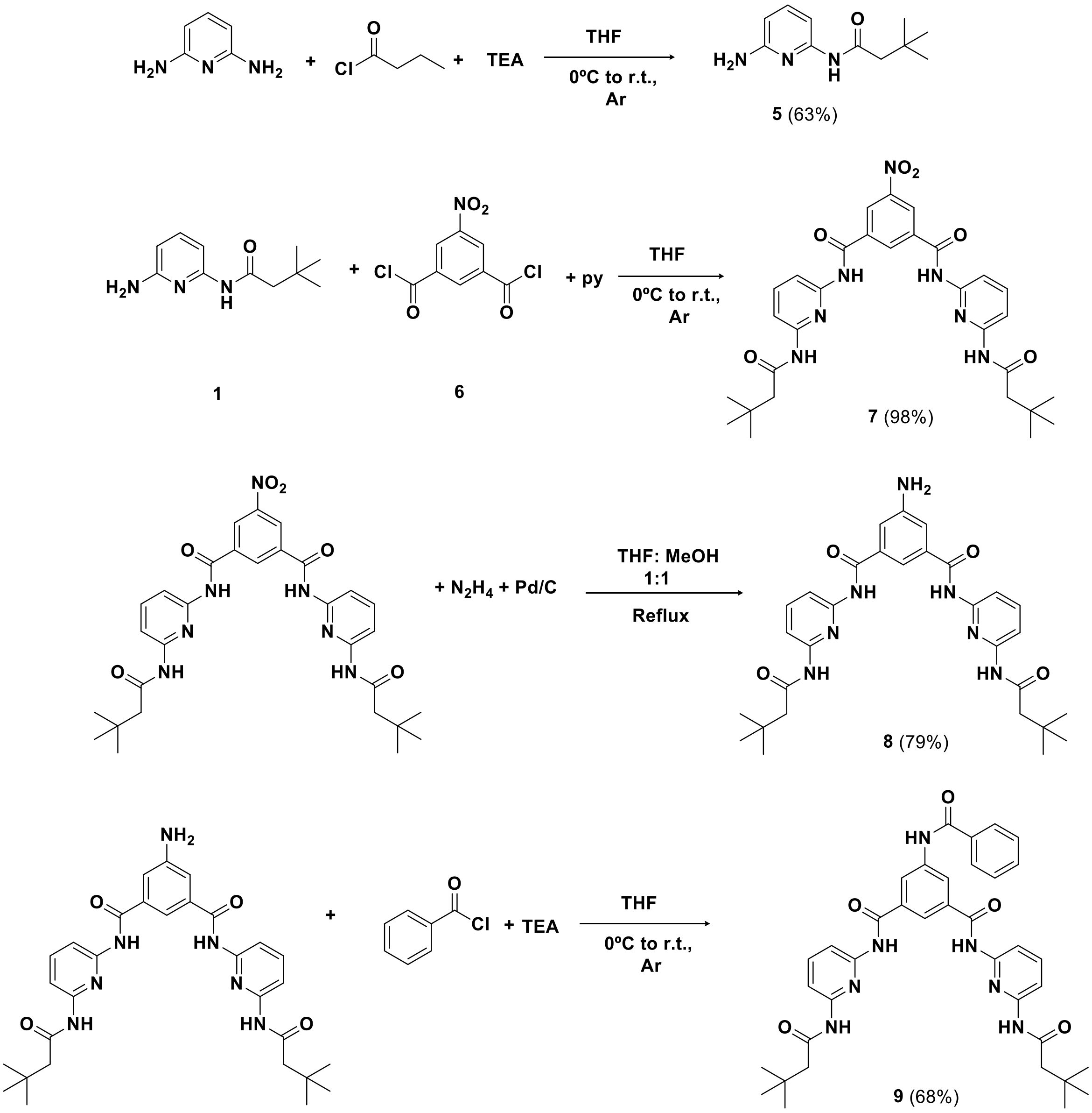


The 5-amino-*N,N'*-bis[6-(3,3-dimethylbutyrylamino) pyridin-2-yl] isophthalamide, compound **8**, was synthesized as described by Dirksen et al.[5] The experimental procedures followed are explained bellow.

### *N*-(6-Aminopyridin-2-yl)-3,3-dimethylbutyramide

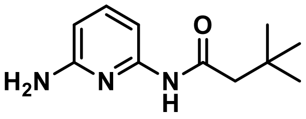

**Compound 5**

A solution of 3,3-dimethyl-butyryl chloride (3 g, 22.3 mmol) in dry THF (12 mL) was added to a solution of 2,6-diaminopyridine (2.43 g, 22.3 mmol) and triethylamine (3.1 mL, 22.3

mmol) in dry THF (25 mL) at 0ºC under an argon atmosphere over a period of 2 h. The solution was stirred for 60h at room temperature, the residue filtered off, and the solvent removed under reduced pressure. Purification by column chromatography on silica gel (DCM/ethyl acetate 4/1 as eluent) gave a colorless solid (2.9 g, 63%); $^1$H NMR ($CDCl_3$): $\delta$ = 7.77 ppm (br s, 1H, CONH), 7.55 (d, 1H, $J$ = 7.9 Hz, Hpy), 7.42 (t, 1H, $J$ = 8.0 Hz; Hpy), 6.22 (d, 1H, $J$ = 7.9 Hz, Hpy), 4.34 (br s, 2H; $NH_2$), 2.17 (s, 2H; (**$CH_2$**$C(CH_3)_3$), 1.05 (s, 9H; $(C(CH_3)_3)$ ppm.

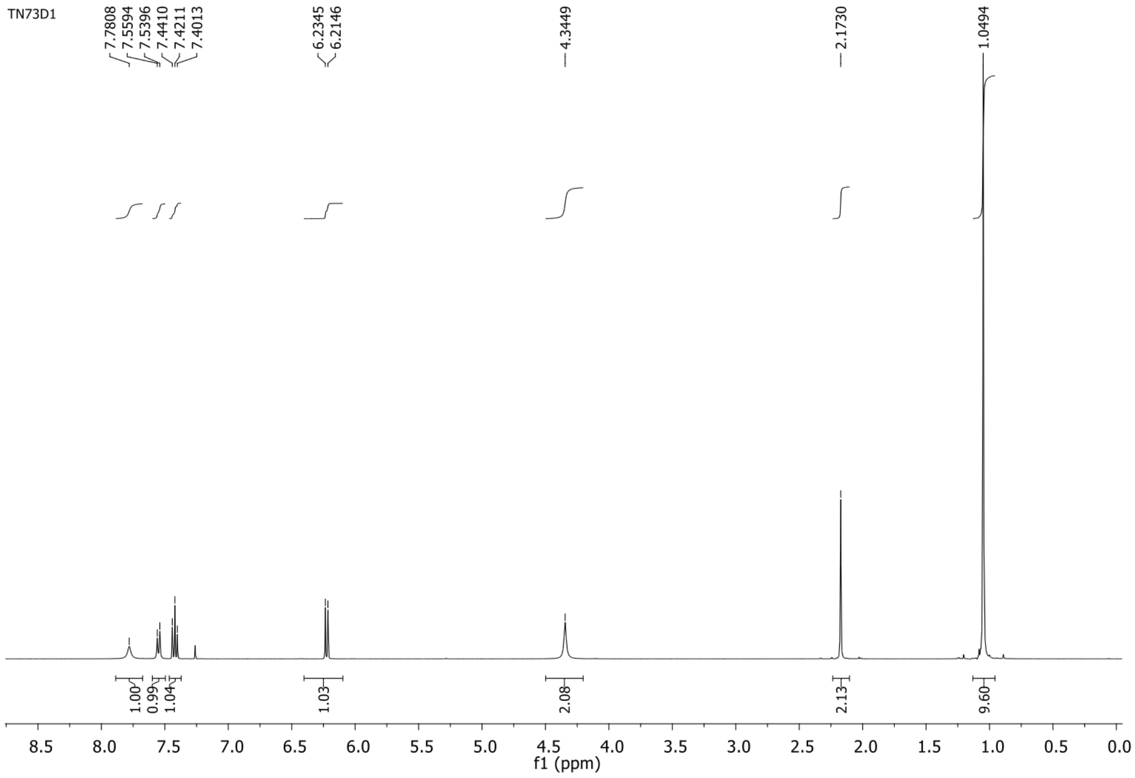


**5-Nitroisophthaloyl dichloride**

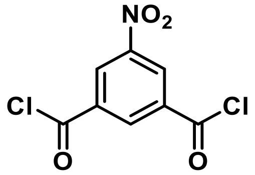


**Compound 6**

A solution of 5-nitroisophthalic acid (3.0 g, 14.0 mmol) in thionyl chloride (5 mL) and *N,N'*-dimethylformamide (five drops) was refluxed for 6 h under dry conditions with subsequent vacuum distillation of the thionyl chloride excess. The residue was dried under high vacuum and yielded a white solid (2.6 g, 98%): $^1$H NMR ([D6]dimethyl sulfoxide): $\delta$ = 8.77 (d, $J$ = 1.52 Hz ,2H; Ar-H), 8.74 (t, $J$ = 1.52 Hz, 1H; Ar-H) ppm.

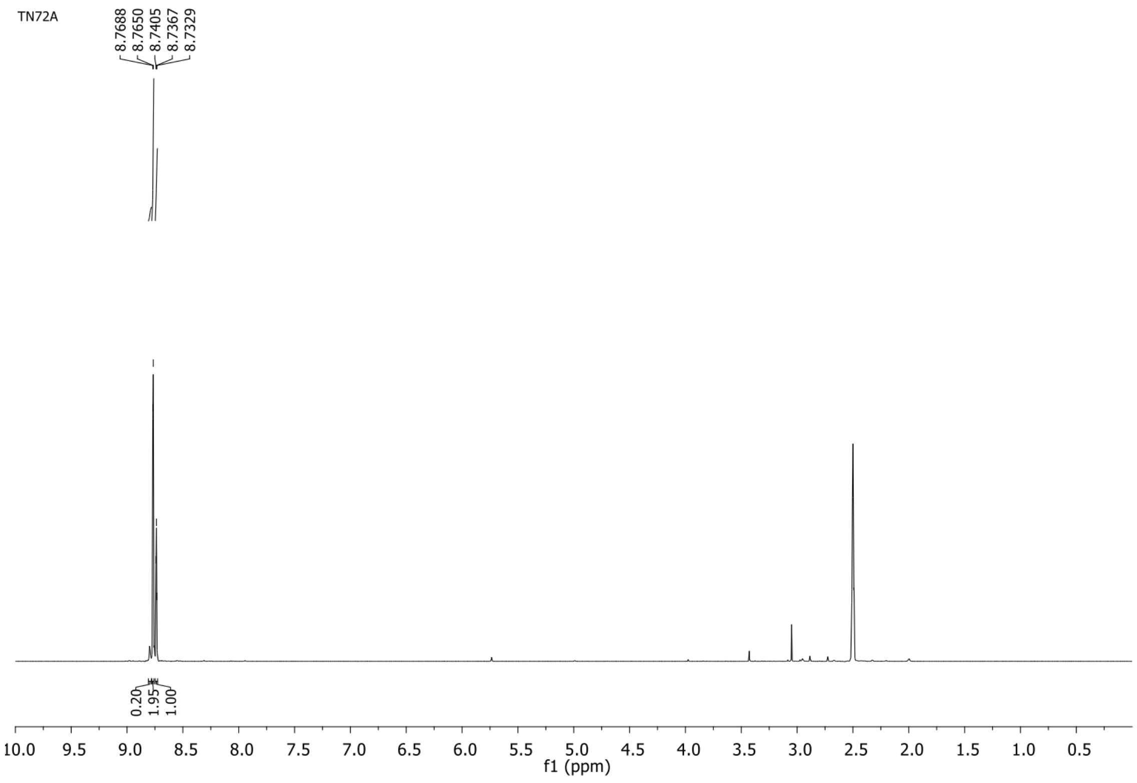


***N,N'*-Bis[6-(3,3-dimethylbutyrylamino)pyridin-2-yl]-5-nitro-isophthalamide**

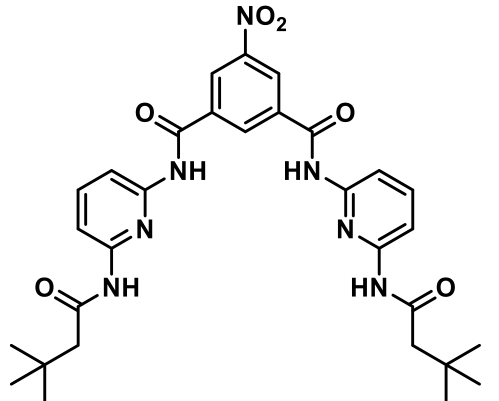


**Compound 7**

A solution of diacid dichloride **6** (0.9 g, 3.62 mmol) in dry THF (15 mL) was added dropwise to a solution of monosubstituted diaminopyridine **5** (1.5 g, 7.24 mmol) and triethylamine (1 mL, 7.24 mmol) in dry THF (15 mL) at 0ºC under an argon atmosphere. The solution was stirred at r.t. for 12 h, the residue was filtered off, and the solvent removed under reduced pressure. Purification by column chromatography on silica gel (DCM/ethyl acetate 10/1-3/1 as eluent) gave a yellowish solid (2.1 g, 98%): $^{1}$H NMR ($CDCl_3$): $\delta$ = 8.91 (br d, *J* = 1.3 Hz, 2H; CONH), 8.80 (br t, *J* = 1.3 Hz, 1H; Ar-H), 8.46 (br s, 2H; Ar-H), 8.02 (t, *J* = 8.0 Hz, 4H; Hpy), 7.79 (t, *J* = 8.0 Hz, 2H; Hpy), 7.66 (br s, 2H; CONH), 2.28 (s, 4H; $COCH_2$), 1.13 (s, 18H; $C(CH_3)_3$) ppm.

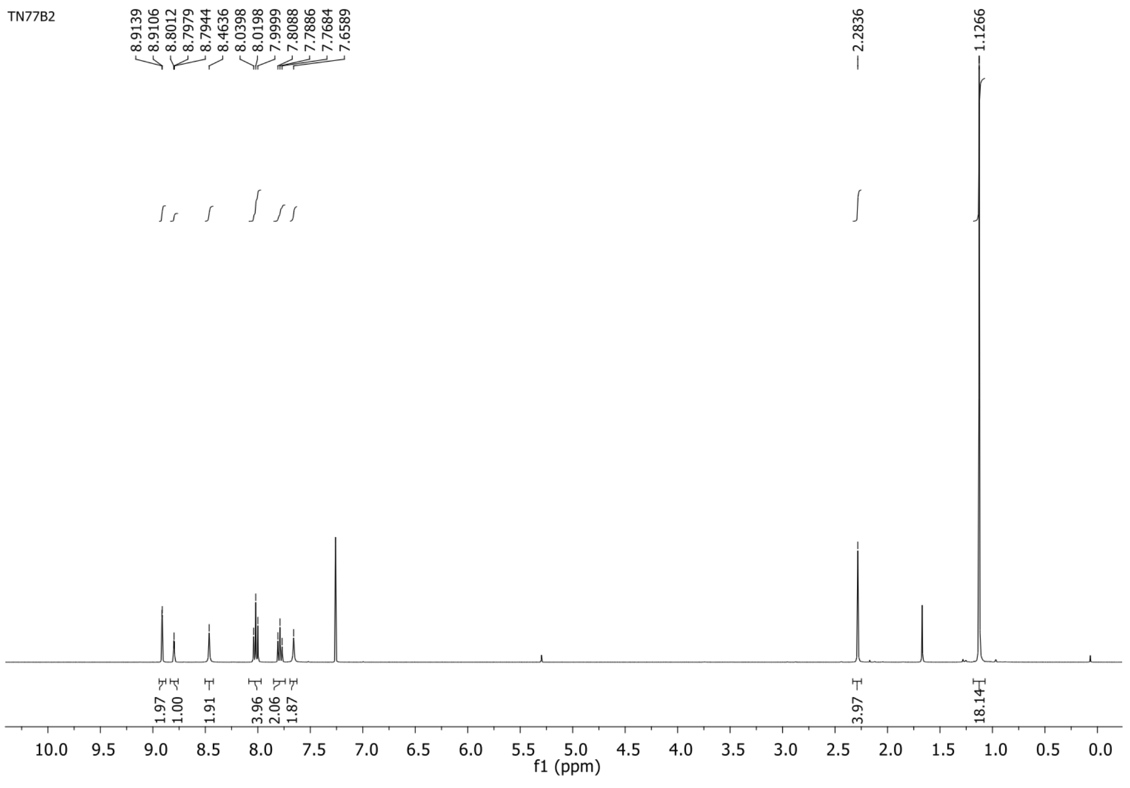


**5-Amino-*N,N'*-bis[6-(3,3-dimethylbutyrylamino)pyridin-2-yl]isophthalamide**

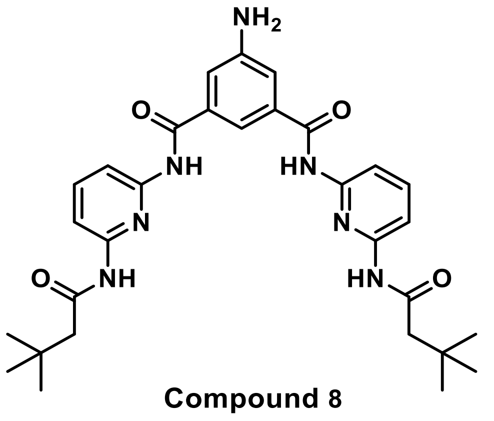


**Compound 8**

To a solution of compound **7** (0.4 g, 0.68 mmol) in dry THF (34 mL) and MeOH (34 mL) under argon atmosphere, 10% Pd-C (400 mg) was added and the reaction mixture was heated to 70 ºC. Then hydrazine (1.1 mL, 20.4 mmol) was added to the reaction mixture, whih was heated under reflux for 4h. Once the reaction was completed (TLC). The catalyst was filtered off over celite and the solvent removed under reduced pressure to give a yellowish solid (0.3 g, 79%): $^1$H NMR ($CDCl_3$): $\delta$ = 8.47 ppm (s, 2H; CONH), 8.10 (s, 2H; CONH), 7.93 (br t, 4H; Hpy), 7.66 (m, 3H; 2H-py, Ar-H), 4.08 (br s, 2H, $NH_2$), 2.26 (s, 4H; $2CH_2$), 1.11 (s, 18H; $C(CH_3)_3$) ppm.

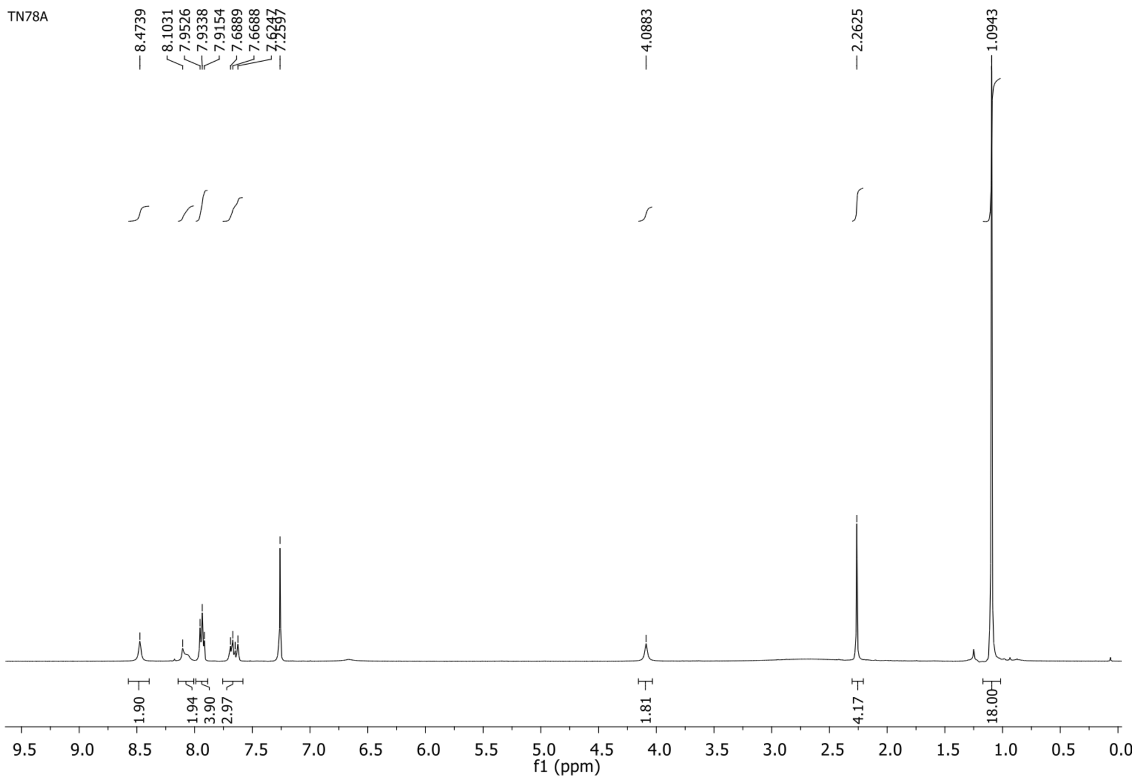


**5-benzamido-*N,N´*-bis(6-(3,3-dimethylbutyrylamino)pyridin-2-yl)isophthalamide**

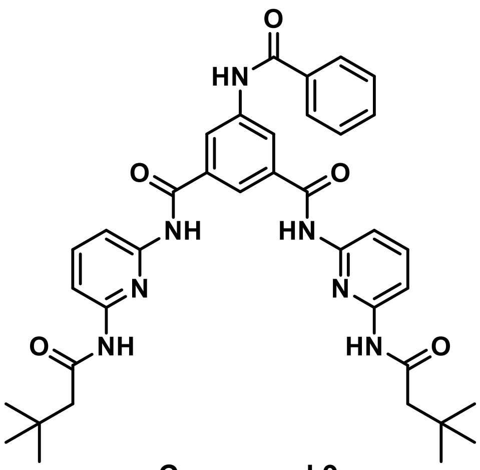


**Compound 9**

A solution of benzoyl chloride (21 mg, 0.15 mmol) in dry THF (1 mL) was added dropwise to a solution of compound **8** (50 mg, 0.09 mmol) and triethylamine (16 μL, 0.12 mmol) in dry THF (2 mL) at 0ºC under an argon atmosphere. The solution was stirred at r.t. for 12 h, the residue filtered off, and the solvent removed under reduced pressure. Purification by column chromatography on silica gel (DCM/MeOH 15/1 as eluent) gave a yellowish oil (40 mg, 68%): $^1$H NMR ($CDCl_3$): δ = 9.24 (br s, 1H), 8.97 (br s, 2H), 8.48 (br s, 2H), 8.40 (br s, 2H), 7.99 (m, 3H), 7.90 (d, 2H, *J* = 8.0 Hz), 7.76 (d, 2H, *J* = 7.8 Hz), 7.53 (m, 3H), 7.41 (t, 2H, *J* = 7.5 Hz), 2.42 (s, 4H), 1.23 (s, 18H); $^{13}$C NMR ($CDCl_3$): δ = 171.7 (2C), 170.1, 167.0, 163.9, 150.1 (2C), 148.8 (2C), 140.6 (2C), 139.2, 135.0, 133.6, 132.6

(2C), 130.1, 128.8 (2C), 128.3, 127.5 (2C), 122.2, 122.0, 110.6 (2C), 109.4 (2C), 50.9 (2C), 31.5 (2C), 29.8 (6C) ppm.

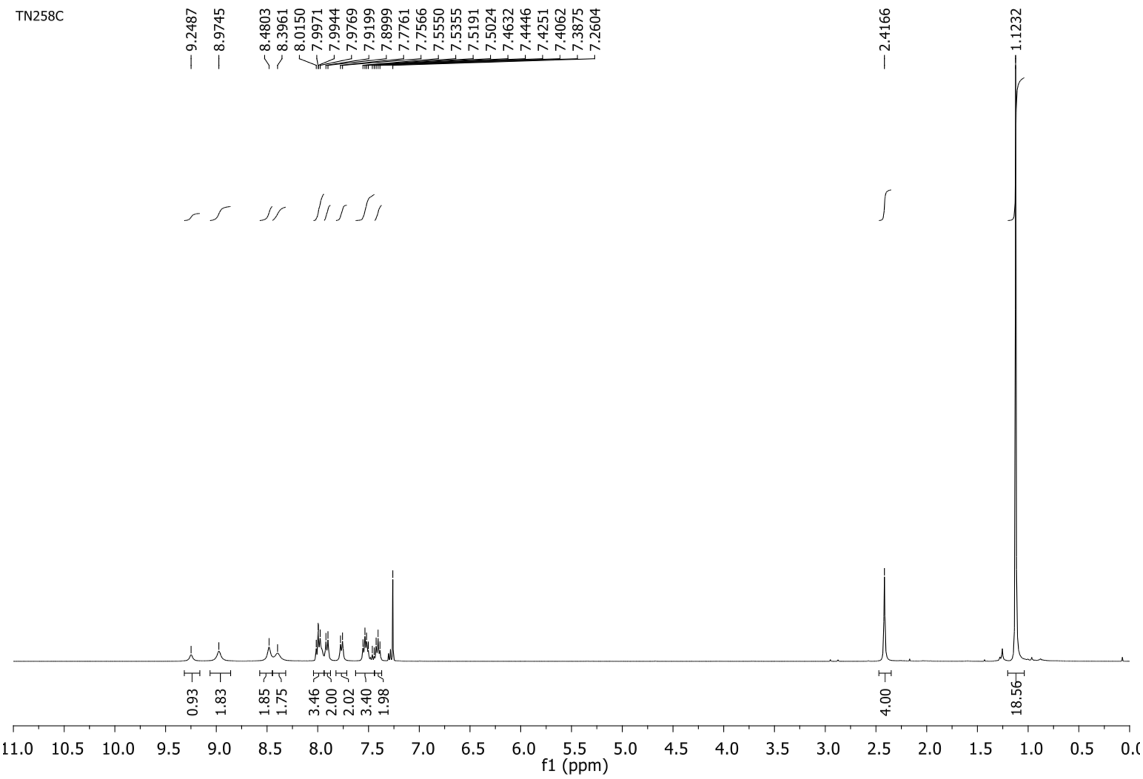


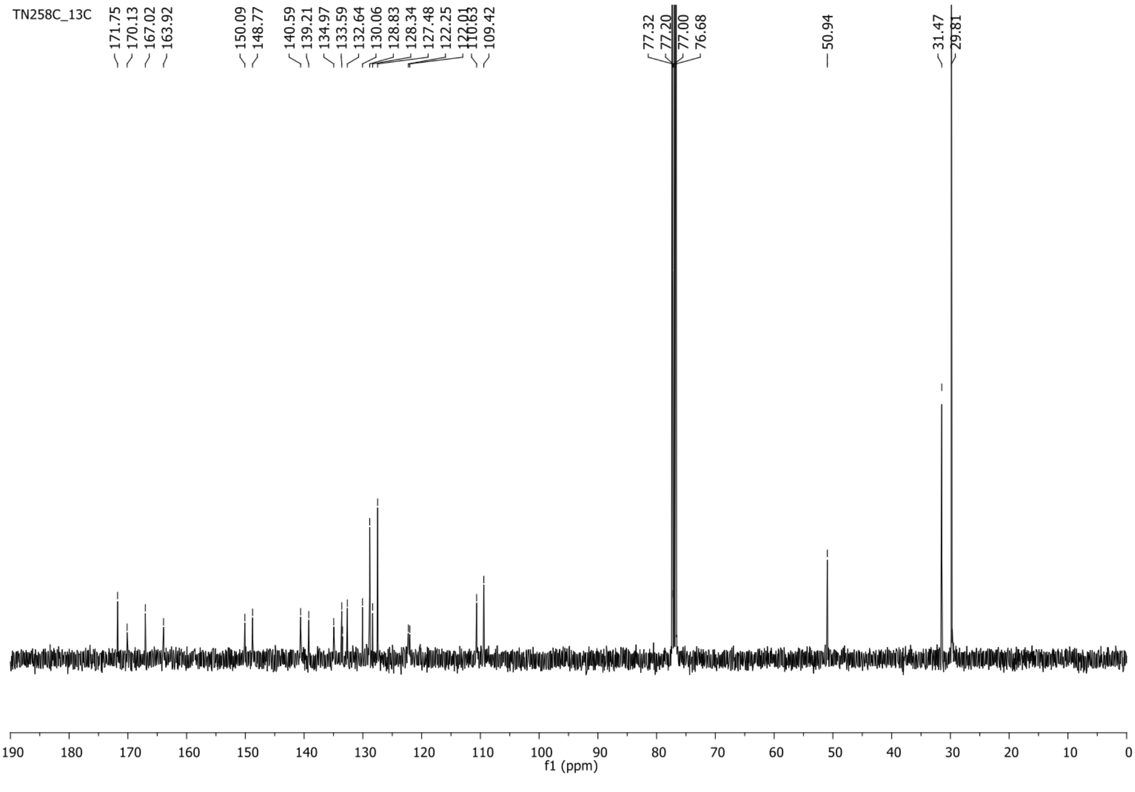

## 3. Synthesis of Ammonium derivative (Am)

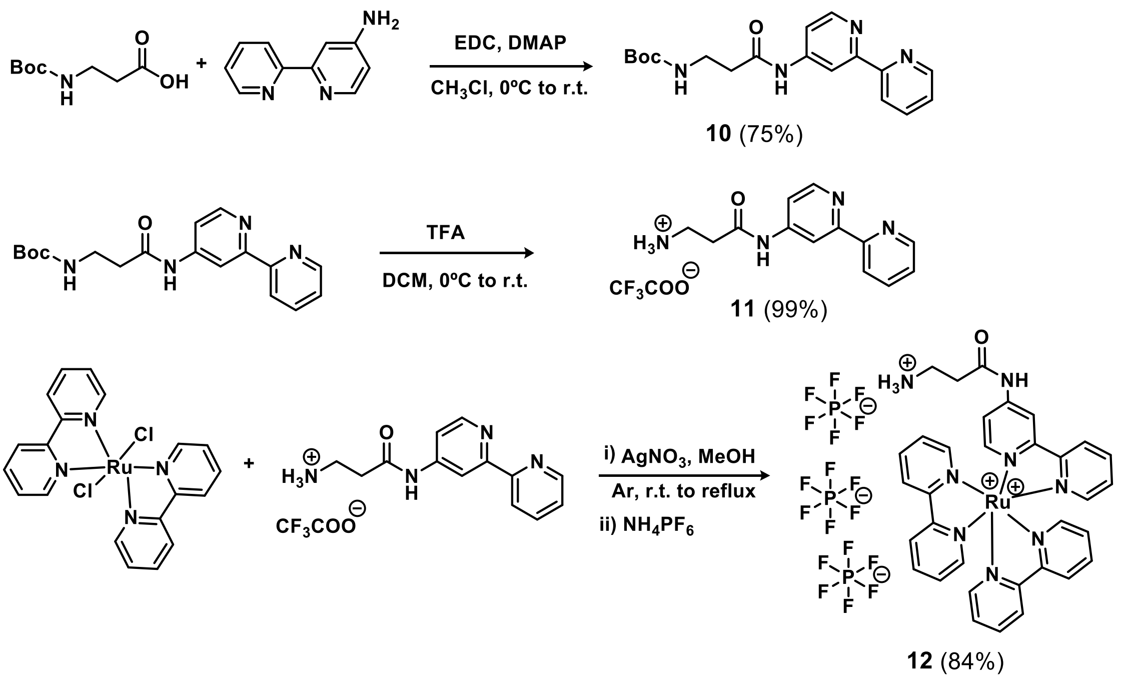


### *tert*-butyl (3-([2,2'-bipyridin]-4-ylamino)-3-oxopropyl)carbamate

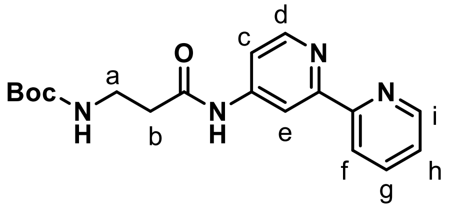

**Compound 10**

3-((*tert*-butoxycarbonyl) amino)propanoic acid (133 mg, 0.70 mmol) was dissolved in $CHCl_3$ (6 mL) and the solution was cooled to 0ºC. EDCI.HCl (168 mg, 0.88 mmol) and DMAP (122 mg, 0.99 mmol) were added. The reaction mixture was allowed to stir at room temperature for 30 minutes. A solution of [2,2'-bipyridin]-4-amine (100 mg, 0.58 mmol) in $CHCl_3$ (4 mL) was added to the activated acid. The reaction mixture was stirred overnight, concentrated under reduced pressure and the crude material was purified by column chromatography (eluent: DCM/ MeOH 50/1-40/1) to furnish product **10** as colorless oil, 150 mg, 75%. $^1H$ NMR ($CDCl_3$): $\delta$ = 8.70 (s, 1H, NH), 8.63 (d, 1H, $J$ = 4.8 Hz, $H_i$), 8.53 (d, 1H, $J$ = 5.5 Hz, $H_d$), 8.40 (br s, 1H, $H_e$), 8.33 (d, 1H, $J$ = 8.0 Hz, $H_f$), 7.78 (dt, 1H, $J$ = 1.8, 7.7 Hz, $H_g$), 7.73 (m, 1H, $H_c$), 7.28 (ddd, 1H, $J$ = 0.8, 4.8, 7.5 Hz, $H_h$), 5.33 (br s, 1H, NH), 3.48 (q, 2H, $J$ = 6.2 Hz, $CH_2$-$H_a$), 2.64 (t, 2H, $J$ = 7.0 Hz, $CH_2$-$H_b$);

1.42 (s, 9H, $3CH_3$-Boc); $^{13}C$ NMR : $\delta$ = 170.7, 157.1, 156.4, 155.7, 150.2, 149.1, 146.0, 136.9, 123.9, 121.1, 113.7, 110.9, 79.8, 37.8, 36.3, 28.4 ppm.

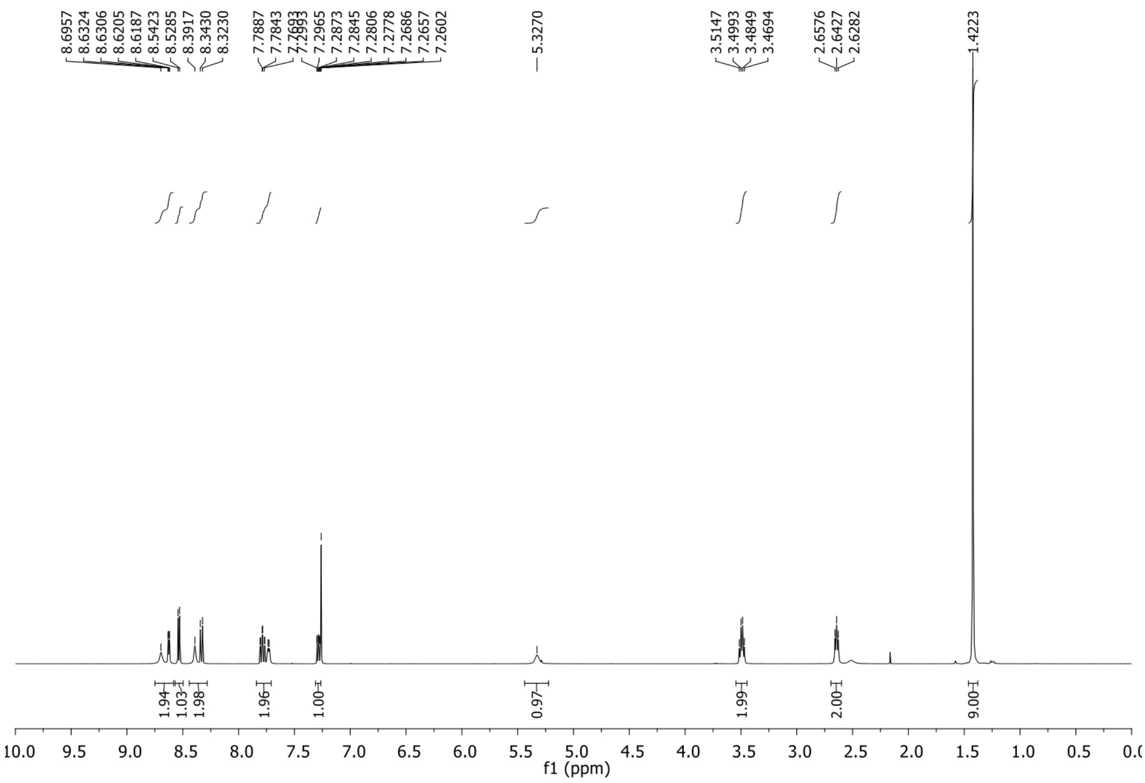

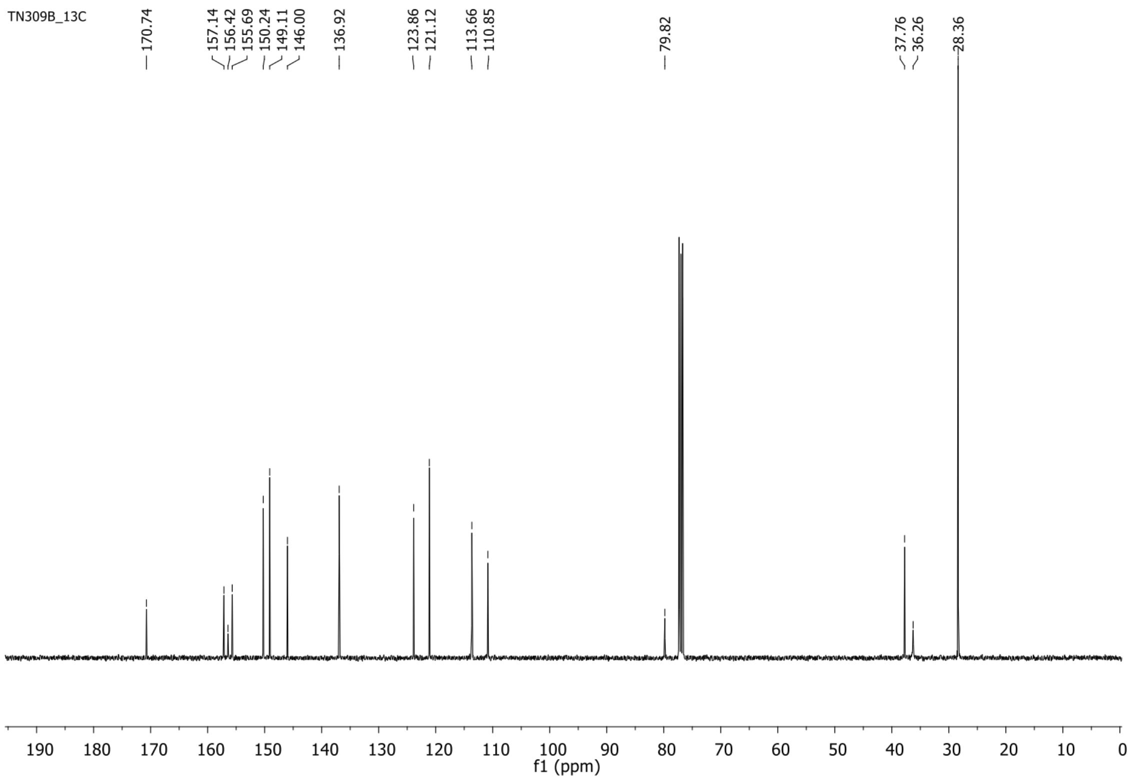


**3-([2,2'-bipyridin]-4-ylamino)-3-oxopropan-1-aminium 2,2,2-trifluoroacetate**

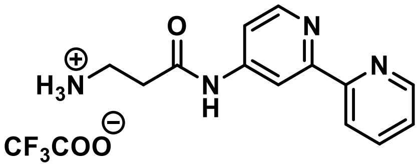

**Compound 11**

A solution of compound **10** (60 mg, 0.18 mmol) in DCM (1.5 mL) was added TFA (0.1 mL) at 0ºC. The reaction was stirred at room temperature for 3h until it was completed (TLC). The reaction mixture was concentrated under reduced, then DCM was added and the organic layer was washed twice with brine, dried over $Na_2SO_4$, concentrated under reduced pressure and dried under high vacuum to give compound **11** as a yellowish oil, quantitative yield. The crude material was used directly in the next step reaction.

**(Bipyridine)$_2$-(bipyridine-*N*-([2,2'-bipyridin]-4-yl)-5-(2,4,6-trioxo-1,3,5-triazinan-1-yl) pentanamide) ruthenium hexafluorophosphate: <u>Am</u>**

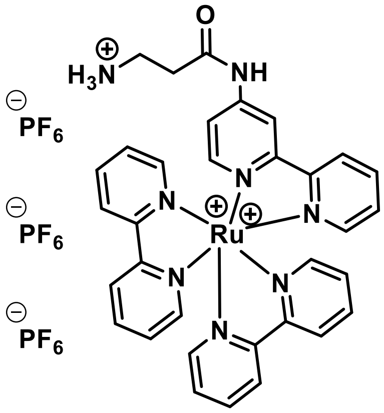


**Compound 12**
**Am**

[$Ru(bpy)_2Cl_2$] (54 mg, 0.11 mmol) was reacted with silver nitrate (37 mg, 0.22 mmol) in $CH_3OH$ (5.4 mL) for 3 hours at room temperature under an argon atmosphere. The suspension was filtered in order to remove the silver salt, and the filtrate was added to compound **11** (44 mg, 0.12 mmol). The solution was stirred at room temperature in the dark overnight under an argon atmosphere. The solvent was evaporated. The remaining solid was re-dissolved in a minimum amount of $CH_3OH$, and the desired compound was precipitated by dropwise addition of a saturated aqueous solution of ammonium hexafluorophosphate. The precipitate was filtered, washed with water then $Et_2O$ and dried under vacuum to yield 97 mg (0.09 mmol, 84%) of the desired hexafluorophosphate ruthenium complex (compound **12**) as a brown solid. $^1H$ NMR ($CD_3CN$): $\delta$ = 9.25 (br s, 1H, NH), 8.64 (br s, 1H), 8.49 (d, 4H, $J$ = 8.2 Hz, Ar-H), 8.35 (d, 1H, $J$ = 7.8 Hz, Ar-H), 8.04 (m, 4H, Ar-H), 7.80 (d, 1H, $J$ = 5.6 Hz, Ar-H), 7.72 (m, 4H, Ar-H), 7.53 (s, 2H),7.38 (m, 5H, Ar-H), 6.37 (br s, 3H, $NH_3$), 3.27 (t, 2H, $J$ = 6.2 Hz, $CH_2$), 2.83 (t, 2H, $J$ = 6.2 Hz, $CH_2$). $^{13}C$ NMR ($CD_3CN$): $\delta$ = 172.0, 158.5, 158.0, 157.8, 153.0, 152.8, 152.7, 152.7, 149.3, 147.5, 145.8, 145.6, 145.2, 144.7, 138.9, 138.7 (2C), 131.1, 131.0, 130.7, 130.4, 128.7, 128.5 (2C), 127.4, 126.9, 125.2 (2C), 125.1, 117.6, 114.3, 37.1, 33.3 ppm. MS m/z: calculated for $C_{33}H_{31}F_6N_8OPRu$ [$M+PF_6$]$^+$ 801.7 found ESI$^+$ 801.1.

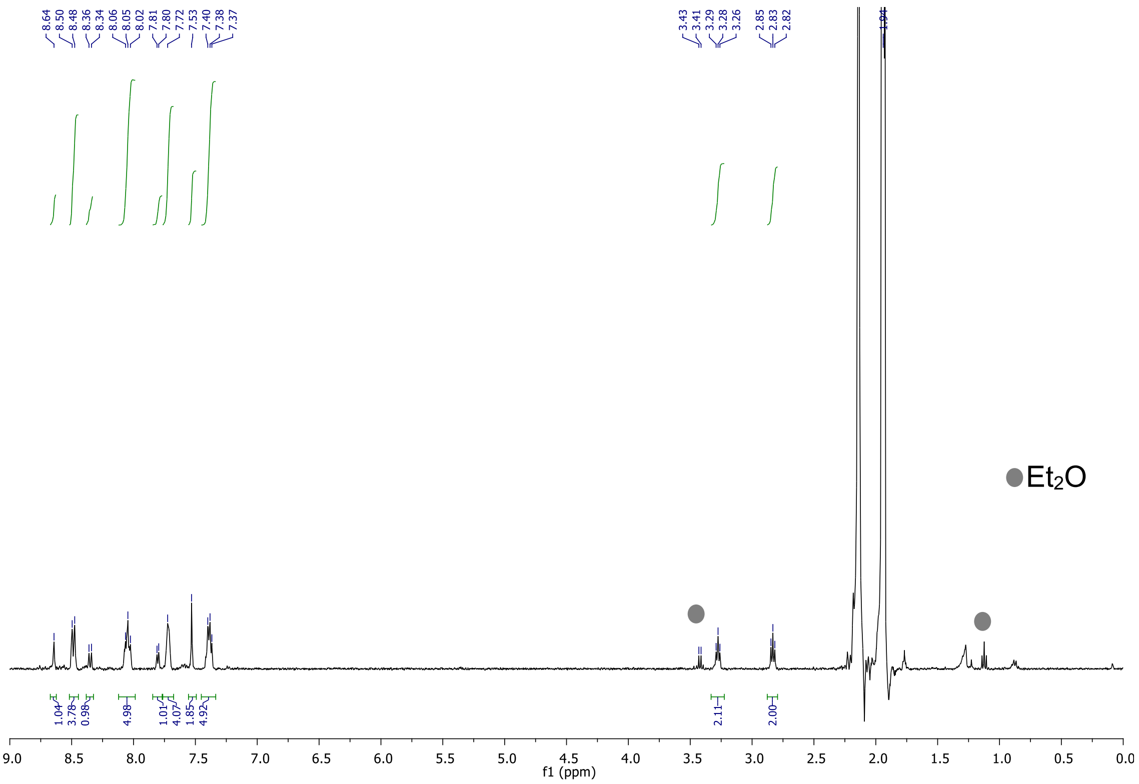
8.64
8.50
8.48
8.36
8.34
8.06
8.05
8.02
7.81
7.80
7.72
7.53
7.40
7.38
7.37
3.43
3.41
3.29
3.28
3.26
2.85
2.83
2.82
Et2O
1.04
3.78
0.98
4.98
1.01
4.07
1.85
4.92
2.11
2.00
f1 (ppm)

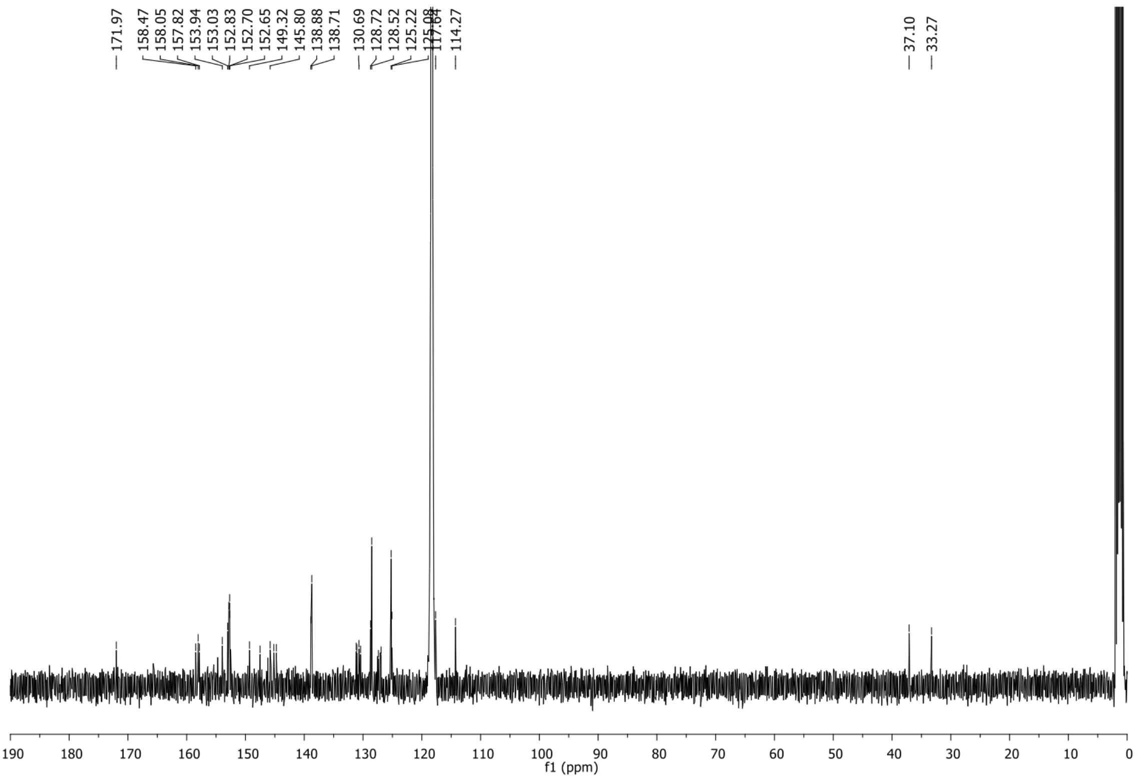
171.97
158.47
158.05
157.82
153.94
153.03
152.83
152.70
152.65
149.32
145.80
138.88
138.71
130.69
128.72
128.52
125.22
125.08
114.27
37.10
33.27
f1 (ppm)

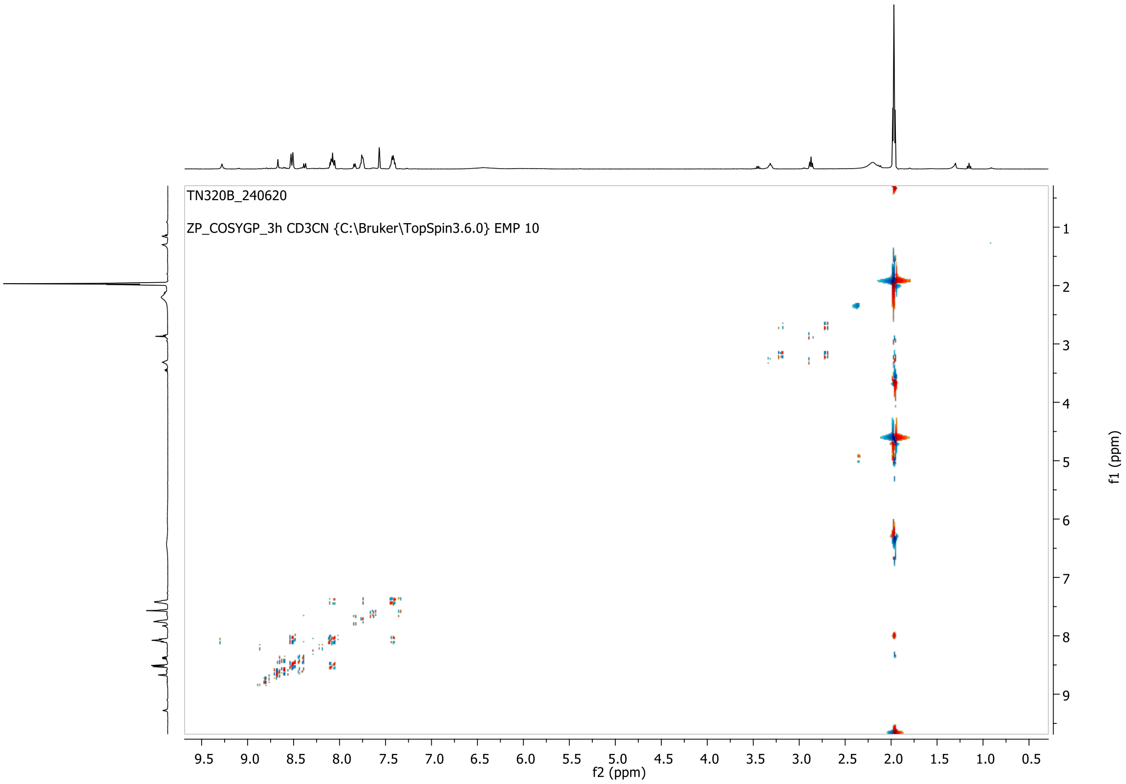
TN320B_240620
ZP_COSYGP_3h CD3CN {C:\Bruker\TopSpin3.6.0} EMP 10
f2 (ppm)
f1 (ppm)

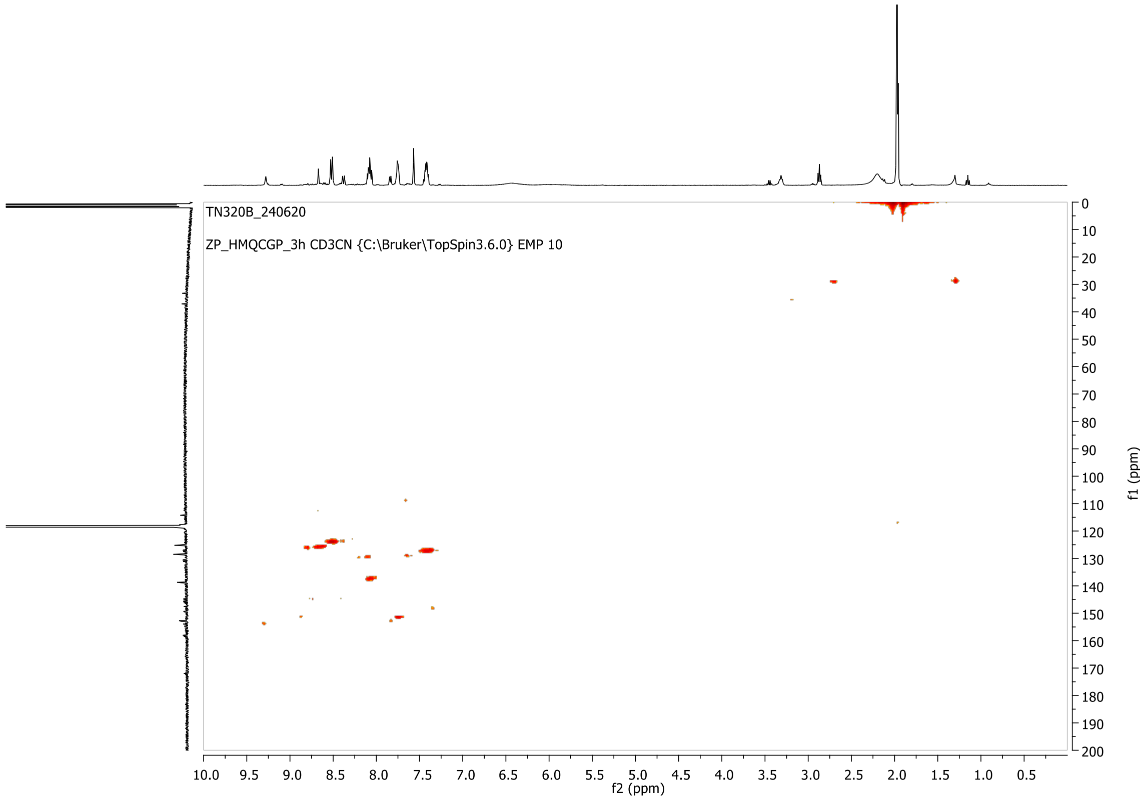
TN320B_240620
ZP_HMQCGP_3h CD3CN {C:\Bruker\TopSpin3.6.0} EMP 10
f2 (ppm)
f1 (ppm)

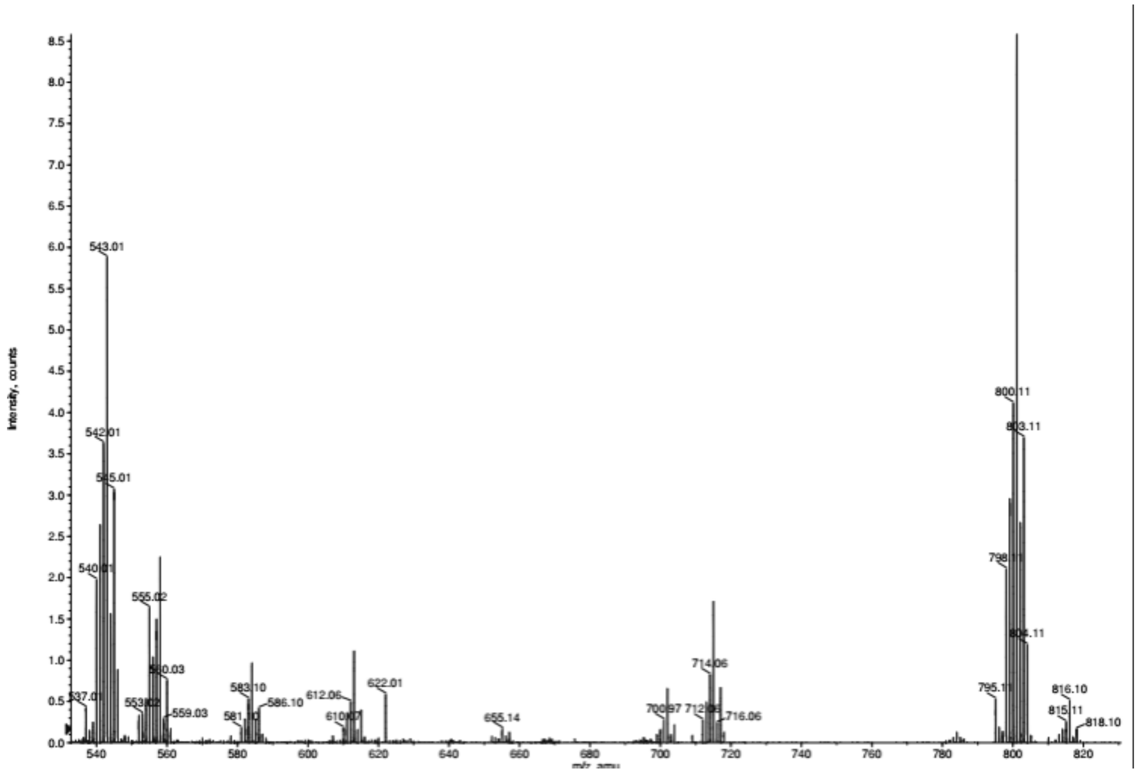


## 4. Synthesis HR-barb couple for AFM studies

### 4.a. HR synthesis

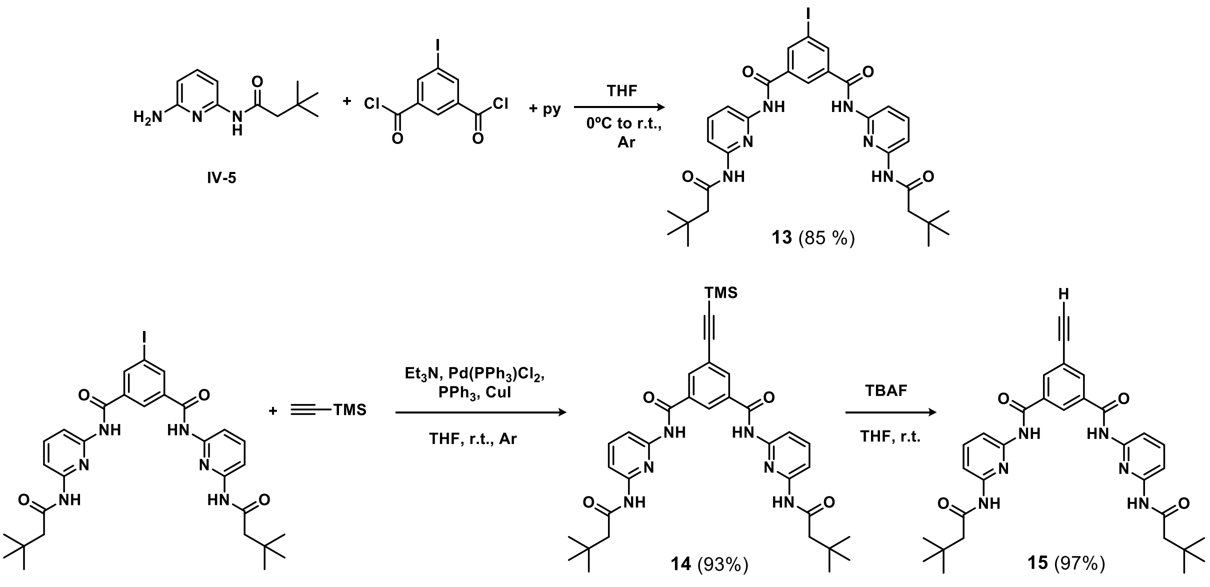


### 5-Iodo-*N,N'*-Bis[6-(3,3-dimethylbutyrylamino)pyridine-2-yl]isophthalamide

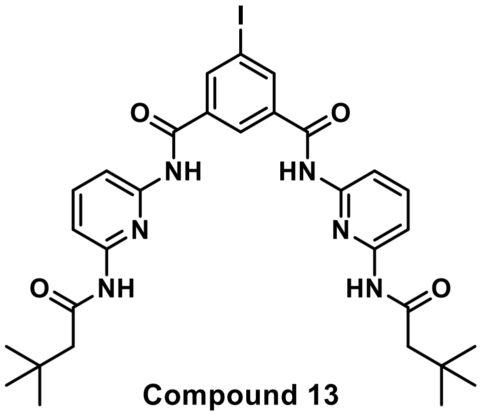

**Compound 13**

i) 5-iodoisophthalic acid (400mg 1.4 mmol) was suspended in anhydrous DCM (4.4 mL), 2 drops of DMF were added and oxalyl chloride (0.3 mL, 3.6 mmol) was added dropwise over a period of 15 min. The reaction was stirred for two hours, then the solvent was removed under reduced pressure and the crude material was directly used in the next step reaction without further purification.

ii) To the mixture of *N*-(6-aminopyridin-2-yl)-3,3-dimethylbutyramide **5** (500 mg, 2.6 mmol) and triethylamine (0.4 mL, 2.6 mmol) in THF (8 mL) at 0°C, 5-iodo-isophthaloyldichloride 18 (428 mg, 1.3 mmol) in THF (8 mL) was added over 1h. The mixture was stirred at 0°C for 3h and at room temperature for overnight. The mixture was filtered, the solid residue was washed with THF. After removal of the solvent under vacuum, the crude material was purified by column chromatography using dichloromethane and ethyl acetate (3/1) as eluent, to give compound **13** as a white powder (740 mg, 85%). $^1$H NMR ($CDCl_3$): $\delta$ = 8.62 (br. s., 1H; NH), 7.95 (m. 6H), 7.71 (t. *J* = 8.1 Hz. 3H), 2.26 (s. 4H; **$CH_2$**$C(CH_3)_3$), 1.10 (s. 18H; $CH_2C$**$(CH_3)_3$**). This data is in concordance with *Organometallics* **2014,** *33* (3), 665-676.[6]

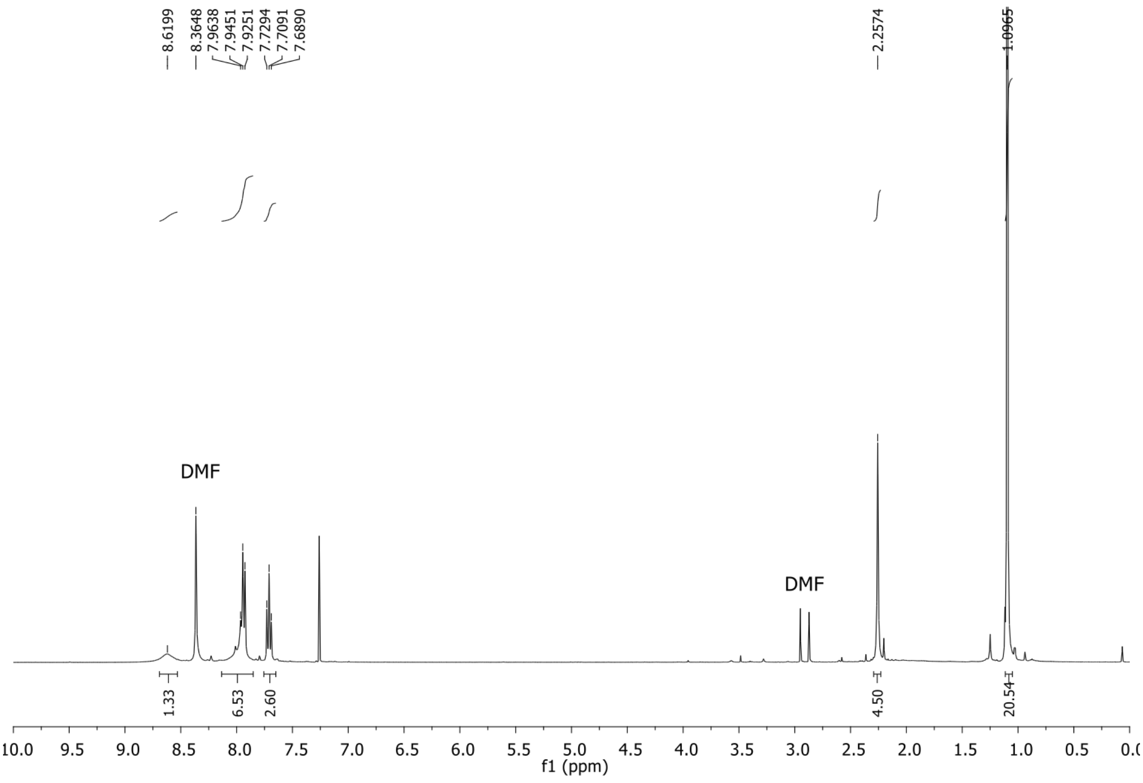


***N,N'*-Bis[6-(3,3-dimethylbutyrylamino)pyridine-2-yl]-trimethylsilyl-ethynyl-isophtalamide**

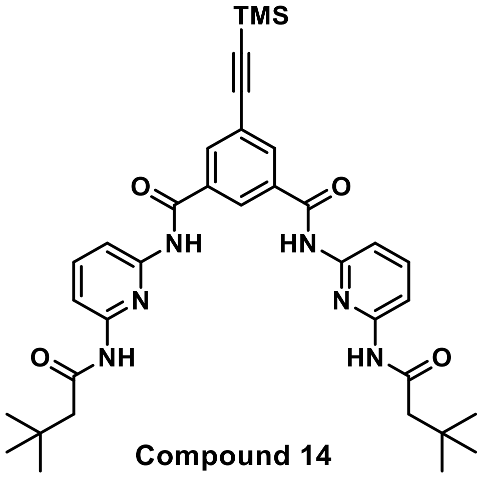


**Compound 14**

Compound **13** (200 mg, 0.30 mmol) was dissolved in anhydrous THF (5 mL) and distilled $NEt_3$ (2.2 mL). Then $PdCl_2(PPh_3)_2$ (2.1 mg, 0.0032 mmol), CuI (cat. amount) and $PPh_3$ (0.4 mg, 0.001 mmol) were added. After stirring the reaction mixture for 15 min to dissolve the catalysts, trimethylsilylacetylene (36 mg, 0.36 mmol) was added slowly and dropwise to the suspension. The reaction mixture was then stirred at room temperature for overnight. The solvent was removed under reduced pressure, and the crude material was purified by column chromatography using DCM/EtOAc 8/1-7/1 mixture as a eluent, to furnish compound **14** as a yellow solid 179 mg, 93%. $^1$H NMR ($CDCl_3$): $\delta$ = 8.41 (br s,

2H, NH), 8.37 (br s, 1H, NH), 8.13 (s, 2H), 7.99 (t, *J* = 4.9 Hz, 4H), 7.74 (t, *J* = 8.0 Hz, 2H), 2.27 (s, 4H, **$CH_2$**$C(CH_3)_3$), 1.11 (s, 18H, $CH_2C$**$(CH_3)_3$**), -0.28 (s, 9H, $Si(CH_3)_3$). $^{13}C$ NMR ($CDCl_3$): $\delta$ = 170.4 (2C), 163.5 (2C), 149.7 (2C), 149.0 (2C), 140.9 (2C), 134.9, 133.7, 125.5, 125.2, 110.2 (2C), 109.7 (2C), 102.3, 98.0, 51.7 (2C), 31.4 (2C), 29.8 (6C), -0.3 (3C) ppm. This data is in concordance with *Organometallics* **2014,** *33* (3), 665-676.

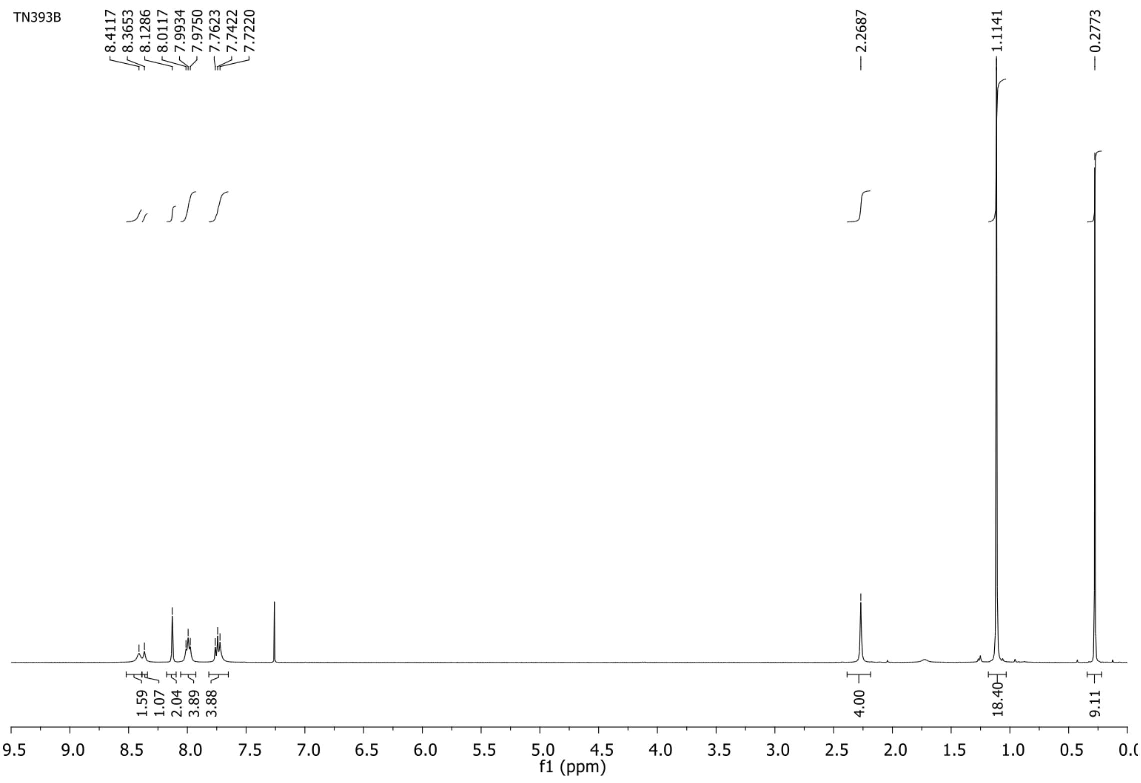

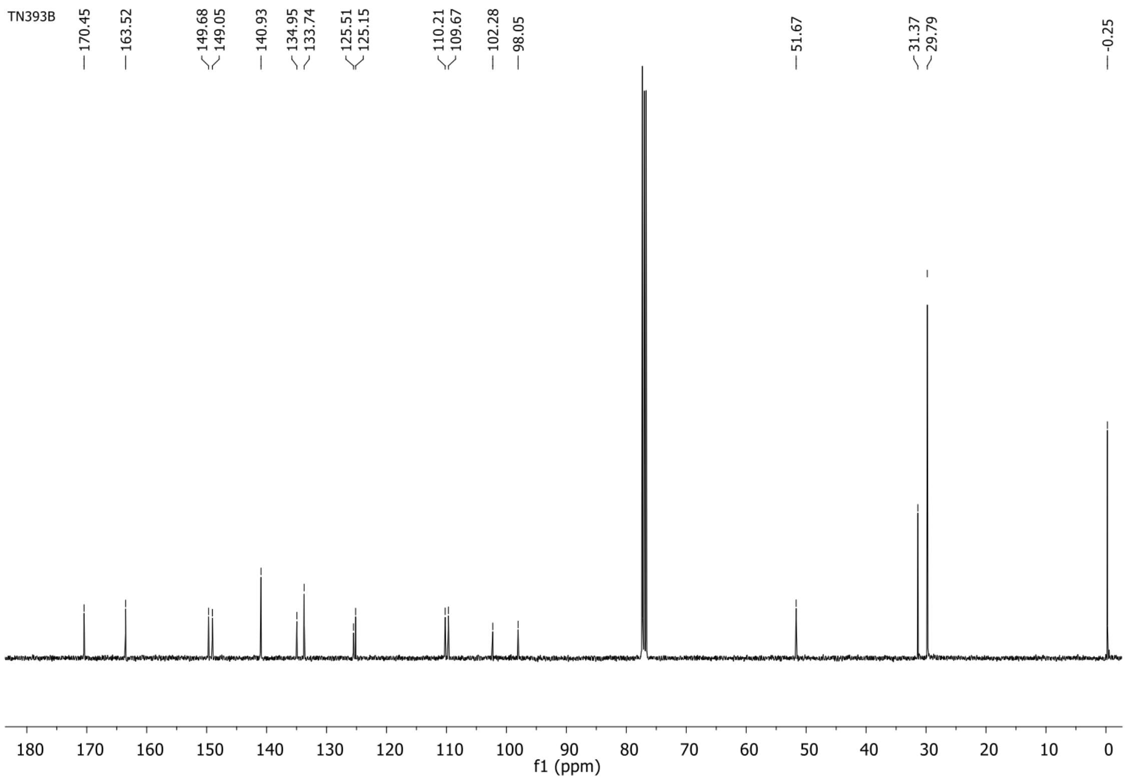


**5-Ethynyl-*N,N'*-Bis[6-(3,3-dimethylbutyrylamino)pyridine-2-yl] isophtalamide**

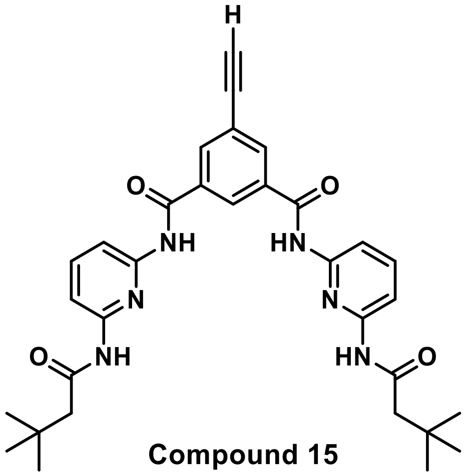


**Compound 15**

Tetrabutylammonium fluoride solution (TBAF) (74 μL, 0.26 mmol) in THF was added dropwise to a solution of compound **14** (140 mg, 0.23 mmol) in dry THF (3mL) at room temperature. After stirring for 3h (TLC), solvent was evaporated and the residue purified by column chromatography, using DCM/ AcOEt 4/ 1 as eluent, to furnish compound **15**, 143 mg (97%) as a brown powder. $^1$H NMR ($CDCl_3$): $\delta$ = 8.48 (br s, 2H, NH), 8.44 (br s, 1H, NH), 8.07 (s, 1H), 8.00 (s, 2H), 7.98 (d, $J$ = 8.1 Hz, 2H), 7.91 (d, $J$ = 8.0 Hz, 2H), 7.68 (2H $J$ = 8.1 Hz, 2H), 3.19 (s, 1H, ≡CH), 2.26 (s, 4H, **$CH_2$**$C(CH_3)_3$), 1.13 (s, 18H, $CH_2C$**$(CH_3)_3$**). $^{13}$C NMR ($CDCl_3$): $\delta$ = 170.7 (2C), 163.6 (2C), 149.8 (2C), 148.9 (2C),

140.9 (2C), 134.9 (2C), 134.1 (2C), 125.9, 124.0, 110.4 (2C), 109.7 (2C), 81.2, 80.3, 51.4 (2C), 31.4 (2C), 29.8 (6C). This data is in concordance with *Organometallics* **2014,** *33* (3), 665-676.

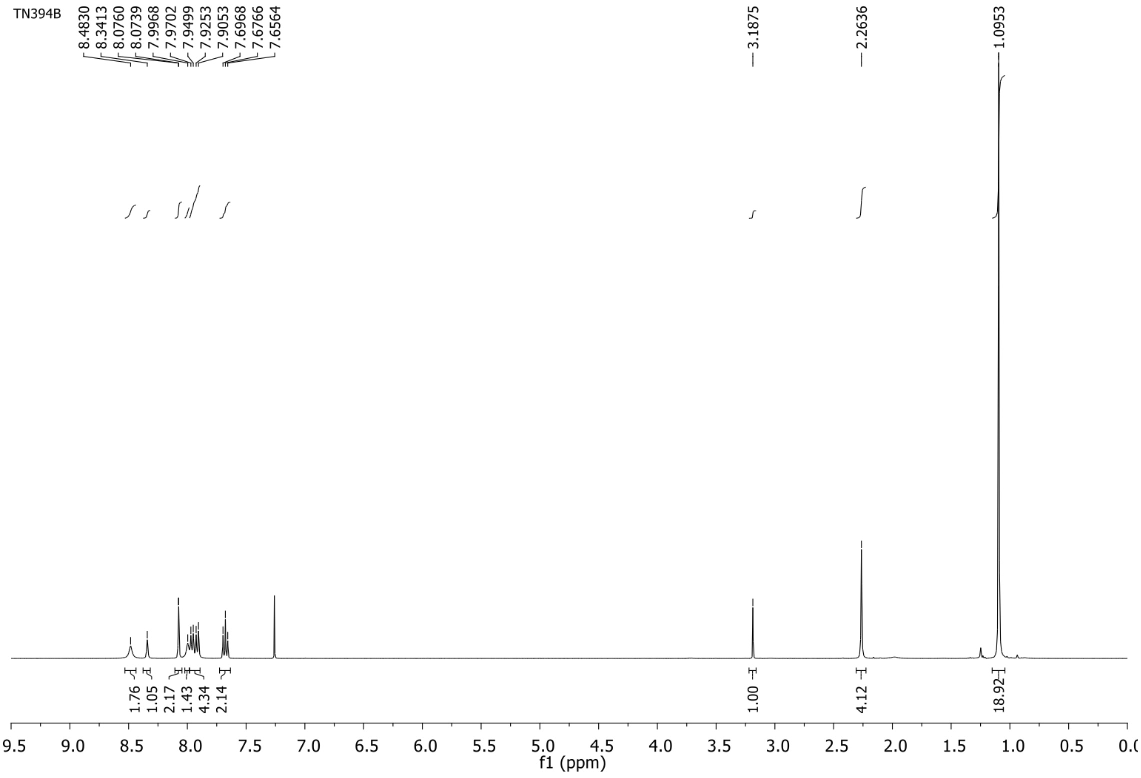


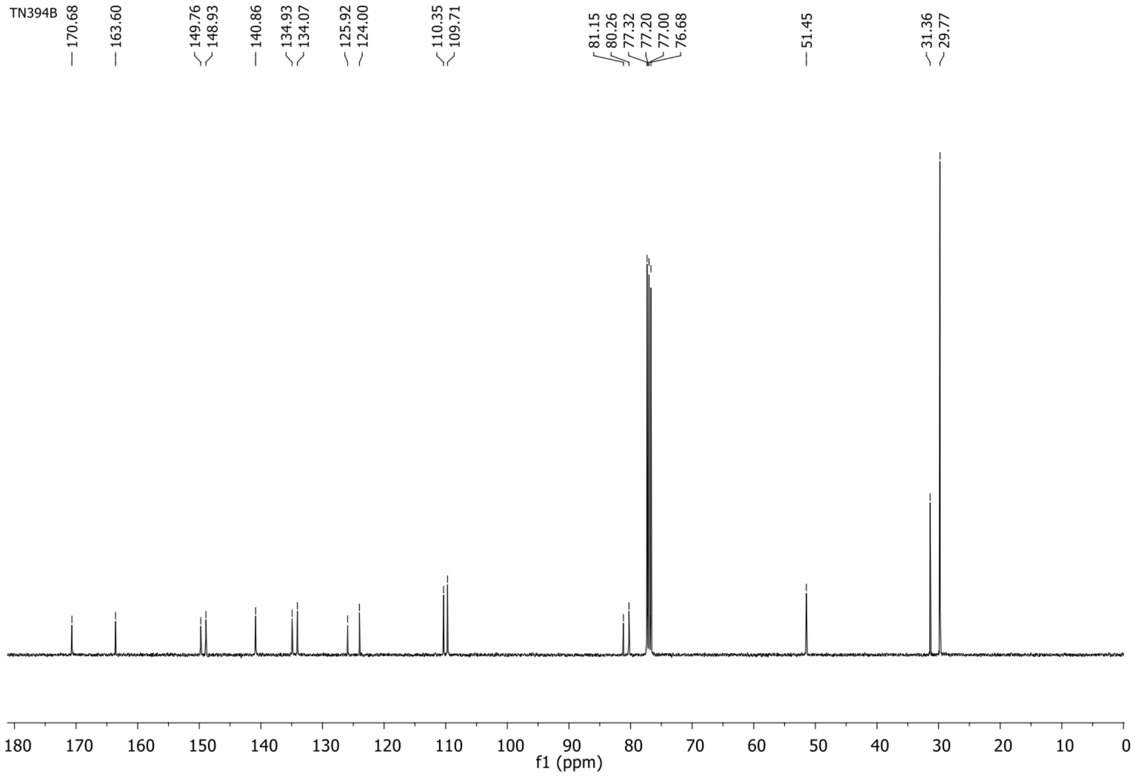


## 4.b. Barbituric derivative synthesis

Barbituric derivative (compound **18**) was synthetized following the procedure describe by Huang, C.-H. et al. [7]

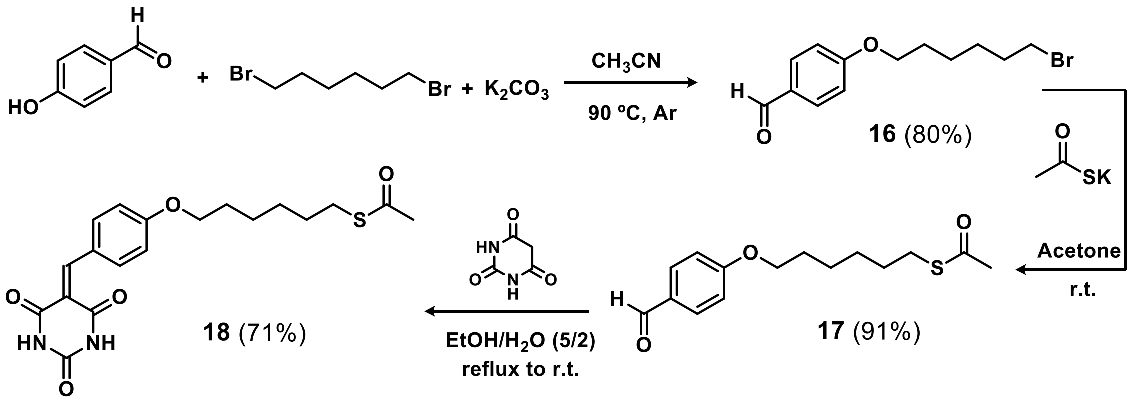


**4-((6-bromohexyl)oxy)benzaldehyde**

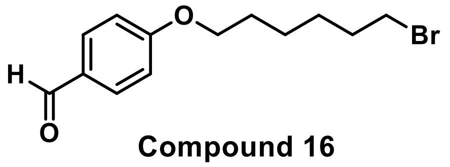

**Compound 16**

1,6-Dibromohexane (9 g, 36.8 mmol) and $K_2CO_3$ (6.8 g, 49.2 mmol) were added to a solution of 4-hydroxybenzaldehyde (3 g, 24.6 mmol) in $CH_3CN$ (17 mL). The mixture was refluxed overnight, the white precipitate was filtered off and the filtrated was extracted with $CH_3Cl$, washed with brine and dried over $Na_2SO_4$. The crude material was purified by column chromatography, using DCM/ MeOH 200/1-60/1 as eluent; to furnish 5.6 g (80%) as white solid. $^1H$ NMR ($CDCl_3$): $\delta$ = 9.87 (s, 1H, CHO), 7.81 (d, $J$ = 8.6 Hz, 2H, Ar-H), 6.97 (d, $J$ = 8.7 Hz, 2H, Ar-H), 4.04 (t, $J$ = 6.4 Hz, 2H, $OCH_2$), 3.42 (t, $J$ = 6.7 Hz, 2H, $BrCH_2$), 1.89 (m, 2H, $CH_2$), 1.83 (m, 2H, $CH_2$), 1.51 (m, 4H, $CH_2$). $^{13}C$ NMR ($CDCl_3$): $\delta$ = 190.8, 164.1, 132.0 (2C), 129.8, 114.7 (2C), 68.1, 33.7, 32.6, 28.9, 27.8, 25.2 ppm.

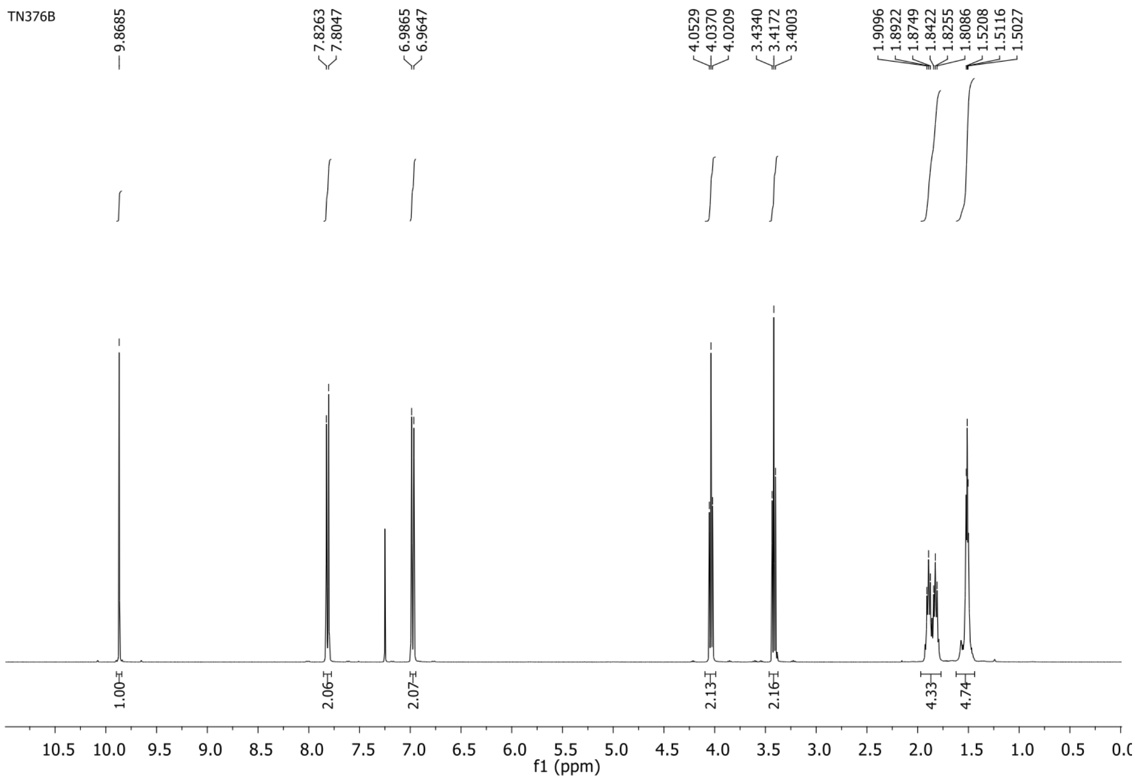
TN376B
9.8685
7.8263
7.8047
6.9865
6.9647
4.0529
4.0370
4.0209
3.4340
3.4172
3.4003
1.9096
1.8922
1.8749
1.8422
1.8255
1.8086
1.5208
1.5116
1.5027
1.00
2.06
2.07
2.13
2.16
4.33
4.74
10.5 10.0 9.5 9.0 8.5 8.0 7.5 7.0 6.5 6.0 5.5 5.0 4.5 4.0 3.5 3.0 2.5 2.0 1.5 1.0 0.5 0.0
f1 (ppm)

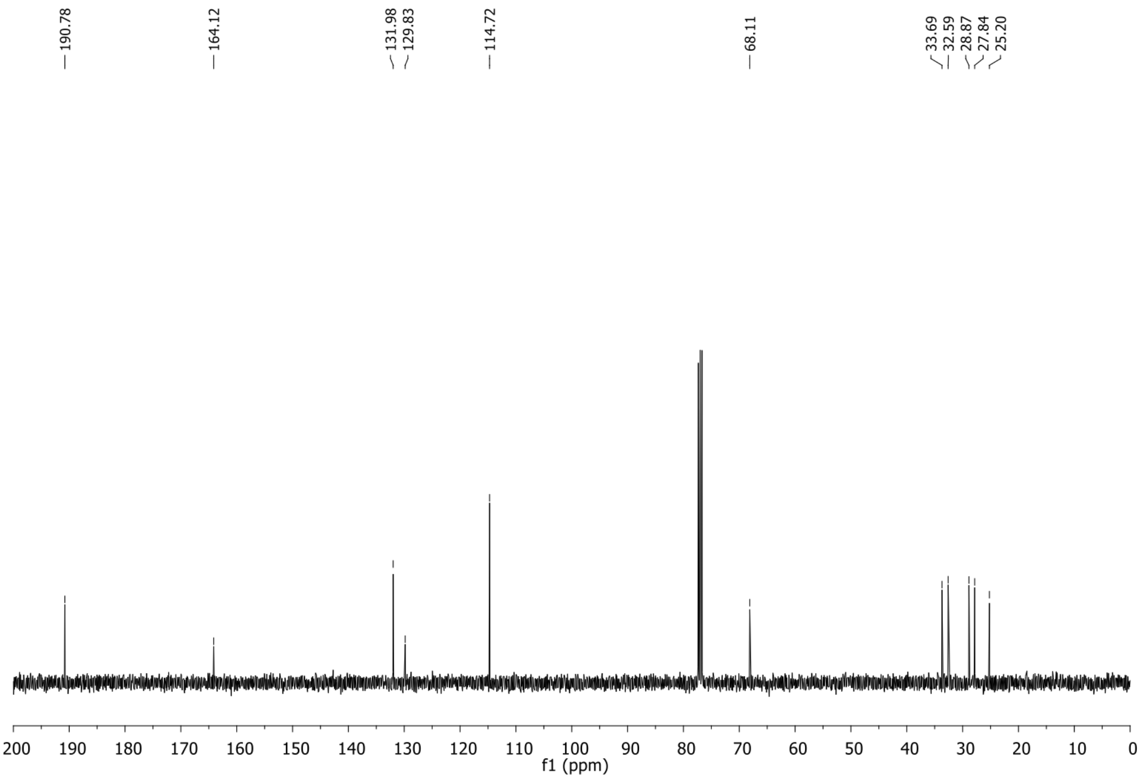
190.78
164.12
131.98
129.83
114.72
68.11
33.69
32.59
28.87
27.84
25.20
200 190 180 170 160 150 140 130 120 110 100 90 80 70 60 50 40 30 20 10 0
f1 (ppm)

## 6-(4-formylphenoxy)hexyl thioacetate

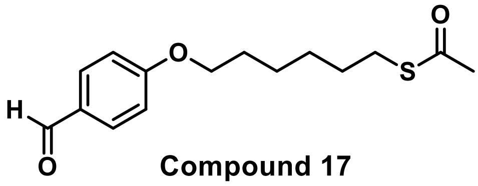

**Compound 17**

4-(6-Bromohexyloxy)-benzaldehyde (3.44 g, 12.1 mmol) and potassium thioacetate (1.38 g, 12.1 mmol) in acetone (65 mL) were stirred at room temperature overnight. The resulting white precipitate was filtered off and the filtrate was concentrated under reduced pressure to give a white solid (3.08 g, 91%). $^1$H NMR ($CDCl_3$): $\delta$ = 9.88 (s, 1H, CHO), 7.82 (d, 2H, *J* = 8.7 Hz, Ar-H), 6.98 (d, 2H, *J* = 8.7 Hz, Ar-H), 4.04 (t, 2H, *J* = 6.3 Hz, $OCH_2$), 2.88 (t, 2H, J = 7.1 Hz, $SCH_2$), 2.32 (s, 3H, $CH_3$), 1.82 (m, 2H), 1.59 (m, 2H), 1.48 (m, 4H).

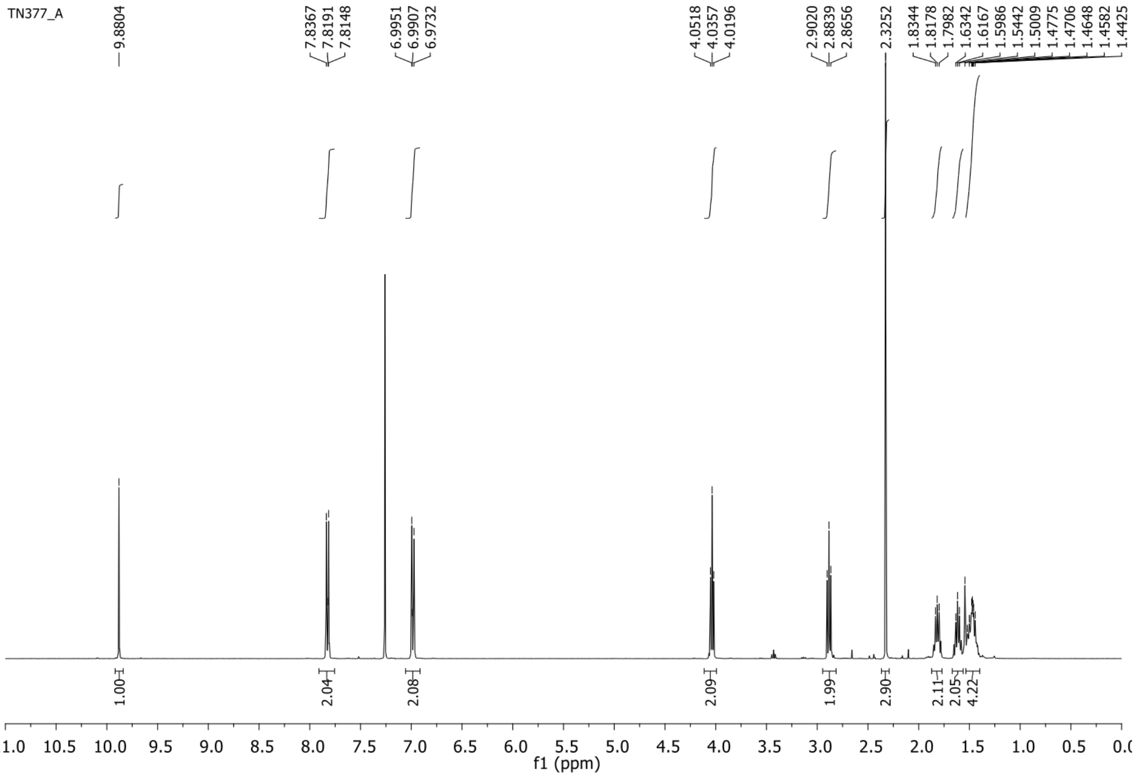


## 6-[4-((1,3-Dimethyl-tetrahydro-2,4,6-trioxo-5(2H)-pyrimidinyliene)methyl) phenoxy] hexyl thioacetate

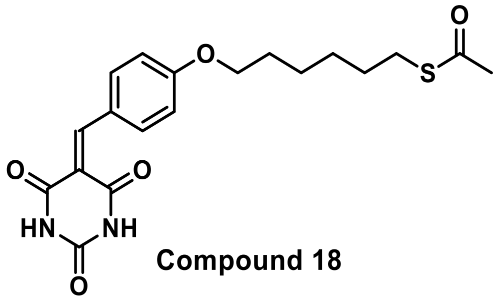

**Compound 18**

Compound **17** (1.6 g, 5.71 mmol), and barbituric acid (0.73 g, 5.71 mmol) in EtOH/ $H_2O$ 5/2 (35 mL) were stirred under reflux for 10 min and then at room temperature overnight. The resulting yellow suspension was filtered, washed with EtOH and dried under reduced pressure to give a yellow solid (1.58 g, 71%). $^1H$ NMR ([D6]dimethyl sulfoxide): $\delta$ = 11.28 (s, 1H, NH-barb), 11.15 (s, 1H, NH-barb), 8.35 (d, 2H, *J* = 9.1 Hz, Ar-H), 7.04 (d, 2H, *J* = 9.0 Hz, Ar-H), 4.09 (t, 2H, *J* = 6.5 Hz, $OCH_2$), 2.84 (t, 2H, *J* = 7.2 Hz, $SCH_2$), 2.31 (s, 3H, $CH_3$), 1.73 (m, 2H), 1.53 (m, 2H), 1.40 (m, 4H); $^{13}C$ NMR ([D6]dimethyl sulfoxide): $\delta$ = 195.3, 163.9, 162.9, 162.2, 154.9, 150.2, 142.2, 137.5 (2C), 125.0, 115.4, 114.3 (2C), 67.9, 30.6, 29.0, 28.4, 27.8, 24.9 ppm.

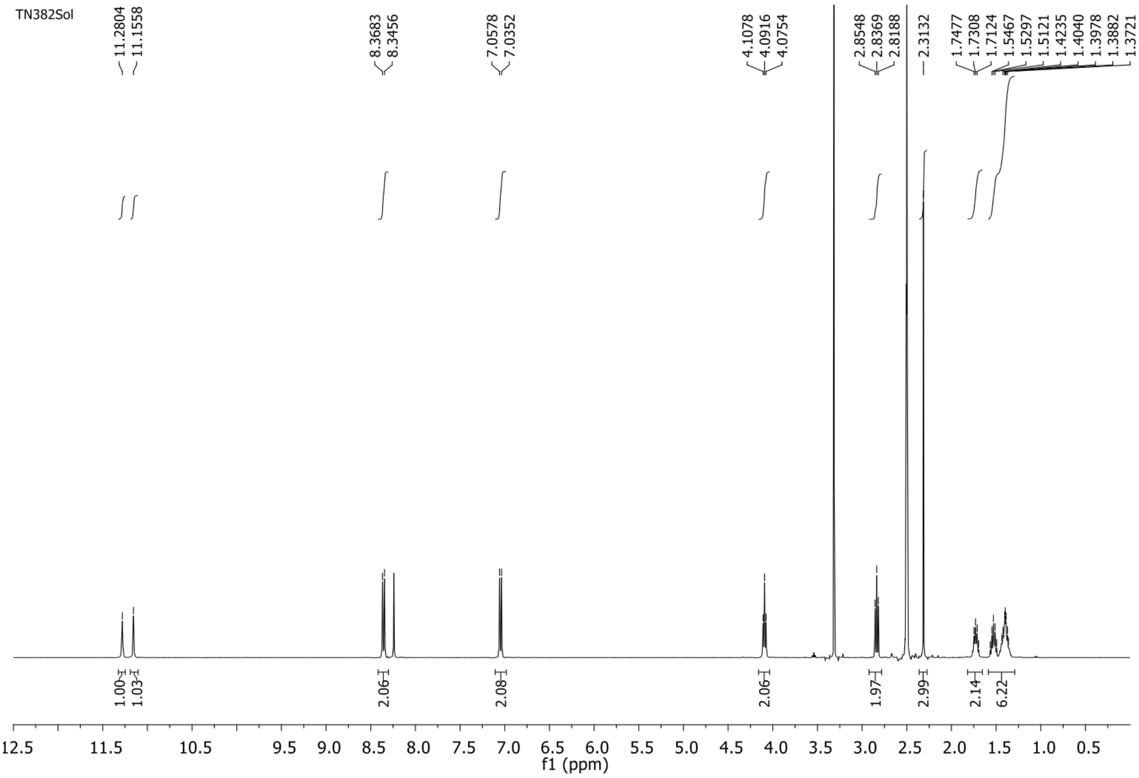

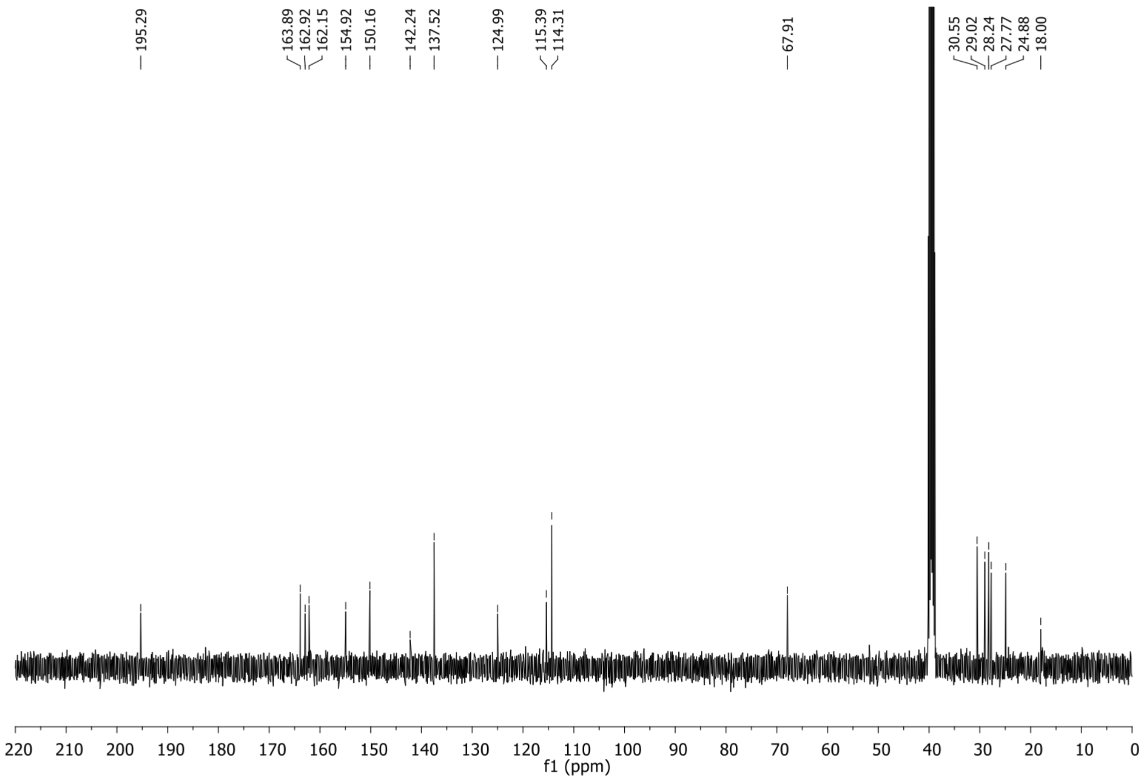


### 4.c. Synthesis of 11-azidoundecane-1-thiol

11-azidoundecane-1-thiol (compound **22**) was synthetized following the procedure described by Liu, X. et al. [8]

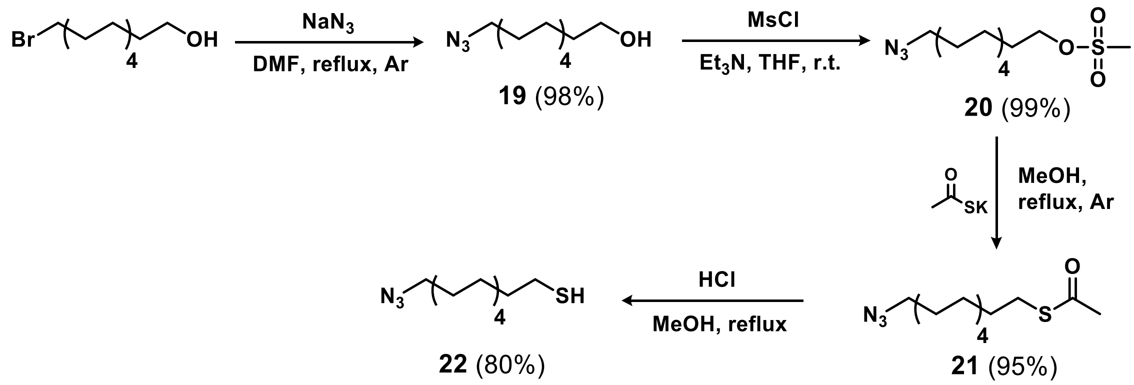


### 11-azidoundecan-1-ol

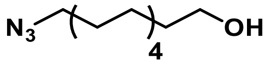


**Compound 19**

Sodium azide (1.43 g, 22.0 mmol) was added to a solution of 11-bromoundecan-1-ol (5 g, 19.9 mmol) in 75 mL DMF. The reaction mixture was stirred under reflux and argon

atmosphere overnight. The reaction mixture was allowed to cool to room temperature, followed by the addition of 75 mL $H_2O$. The resulting mixture was extracted with diethyl ether. Then the organic phase was combined, dried with $MgSO_4$, and concentrated under reduced pressure to give compound as a colorless oil (4.25 g, 98%). $^1$H NMR ($CDCl_3$): $\delta$ = 3.64 (t, 2H, *J* = 6.5 Hz, $OCH_2$), 3.25 (t, 2H, *J* = 6.8 Hz , $N_3CH_2$), 1.56 (m, 4H, $CH_2$), 1.31 (m, 14H, $CH_2$).

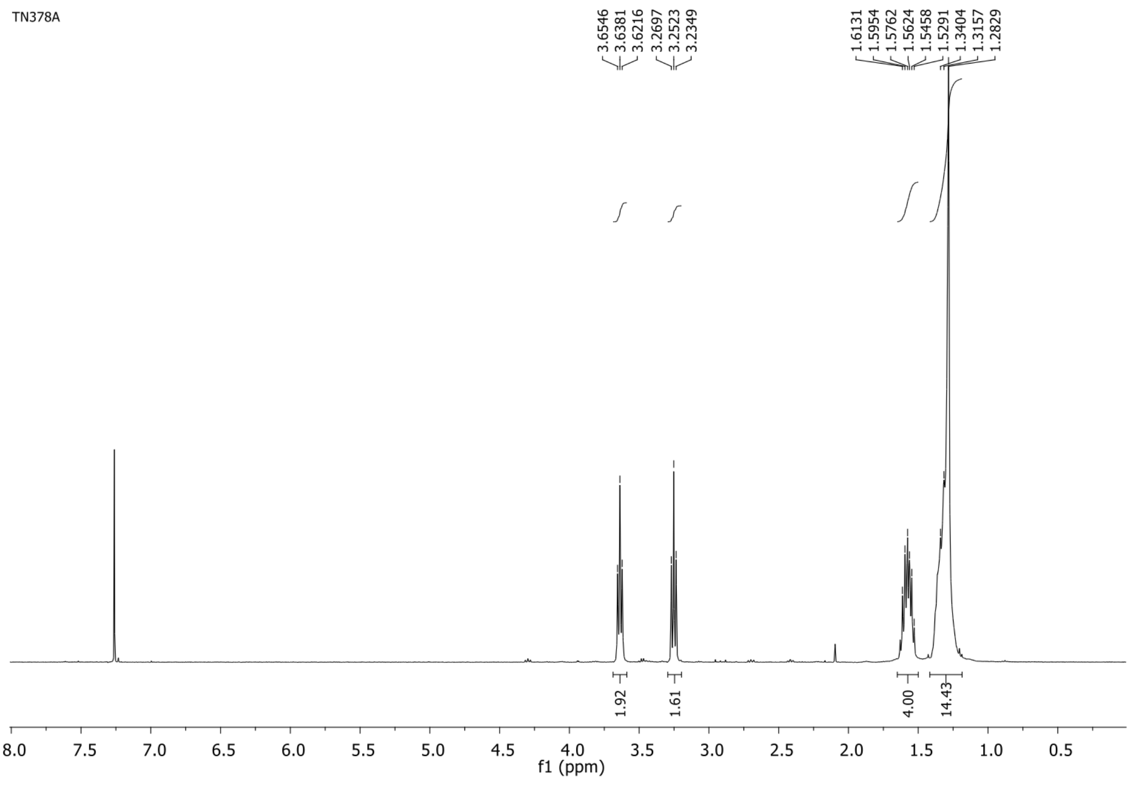


**5-azidoundecyl methanesulfonate**

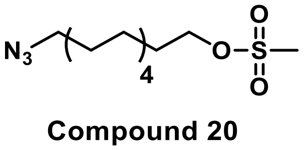


**Compound 20**

To a solution of compound **19** (1.5 g, 6.87 mmol) and $NEt_3$ (1.9 mL, 13.70 mmol) in THF (52 mL) was added methanesulfonyl chloride (1.5 mL, 19.30 mmol) dropwise in an ice bath. The reaction was stirred at 0 ºC for 30 min and at room temperature for another 90 min under argon. Then 52 mL of ice-cold water was added. The aqueous phase was extracted twice with diethyl ether. The combined organic phases were then combined and washed with 1M HCl, $H_2O$, $NaHCO_3$ and $H_2O$. After drying over MgSO4, the solvent was removed under reduced pressure to yield a colorless oil (2 g, quantitative). $^1$H NMR

(CDCl3): δ = 4.22 (t, 2H, *J* = 6.6 Hz, $CH_2$), 3.26 (t, 2H, *J* = 6.8 Hz, $CH_2$), 3.00 (s, 3H, $CH_3$), 1.75 (m, 2H, $CH_2$), 1.60 (m, 2H, $CH_2$ ), 1.36 (m, 14H, $CH_2$).

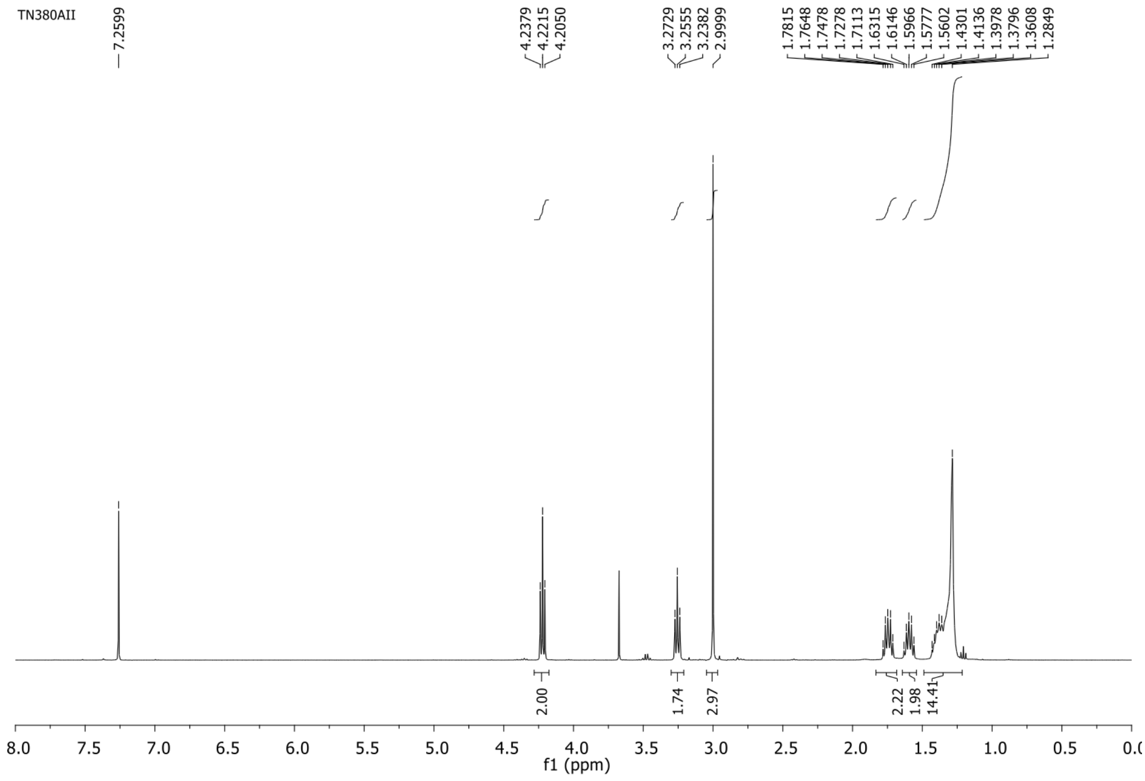


**11-azidoundecyl thioacetate**

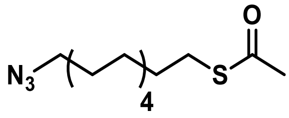


**Compound 21**

Compound **20** (1.7 g, 5.83 mmol) and potassium thioacetate (1.33 g, 11.7 mmol) was dissolved in methanol (50 mL). The reaction mixture was degassed and refluxed under argon for 3h. After colling to room temperature, excess methanol was removed under reduced pressure and ice water was added. The mixture was extracted with diethyl ether (×3) and washed with $H_2O$ (x3). Then the organic phase was combined, dried with $MgSO_4$ and concentrated to yield a pale yellow oil (1.61 g, 95%). $^1$H NMR ($CDCl_3$): δ = 3.24 (t, 2H, *J* = 6.88 Hz, $CH_2$), 2.86 (t, 2H, *J* = 6.88 Hz , $CH_2$), 2.31 (s, 3H, $CH_3$), 1.60 (m, 4H, $CH_2$), 1.33 (m, 14H, $CH_2$);

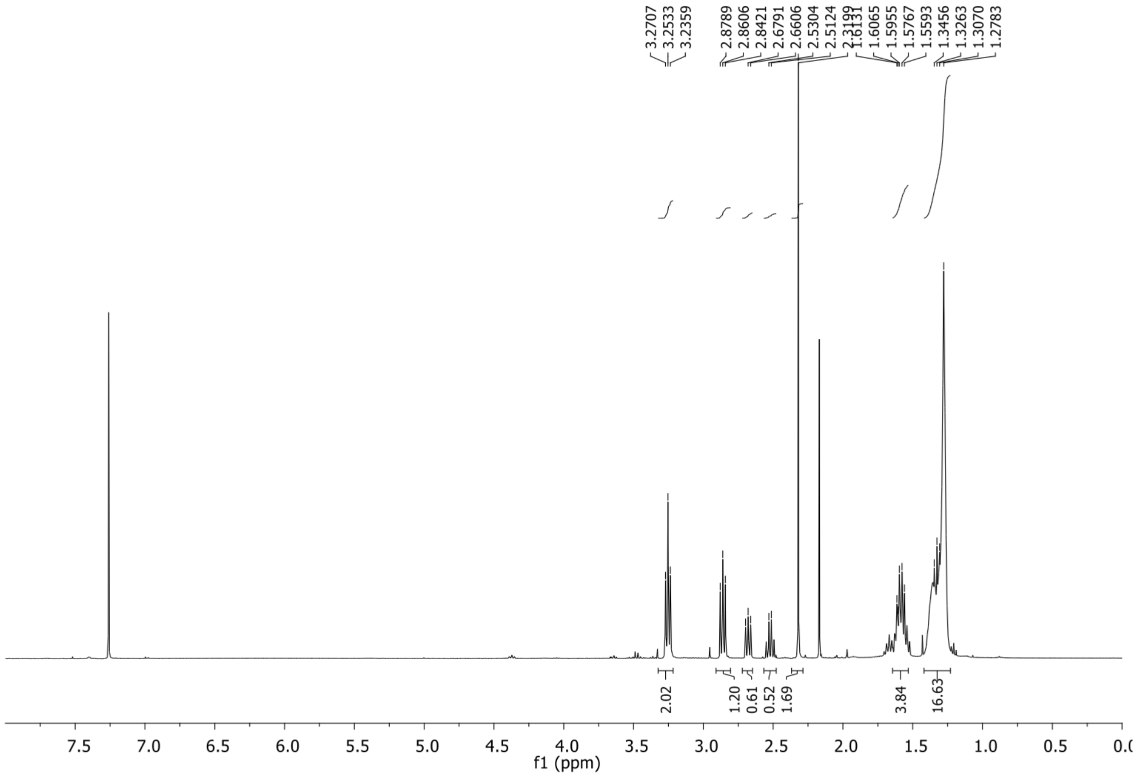


**11-azidoundecane-1-thiol**

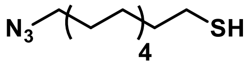


**Compound 22**

To 300 mg of compound **21**, 20mL of methanol was added. The solution was degassed thoroughly and backfilled with $N_2$. 1 mL conc. HCl was added, and the entire mixture was refluxed for 5h. The reaction mixture was quenched with water and extracted twice with diethyl ether. The organic phase was washed twice with water (x2) and dried over $MgSO_4$. Rotary evaporation yielded a yellow oil. 80% Yield. $^1$H NMR ($CDCl_3$): $\delta$ = 3.25 (t, 2H, $J$ = 7.00 Hz, $CH_2$), 2.68 (t, 0.6 H, $J$ = 7.25 Hz, $CH_2$ in disulfide), 2.52 (q, 2H, $J$ = 11.0 Hz, $CH_2$), 1.61 (m, 4H, $CH_2$), 1.24 (m, 14H, $CH_2$).

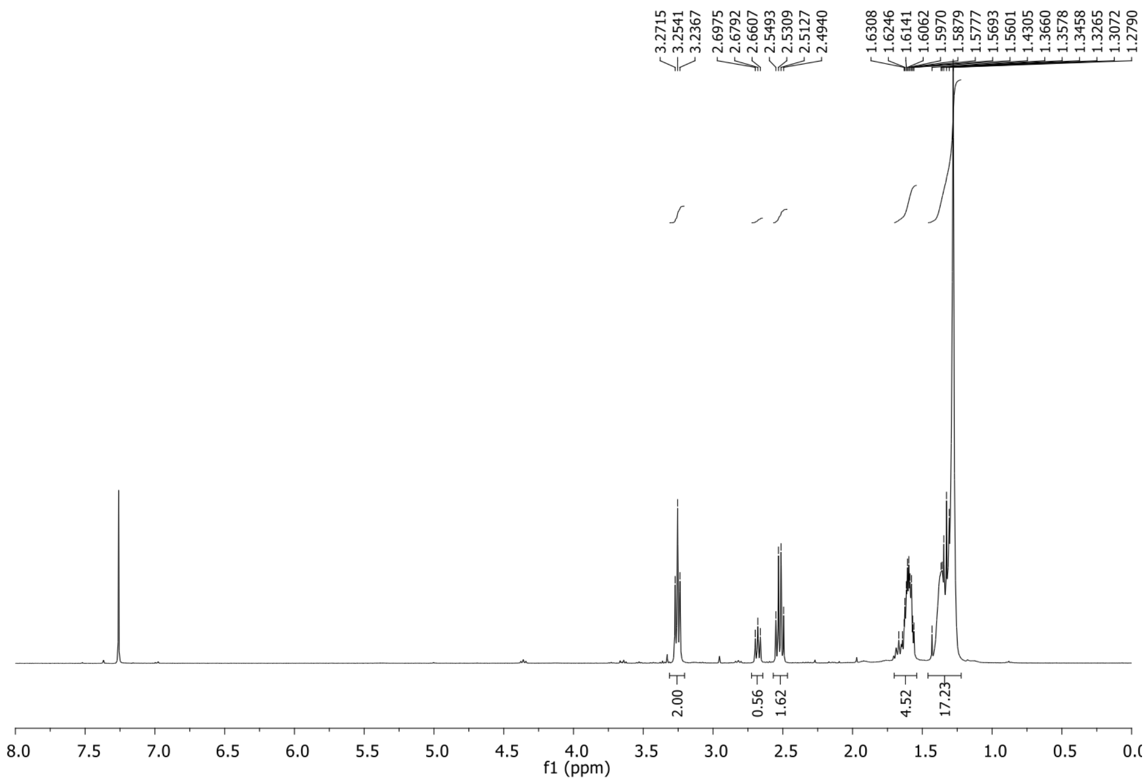


## 5. Description of UV/Vis titration experiments

UV/Vis experiments were performed on a Varian Cary® 50 UV/Vis spectrometer. Absolute concentrations for each component of a titration experiment were determined by standard calibration curves, and appropriate aliquots added relative to the determined concentrations. All titration were performed with a background concentration of host in the guest solution so as to maintain a constant concentration of host species. Aliquot injections were carried out with MICROMAN™ E positive displacement pipettes into a quartz cuvette (cuvette path length was dependent on concentrations used: 1.0 mm or 10.0 mm) with a Teflon stopper.

Each spectrum was acquired at a resolution of 1.0 nm and an integration time of 0.1 s. Spectral analyses were performed using the ReactLab Equilibria spectral analyses suite (JplusConsulting, www.jplusconsulting.com). Repetitions of the binding experiments for each complex gave association constants within 10% of the values shown, (the error in data fitting for each experiment was <5%).

## UV/Vis titrations experiments of HR vs Cy couple

### a) Acetonitrile

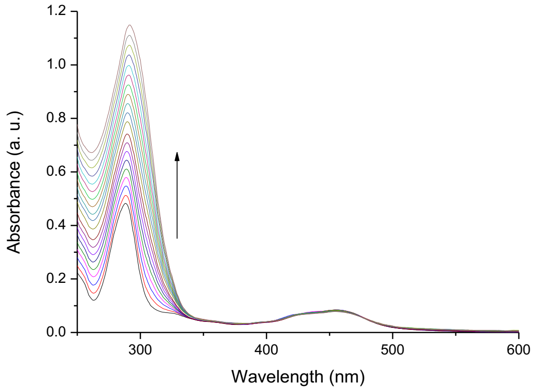


**Figure S2:** UV/Vis spectra of **4** (ca. 4 x $10^{-6}$ M) upon addition of **9** (0 → 2 equiv), while maintaining the concentration of **4** constant, in $CH_3CN$ at 298 K.

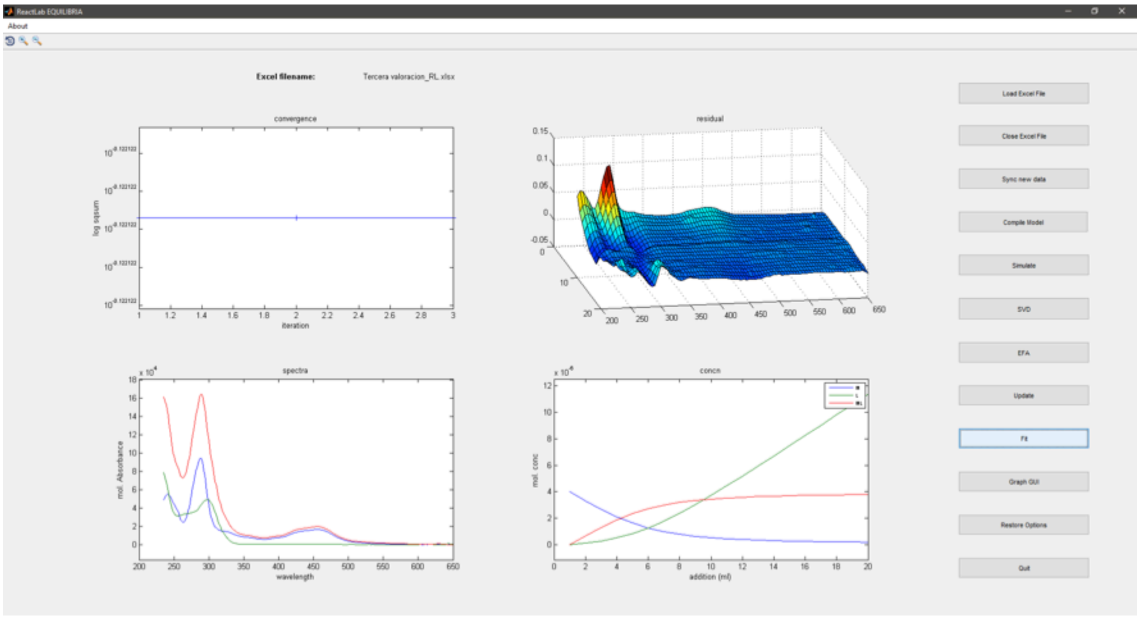


**Figure S3:** UV/Vis titration of **4** with **9** in $CH_3CN$, ReactLab Working Window (JplusConsulting, www.jplusconsulting.com). Top left, plot of least-squares optimization during a fit. Top right, a 3D plot of the residuals for the fit. Bottom left, the calculated spectrum for each species. Bottom right, concentration profile for the species in solution.

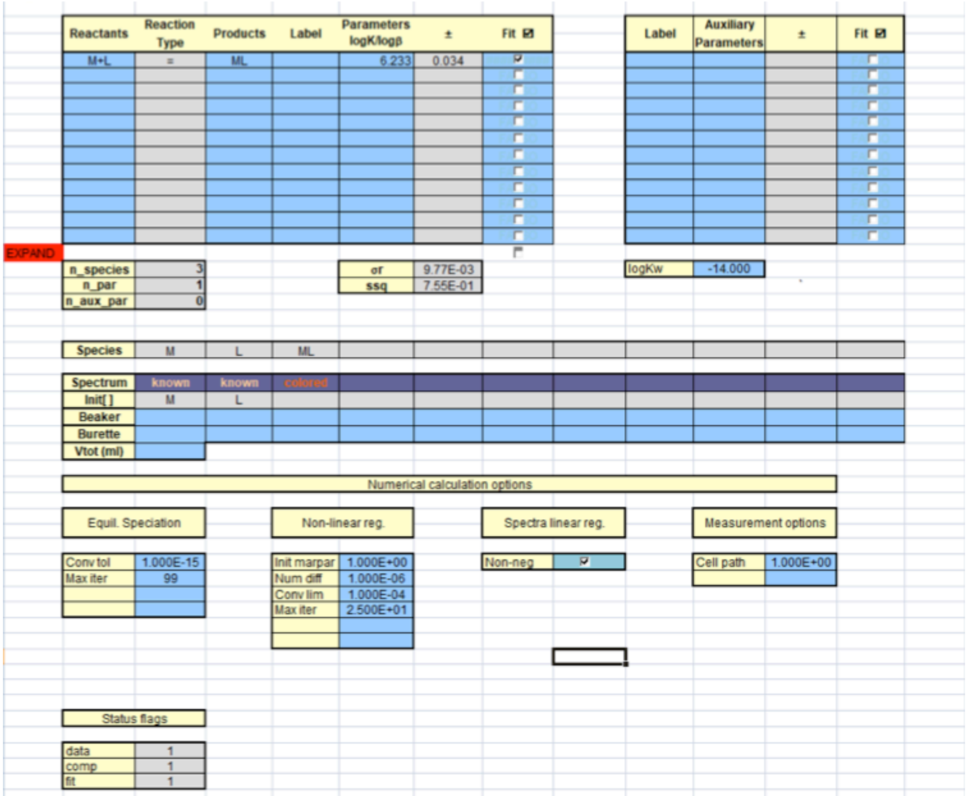


**Figure S4:** UV/Vis titration of **4** with **9** in $CH_3CN$, ReactLab Input/Output spreadsheet displaying log(*K*) =6.233.

## b) [EMIM][FAP]

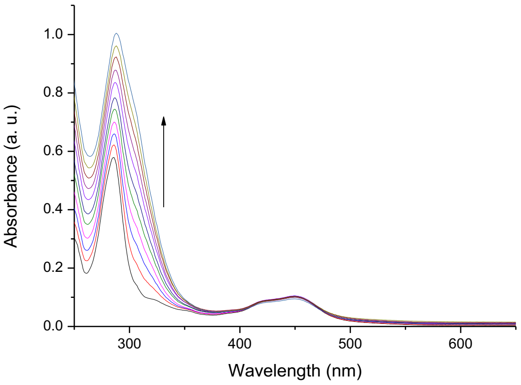


**Figure S5:** UV/Vis spectra of **4** (ca. 1.07 x $10^{-5}$ M) upon addition of **9** (0 → 2 equiv), while maintaining the concentration of **4** constant, in [EMIM][FAP] at 298 K.

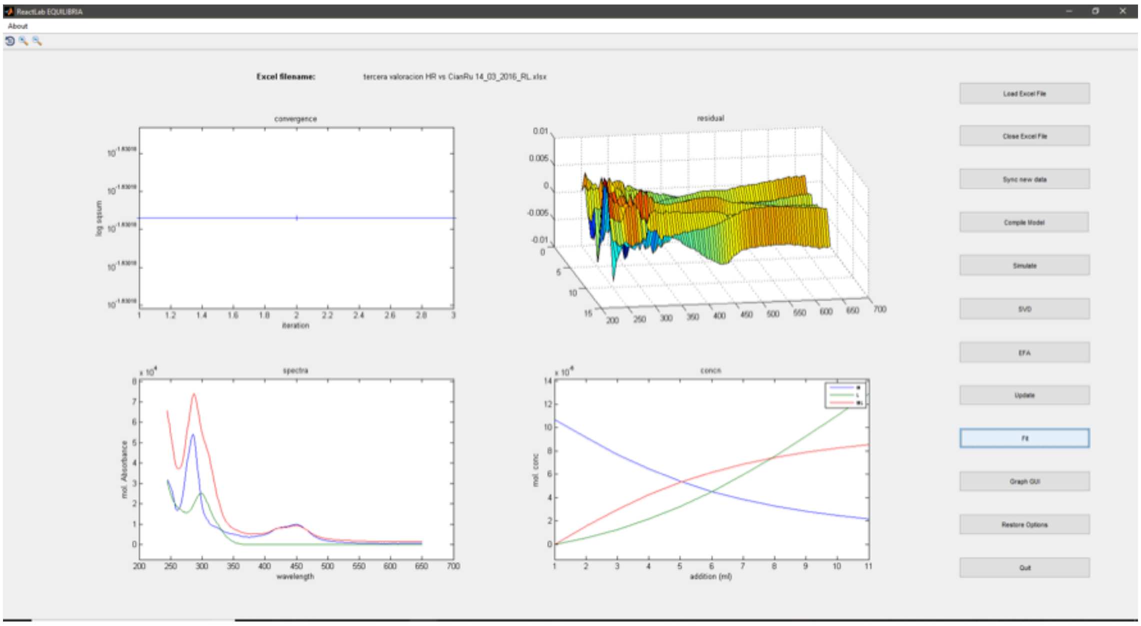


**Figure S6:** UV/Vis titration of **4** with **9** in [EMIM][FAP], ReactLab Working Window (JplusConsulting, www.jplusconsulting.com). Top left, plot of least-squares optimization during a fit. Top right, a 3D plot of the residuals for the fit. Bottom left, the calculated spectrum for each species. Bottom right, concentration profile for the species in solution.

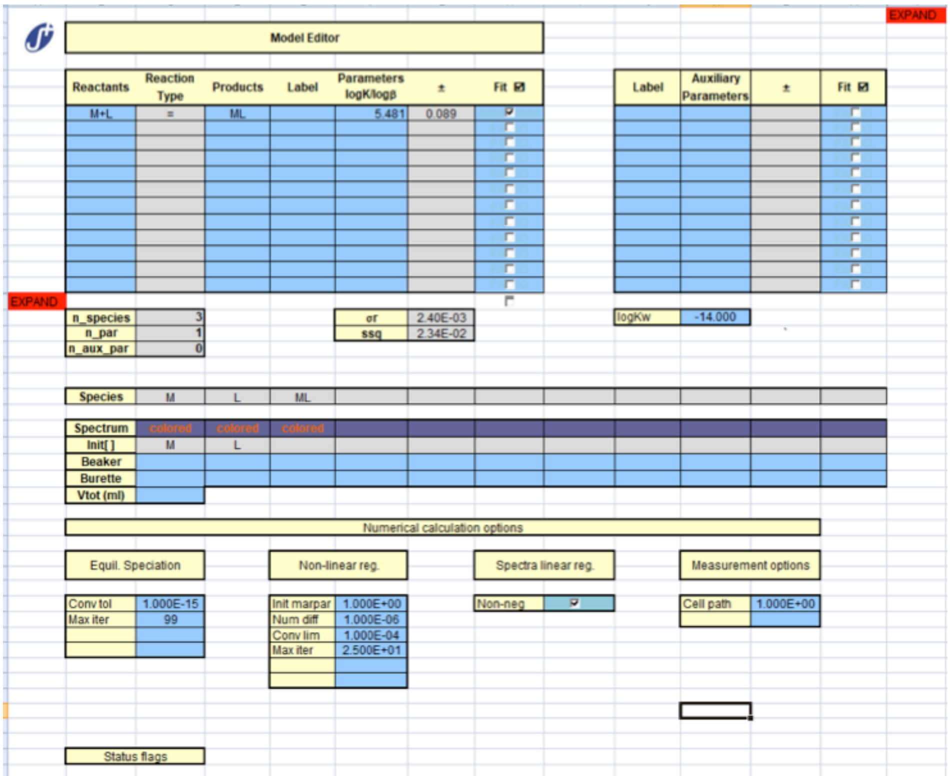


**Figure S7:** UV/Vis titration of **4** with **9** in [EMIM][FAP], ReactLab Input/Output spreadsheet displaying log($K$) =5.481.

## c) [EMIM][$BF_4$]

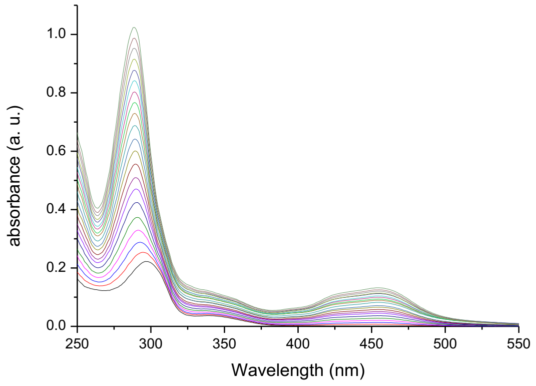


**Figure S8:** UV/Vis spectra of **9** (ca. 6 x $10^{-5}$ M) upon addition of **4** (0 → 2 equiv), while maintaining the concentration of **9** constant, in [EMIM][$BF_4$] at 298 K.

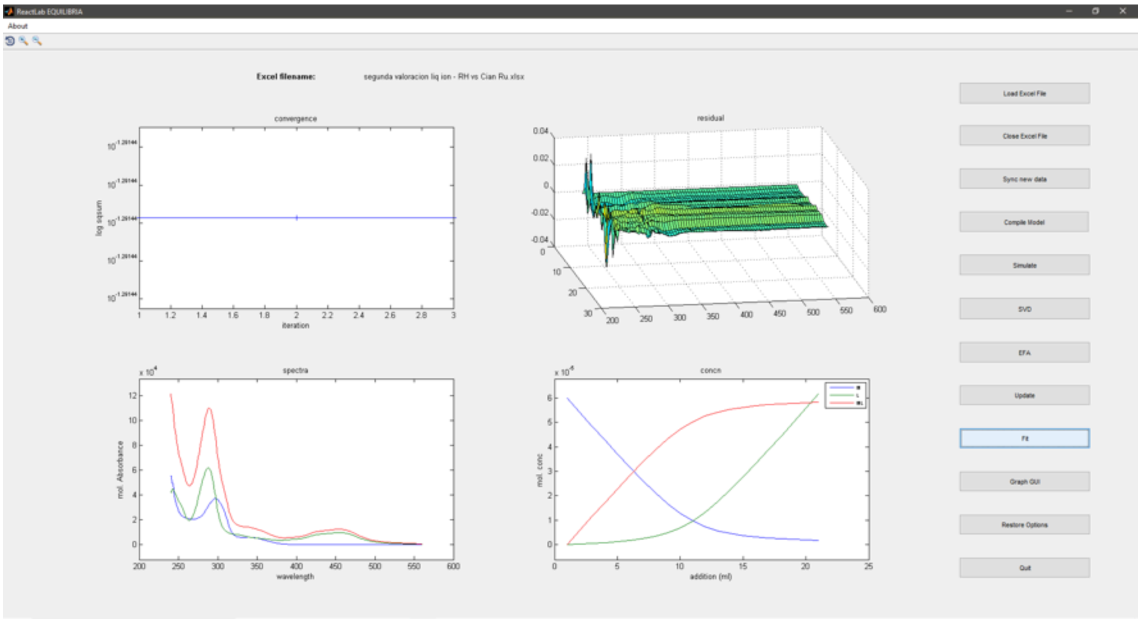


**Figure S9:** UV/Vis titration of **9** with **4** in [EMIM][$BF_4$], ReactLab Working Window (JplusConsulting, www.jplusconsulting.com). Top left, plot of least-squares optimization during a fit. Top right, a 3D plot of the residuals for the fit. Bottom left, the calculated spectrum for each species. Bottom right, concentration profile for the species in solution.

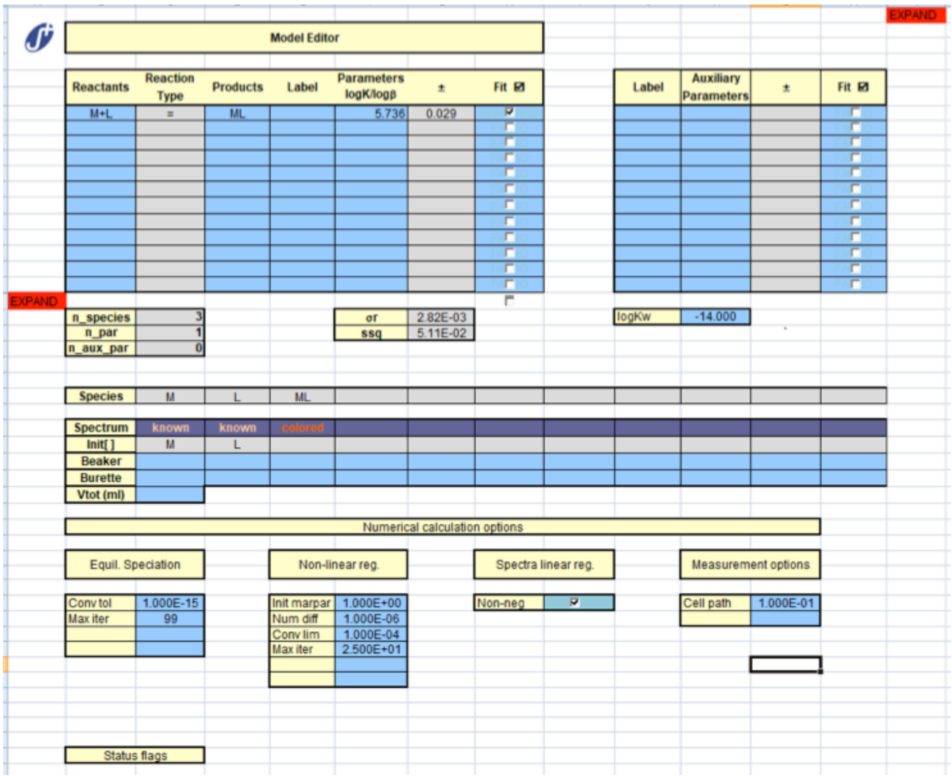


**Figure S10:** UV/Vis titration of **9** with **4** in [EMIM][FAP], ReactLab Input/Output spreadsheet displaying log($K$) =5.736.

## UV/Vis titrations experiments of couple 18-Crown-6 vs Am

### a) Acetonitrile

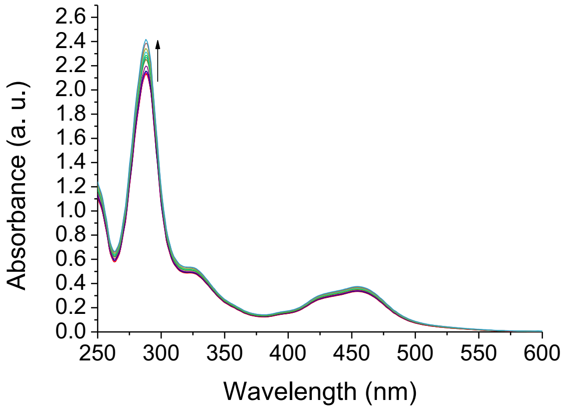


**Figure S11:** UV/Vis spectra of **12** (ca. 2.6 x $10^{-4}$ M) upon addition of **18_Crown_6** (0 → 2 equiv), while maintaining the concentration of **9** constant, in $CH_3CN$ at 298 K.

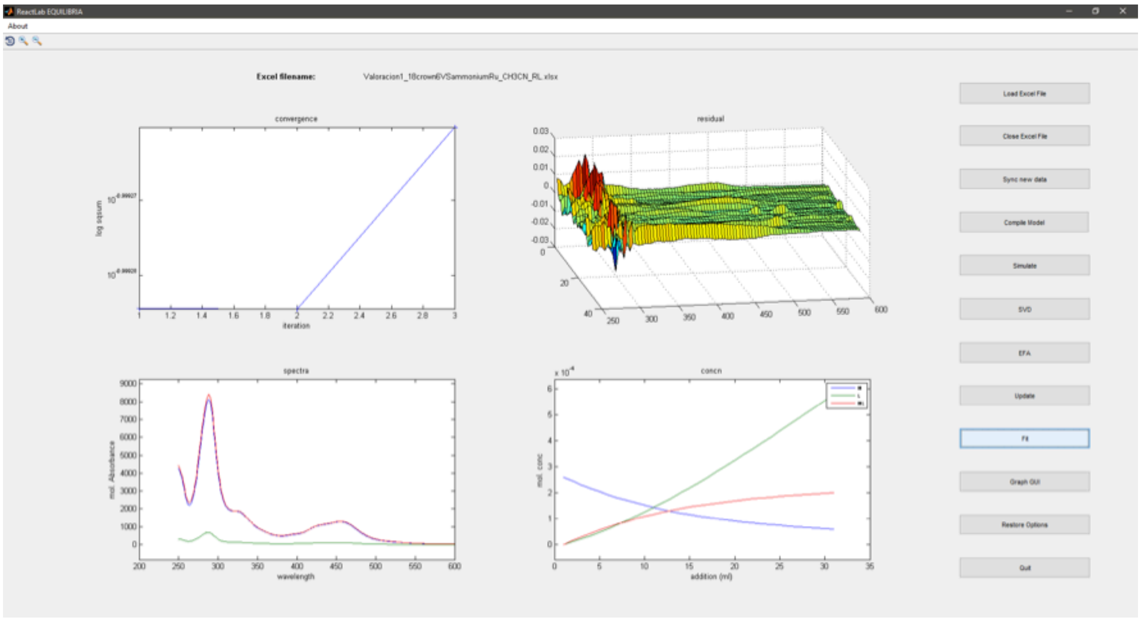


**Figure S12:** UV/Vis titration of **12** with **18_Crown_6** in $CH_3CN$, ReactLab Working Window (JplusConsulting, www.jplusconsulting.com). Top left, plot of least-squares optimization during a fit. Top right, a 3D plot of the residuals for the fit. Bottom left, the calculated spectrum for each species. Bottom right, concentration profile for the species in solution.

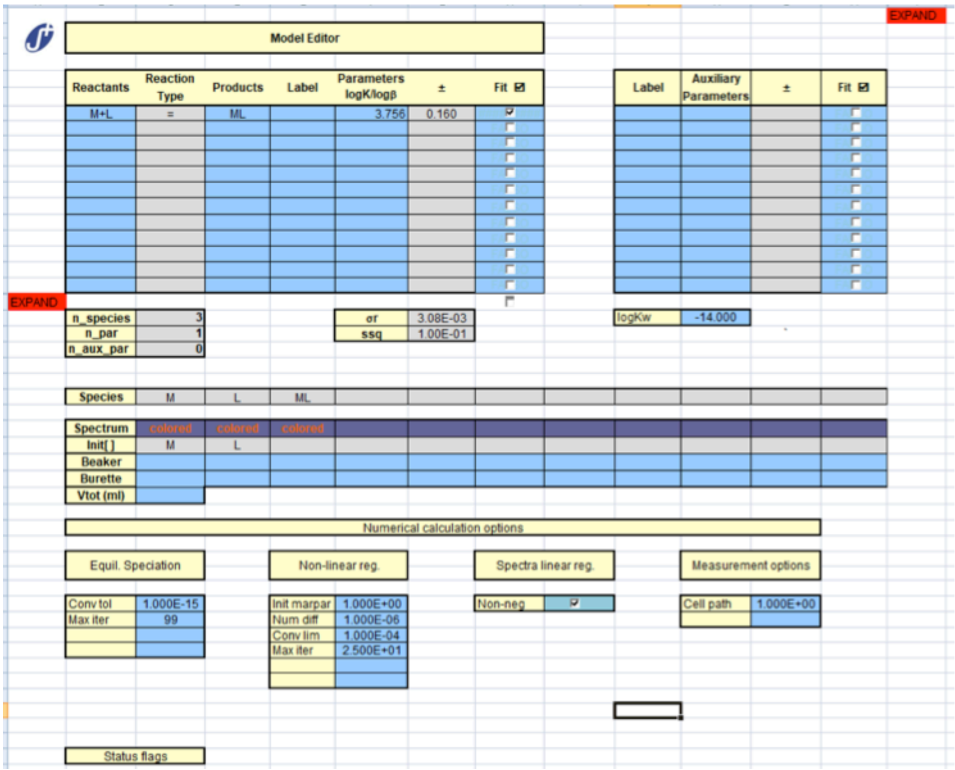


**Figure S13:** UV/Vis titration of **12** with **18_Crown_6** in $CH_3CN$, ReactLab Input/Output spreadsheet displaying log($K$) =3.756.

## b) [EMIM][FAP]

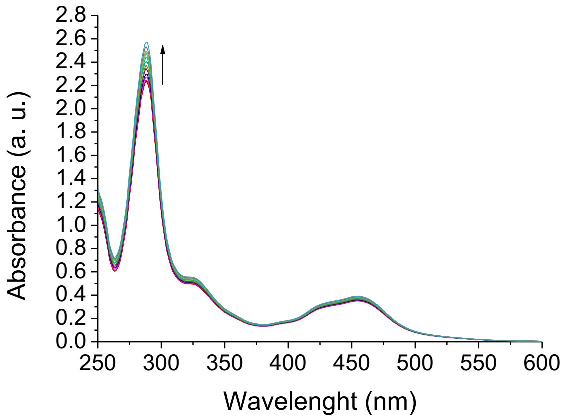


**Figure S14:** UV/Vis spectra of **12** (ca. 2.6 x $10^{-4}$ M) upon addition of **18_Crown_6** (0 → 2 equiv), while maintaining the concentration of **9** constant, in [EMIM][FAP] at 298 K.

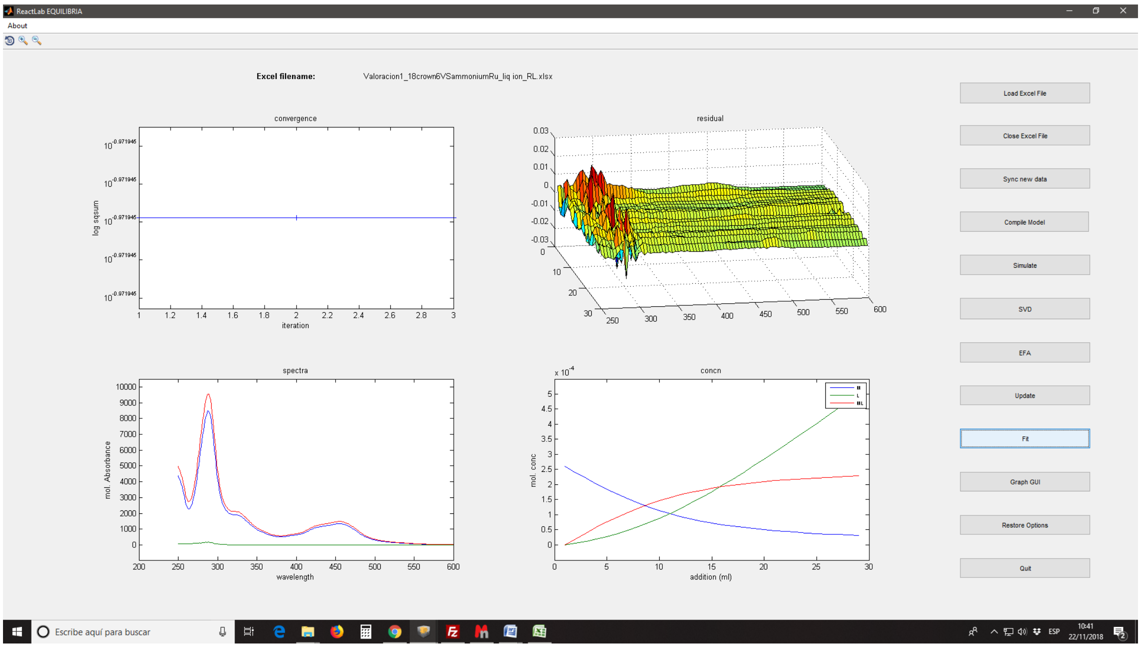


**Figure S15:** UV/Vis titration of **12** with **18_Crown_6** in [EMIM][FAP], ReactLab Working Window (JplusConsulting, www.jplusconsulting.com). Top left, plot of least-squares optimization during a fit. Top right, a 3D plot of the residuals for the fit. Bottom left, the calculated spectrum for each species. Bottom right, concentration profile for the species in solution.

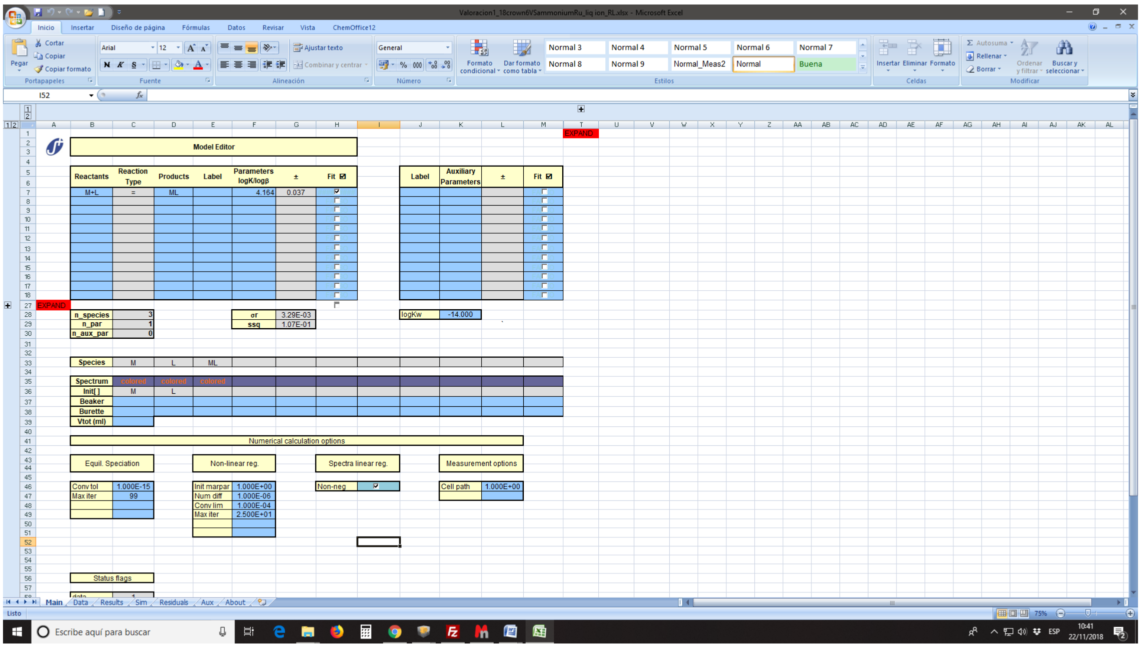

**Figure S16:** UV/Vis titration of **12** with **18_Crown_6** in [EMIM][FAP], ReactLab Input/Output spreadsheet displaying log($K$) =4.164.

## c) [EMIM][$BF_4$]

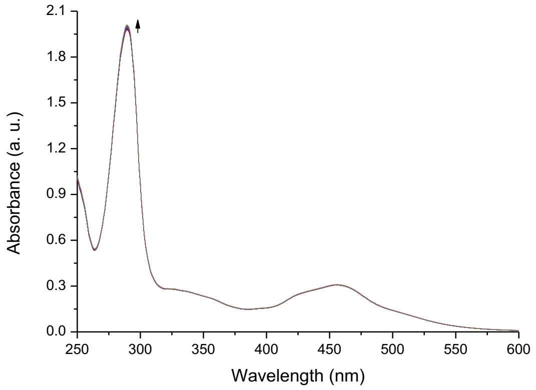


**Figure S17:** UV/Vis spectra of **12** (ca. 2.6 x $10^{-4}$ M) upon addition of **18_Crown_6** (0 → 2 equiv), while maintaining the concentration of **9** constant, in [EMIM][$BF_4$] at 298 K.

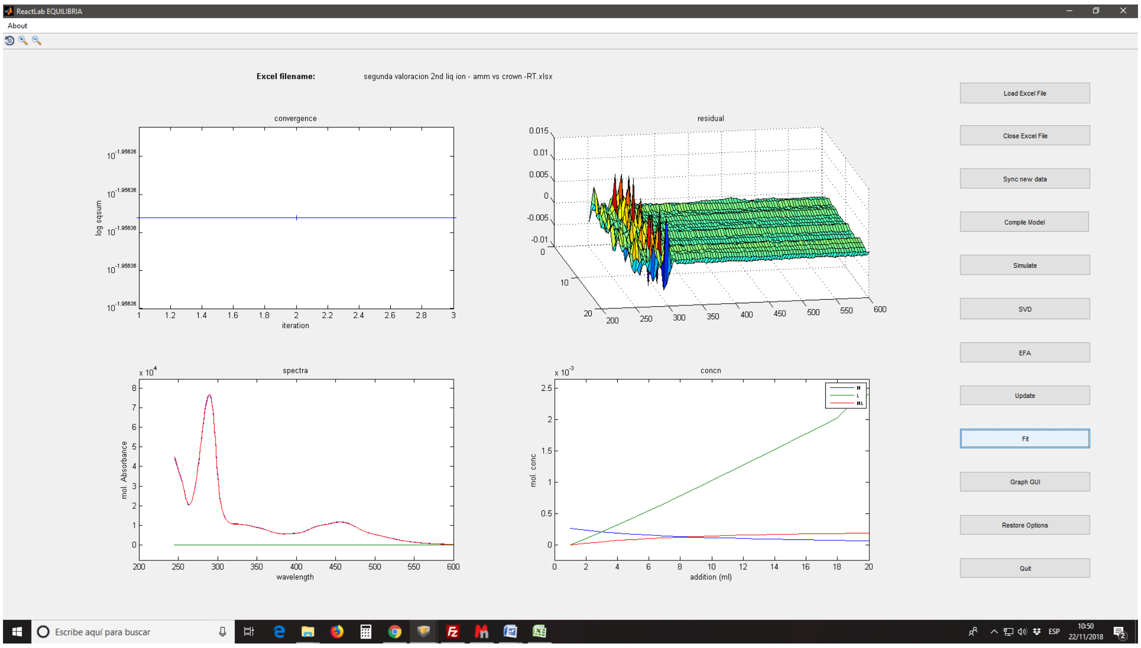


**Figure S18:** UV/Vis titration of **12** with **18_Crown_6** in [EMIM][$BF_4$], ReactLab Working Window (JplusConsulting, www.jplusconsulting.com). Top left, plot of least-squares optimization during a fit. Top right, a 3D plot of the residuals for the fit. Bottom left, the calculated spectrum for each species. Bottom right, concentration profile for the species in solution.

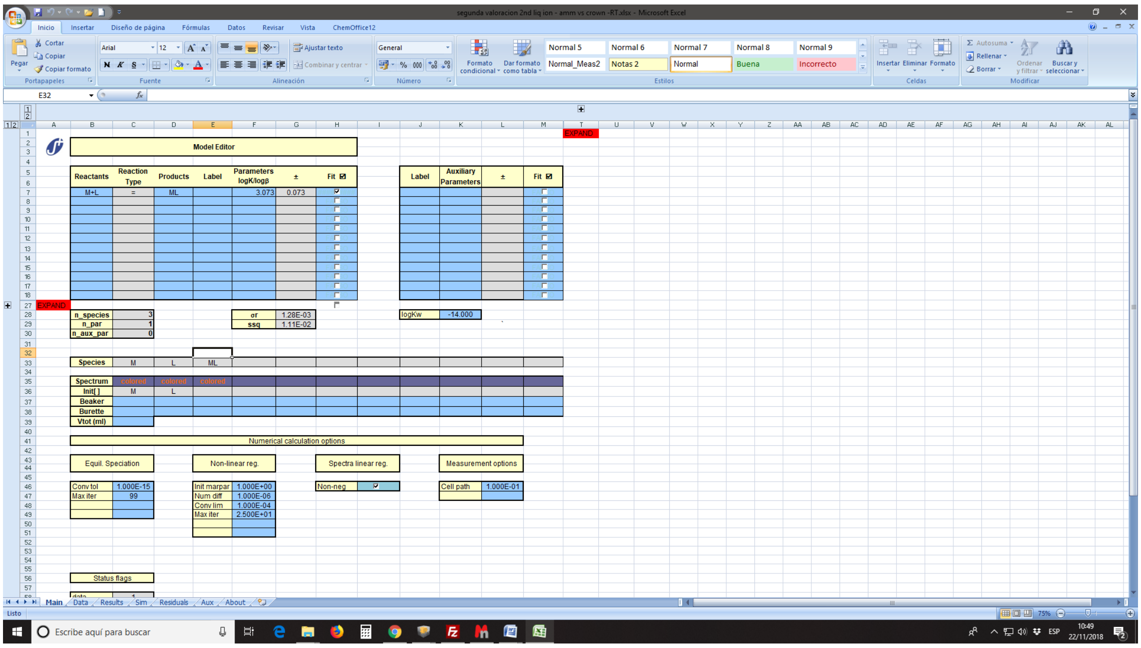


**Figure S19:** UV/Vis titration of **12** with **18_Crown_6** in [EMIM][$BF_4$], ReactLab Input/Output spreadsheet displaying log($K$) =3.073.

### UV/Vis titrations experiments of couple Benzamidine vs AcOH

### a) Chloroform

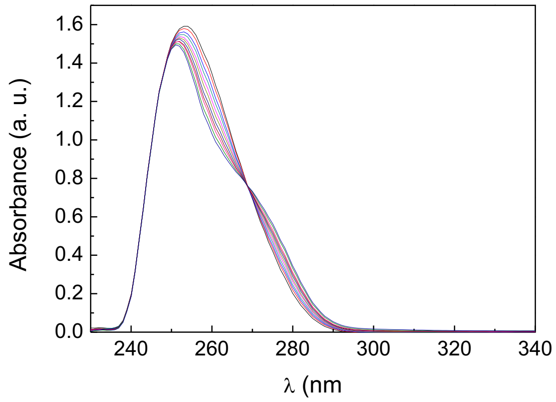


**Figure S20:** UV/Vis spectra of **Ben** (ca. 6.5 x $10^{-4}$ M) upon addition of **AcOH** (0 → 3 equiv), while maintaining the concentration of **Ben** constant, in chloroform at 298 K.

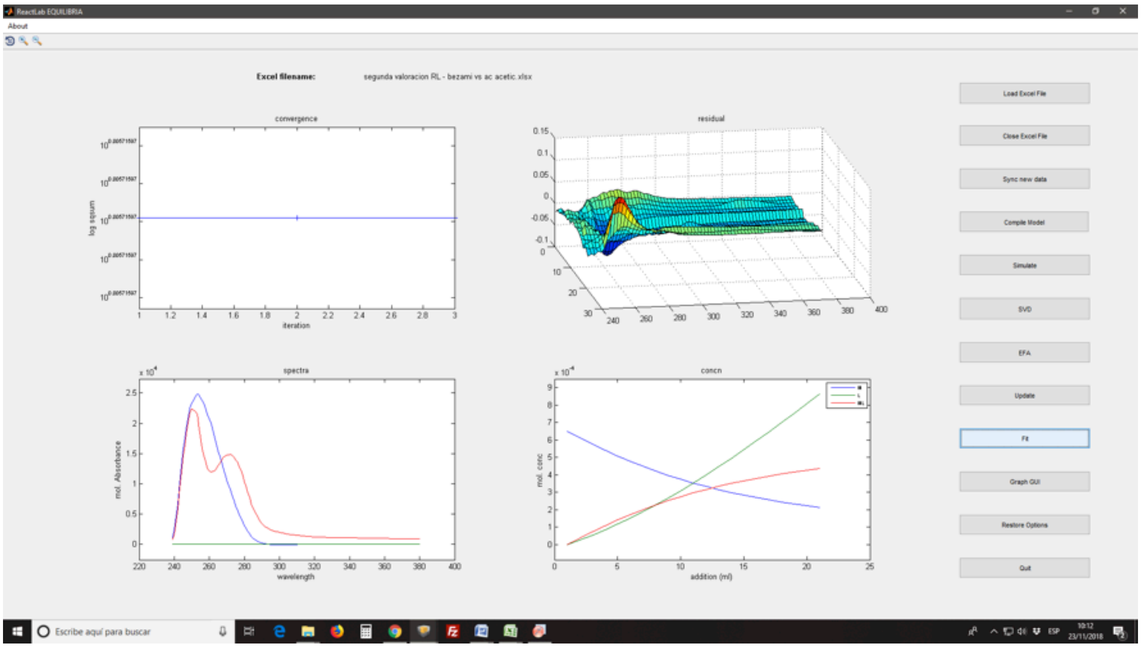


**Figure S21:** UV/Vis titration of **Ben** with **AcOH** in chloroform, ReactLab Working Window (JplusConsulting, www.jplusconsulting.com). Top left, plot of least-squares optimization during a fit. Top right, a 3D plot of the residuals for the fit. Bottom left, the calculated spectrum for each species. Bottom right, concentration profile for the species in solution.

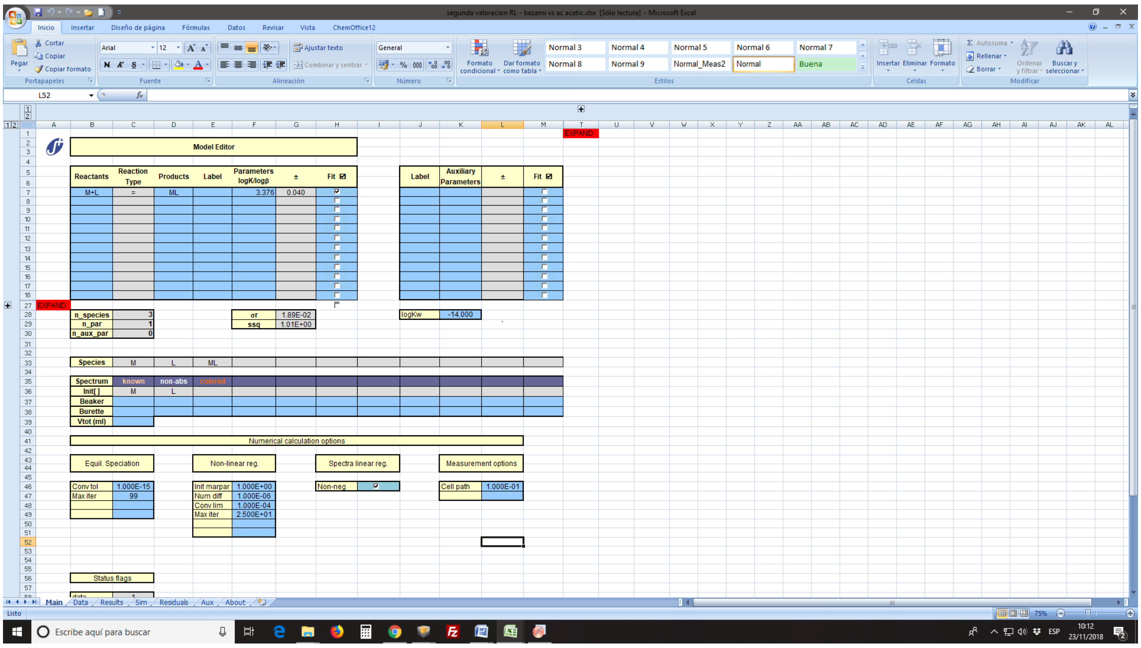


**Figure S22:** UV/Vis titration of **Ben** with **AcOH** in chloroform, ReactLab Input/Output spreadsheet displaying log($K$) =3.376.

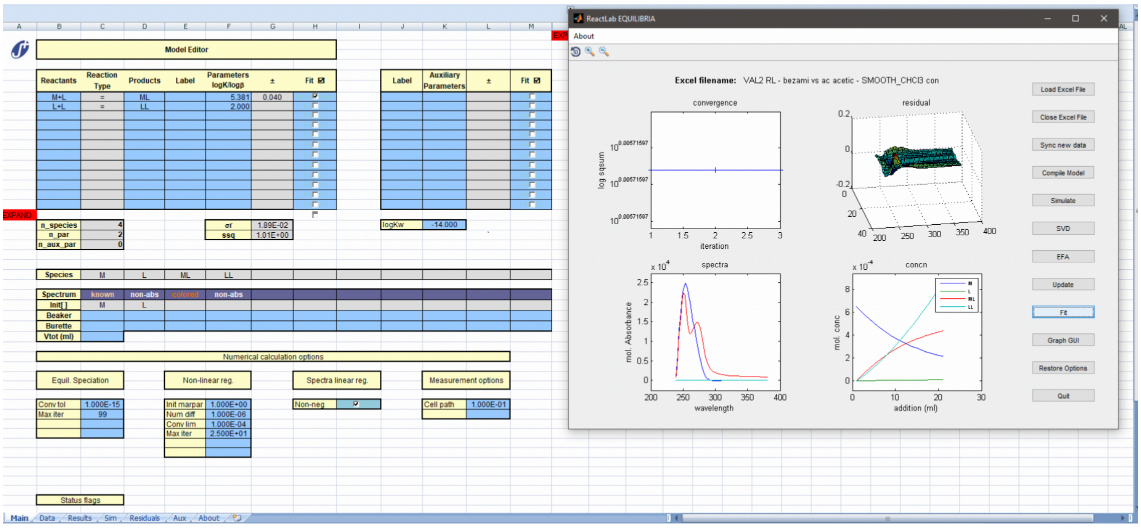


**Figure S23:** UV/Vis titration of **Ben** with **AcOH** in chloroform, analysed using log Kd = 2.0 for the AcOH dimerization, ReactLab Input/Output spreadsheet. Note that log($K$) increases to 5.381, but the fitting does not improve (see residuals).

## b) Acetonitrile

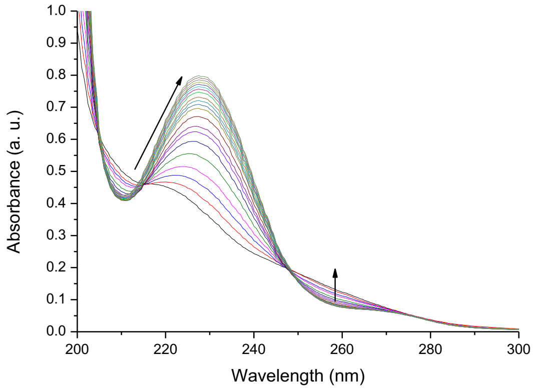


**Figure S24:** UV/Vis spectra of **Ben** (ca. 6.5 x $10^{-4}$ M) upon addition of **AcOH** (0 → 3 equiv), while maintaining the concentration of **Ben** constant, in $CH_3CN$ at 298 K.

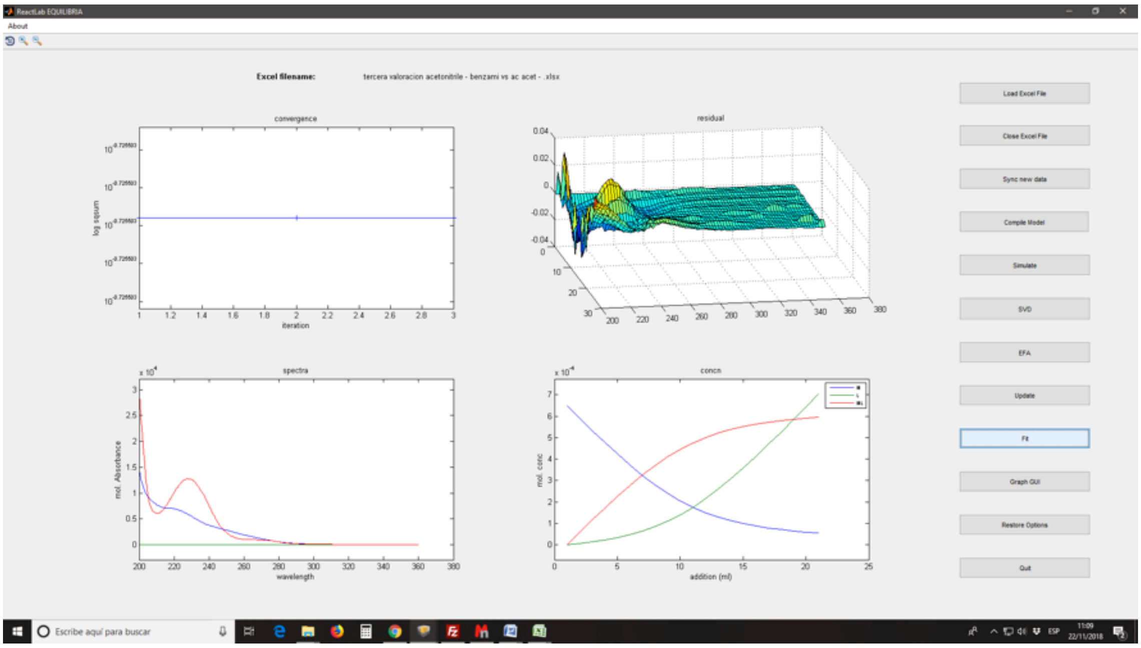

**Figure S25:** UV/Vis titration of **Ben** with **AcOH** in $CH_3CN$, ReactLab Working Window (JplusConsulting, www.jplusconsulting.com). Top left, plot of least-squares optimization during a fit. Top right, a 3D plot of the residuals for the fit. Bottom left, the calculated spectrum for each species. Bottom right, concentration profile for the species in solution.

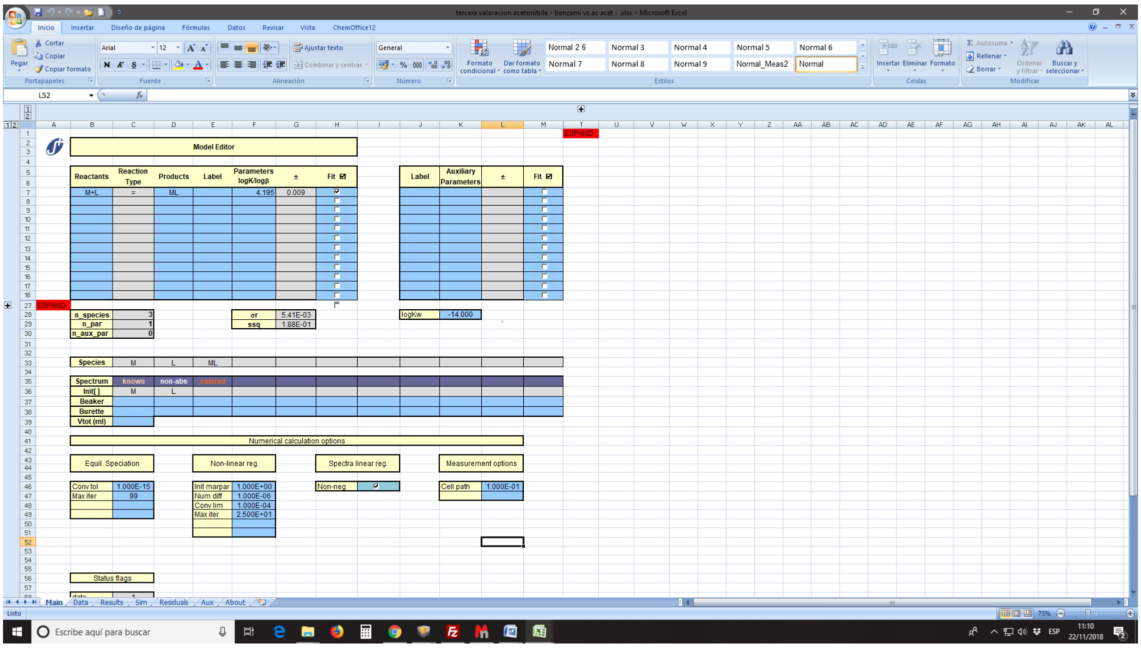


**Figure S26:** UV/Vis titration of **Ben** with **AcOH** in $CH_3CN$, ReactLab Input/Output spreadsheet displaying log($K$) =4.195.

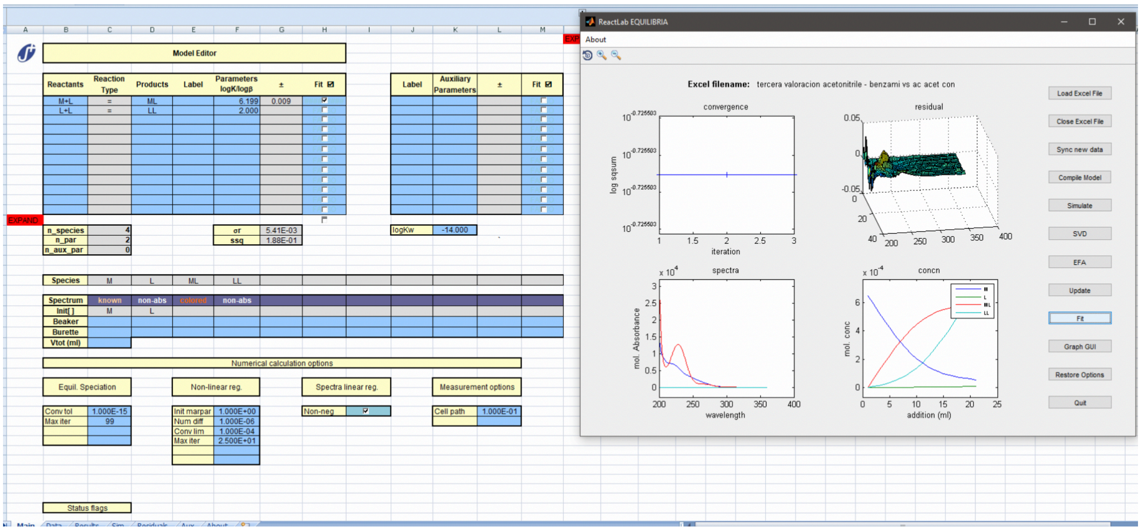


**Figure S27:** UV/Vis titration of **Ben** with **AcOH** in $CH_3CN$, analysed using log $K_d$ = 2.0 for the AcOH dimerization, ReactLab Input/Output spreadsheet. Note that log($K$) increases to 6.199, but the fitting does not improve (see residuals).

## c) [EMIM][FAP]

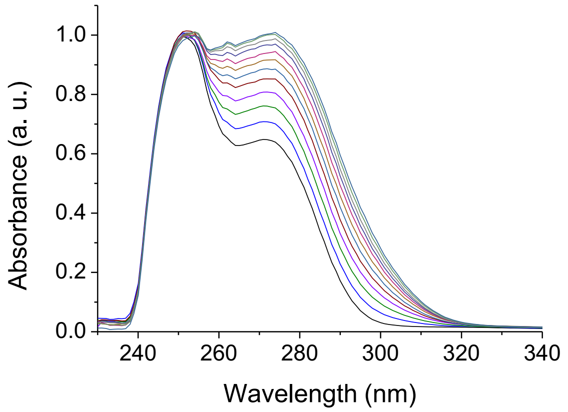


**Figure S28:** UV/Vis spectra of **Ben** (ca. 6.5 x $10^{-4}$ M) upon addition of **AcOH** (0 → 3 equiv), while maintaining the concentration of **Ben** constant, in [EMIM][FAP] at 298 K.

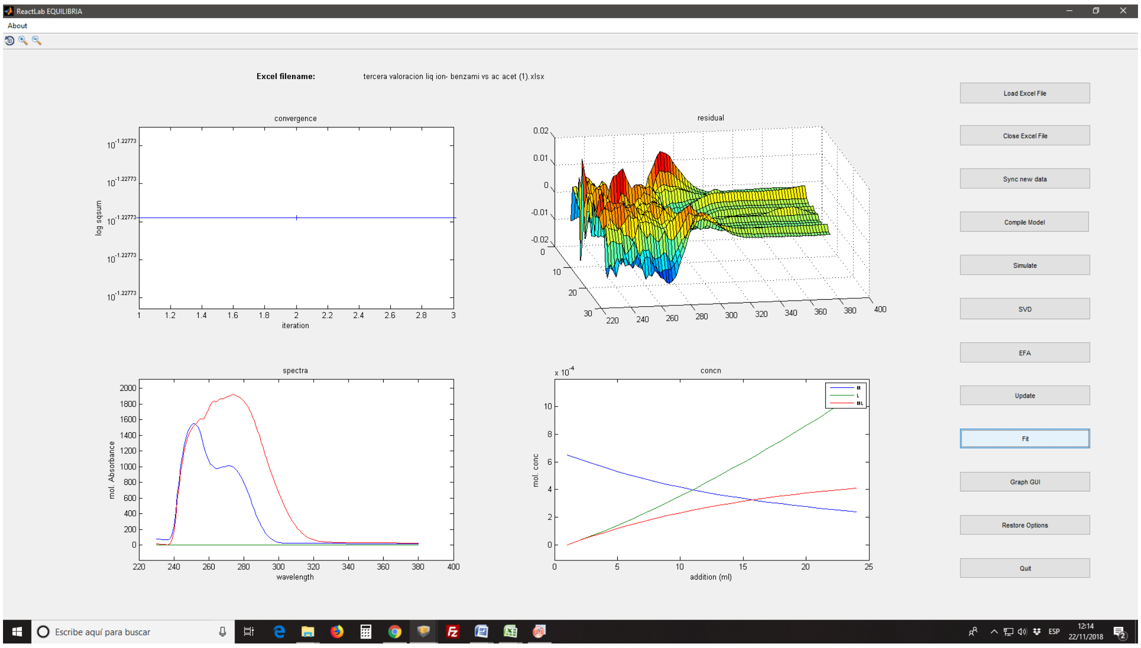


**Figure S29:** UV/Vis titration of **Ben** with **AcOH** in [EMIM][FAP], ReactLab Working Window (JplusConsulting, www.jplusconsulting.com). Top left, plot of least-squares optimization during a fit. Top right, a 3D plot of the residuals for the fit. Bottom left, the calculated spectrum for each species. Bottom right, concentration profile for the species in solution.

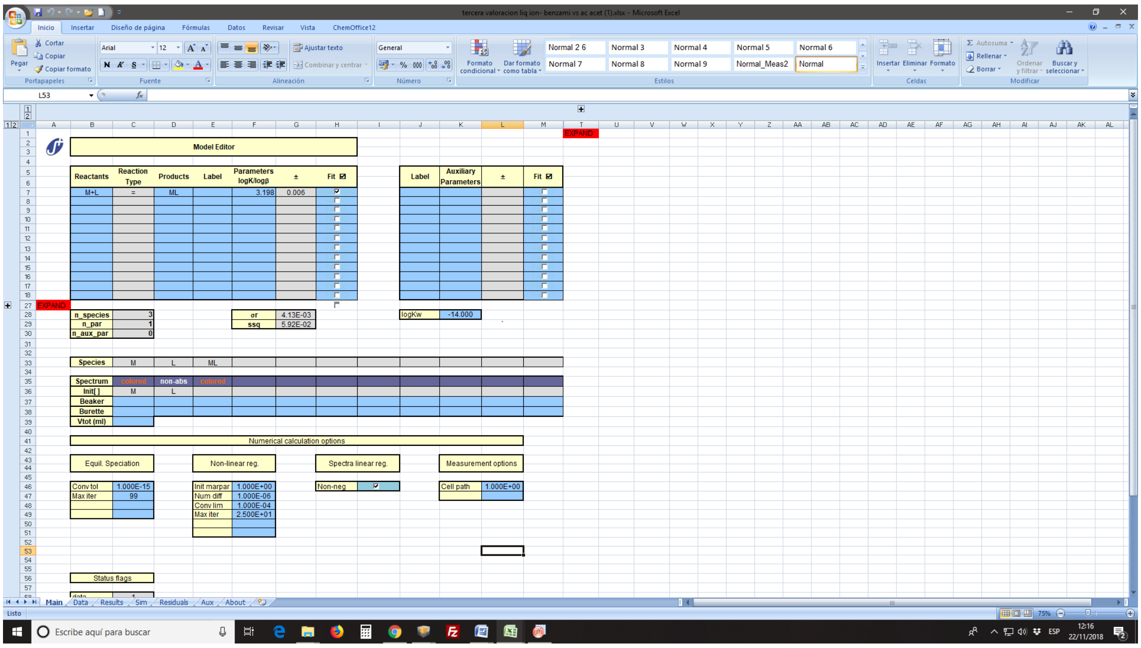

**Figure S30:** UV/Vis titration of **Ben** with **AcOH** in [EMIM][FAP], ReactLab Input/Output spreadsheet displaying log(*K*) =3.198.

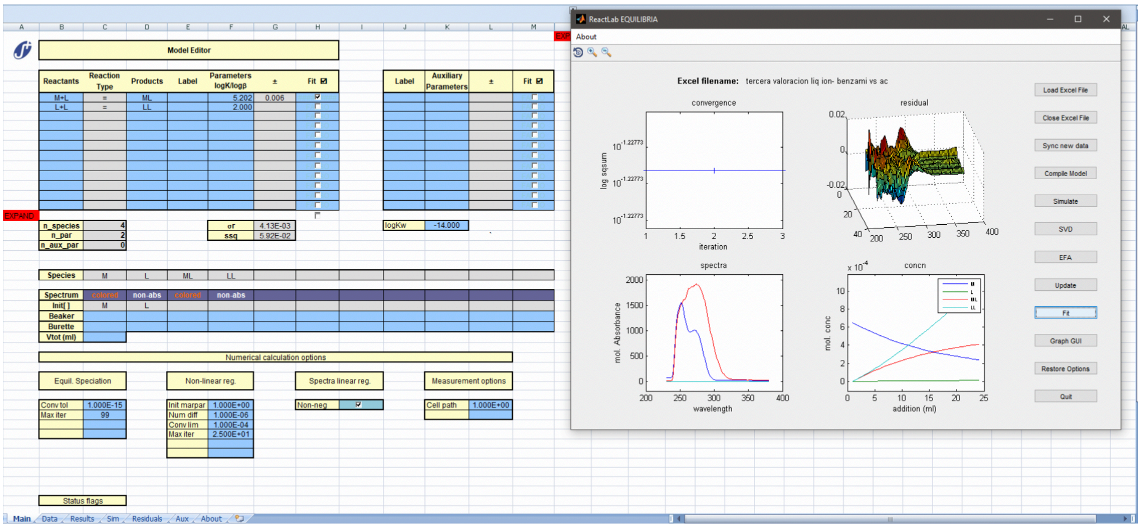

**Figure S31:** UV/Vis titration of **Ben** with **AcOH** in [EMIM][FAP], analysed using log Kd = 2.0 for the AcOH dimerization, ReactLab Input/Output spreadsheet. Note that log(*K*) increases to 5.202, but the fitting does not improve (see residuals).

## d) [EMIM]

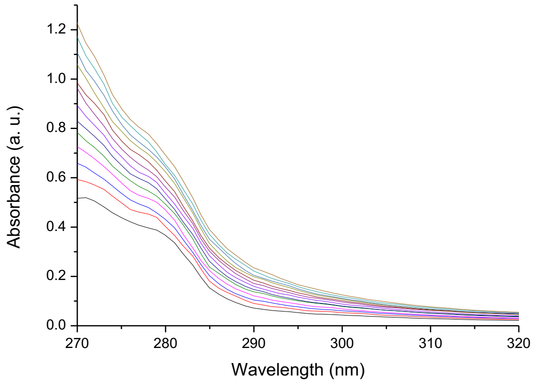


**Figure S32:** UV/Vis spectra of **Ben** (ca. 6.5 x $10^{-4}$ M) upon addition of **AcOH** (0 → 3 equiv), while maintaining the concentration of **Ben** constant, in $[EMIM][BF_4]$ at 298 K.

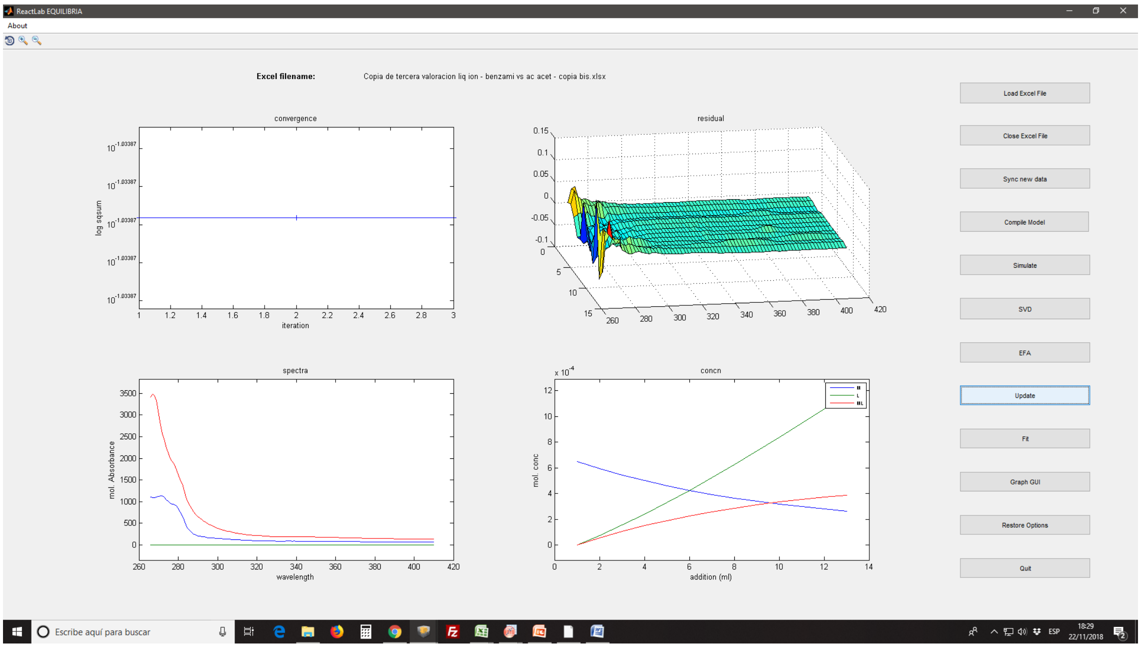


**Figure S33:** UV/Vis titration of **Ben** with **AcOH** in $[EMIM][BF_4]$, ReactLab Working Window (JplusConsulting, www.jplusconsulting.com). Top left, plot of least-squares optimization during a fit. Top right, a 3D plot of the residuals for the fit. Bottom left, the calculated spectrum for each species. Bottom right, concentration profile for the species in solution.

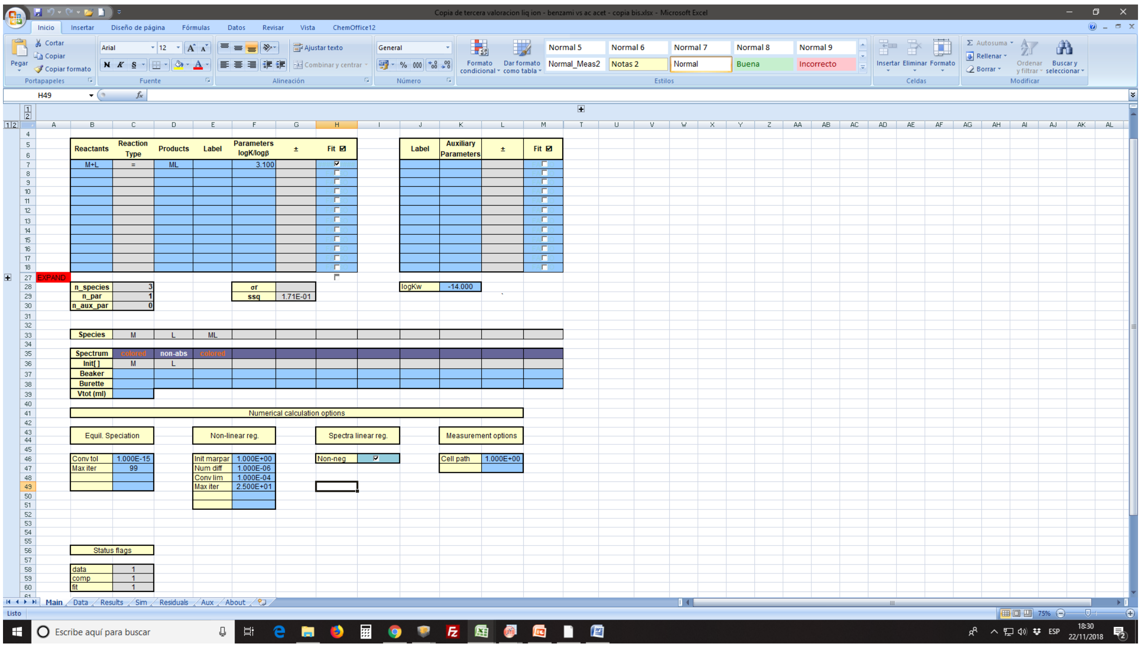

**Figure S34:** UV/Vis titration of **Ben** with **AcOH** in [EMIM][$BF_4$], ReactLab Input/Output spreadsheet displaying log($K$) =3.100.

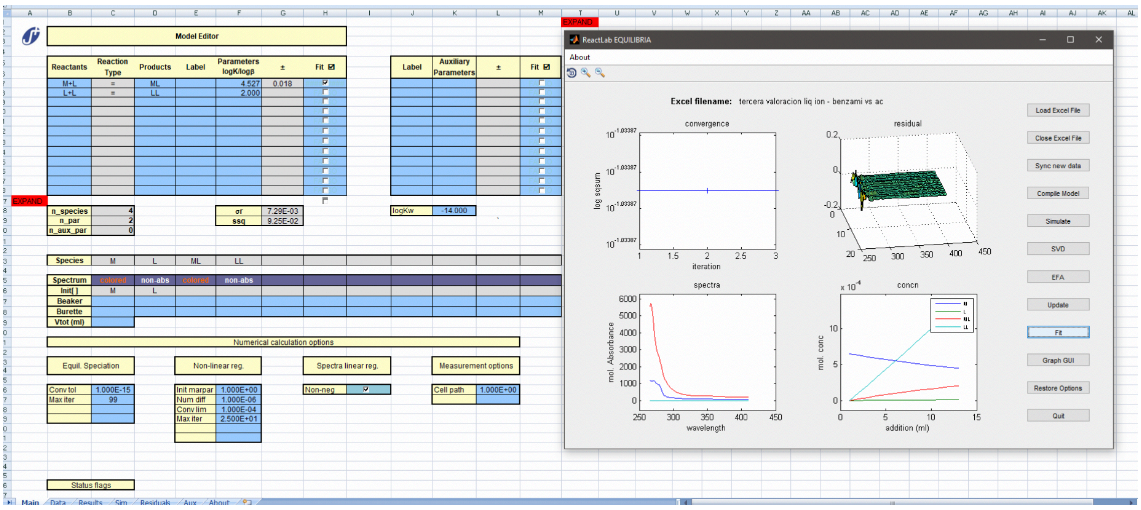

**Figure S35:** UV/Vis titration of **Ben** with **AcOH** in [EMIM][$BF_4$], analysed using log Kd = 2.0 for the AcOH dimerization, ReactLab Input/Output spreadsheet. Note that log($K$) increases to 4.527, but the fitting does not improve (see residuals).

## UV/Vis titrations experiments of couple DMTU vs BzO$^-$

## a) Acetonitrile

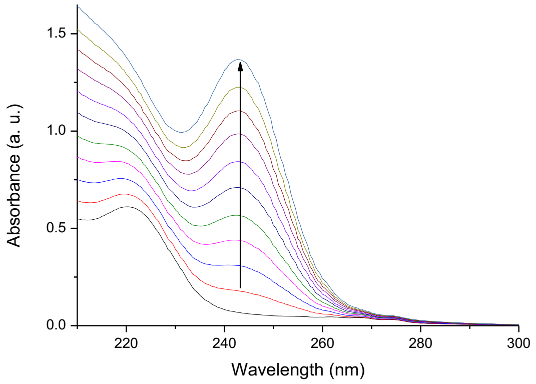


**Figure S36:** UV/Vis spectra of **BzO**$^-$ (ca. 6.5 x $10^{-4}$ M) upon addition of **DMTU** (0 → 3 equiv), while maintaining the concentration of **Ben** constant, in $CH_3CN$ at 298 K.

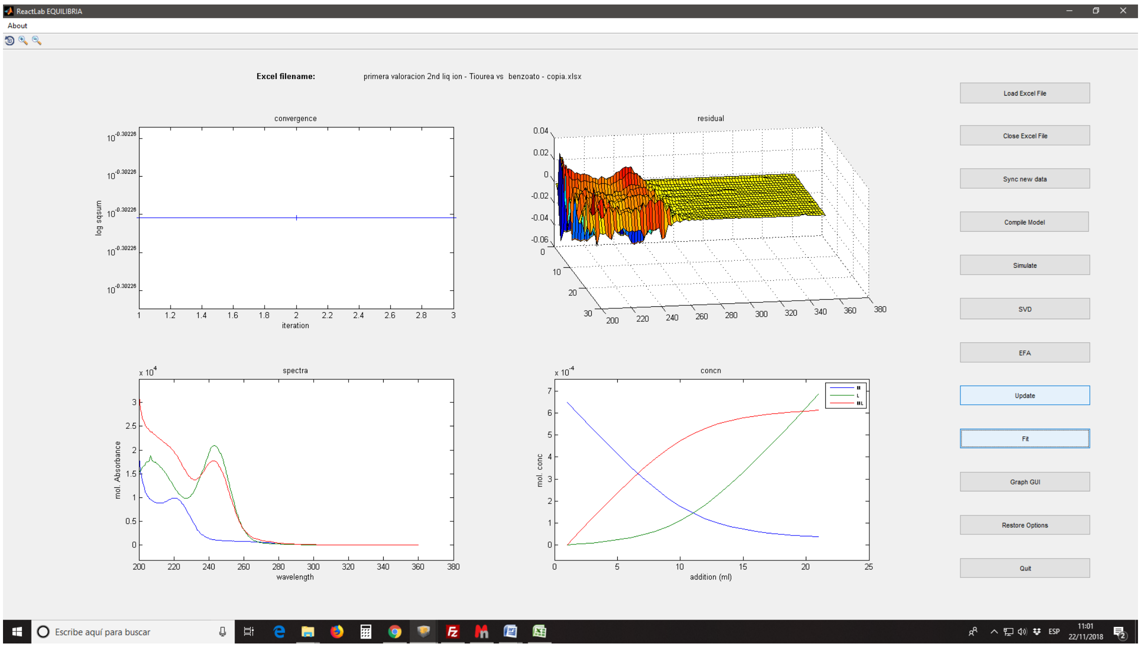


**Figure S37:** UV/Vis titration of **BzO**$^-$ with **DMTU** in $CH_3CN$, ReactLab Working Window (JplusConsulting, www.jplusconsulting.com). Top left, plot of least-squares optimization during a fit. Top right, a 3D plot of the residuals for the fit. Bottom left, the calculated spectrum for each species. Bottom right, concentration profile for the species in solution.

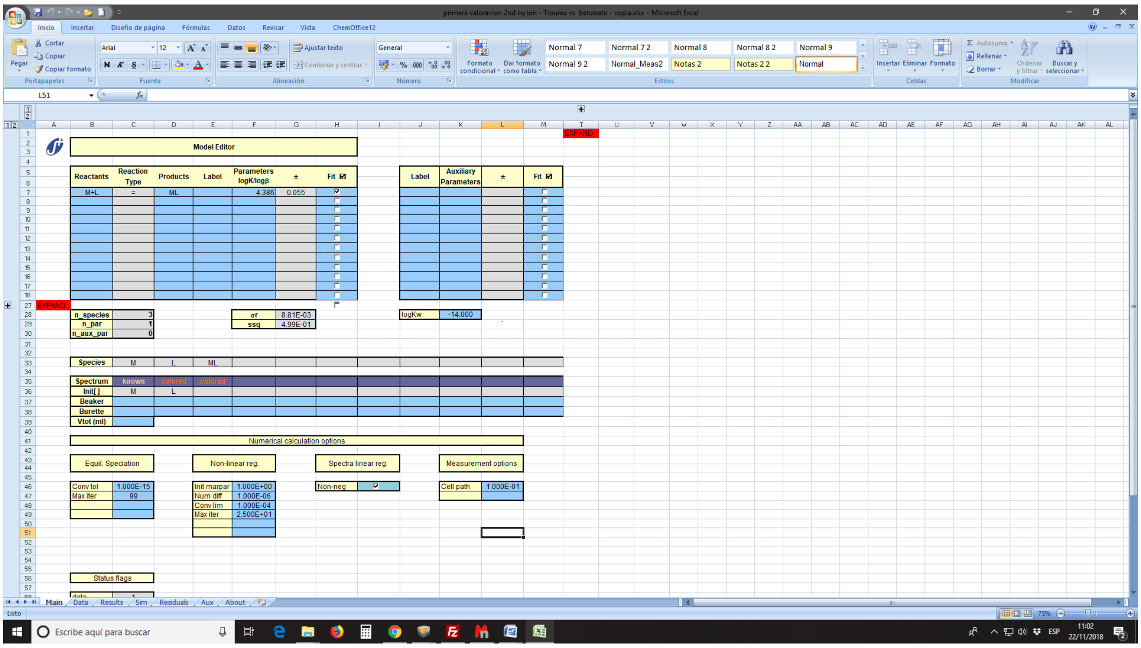


**Figure S38:** UV/Vis titration of **BzO**$^-$ with **DMTU** in $CH_3CN$, ReactLab Input/Output spreadsheet displaying log($K$) =4.386.

## b) Chloroform

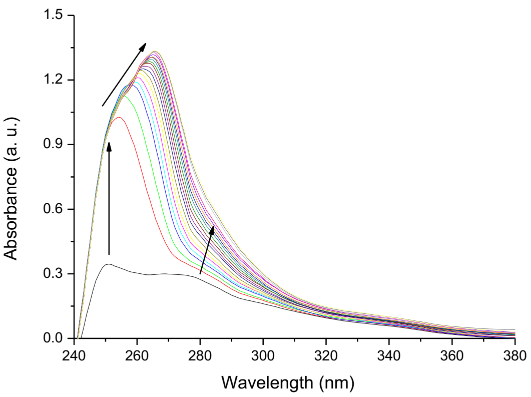


**Figure S39:** UV/Vis spectra of **BzO**$^-$ (ca. 1 x $10^{-3}$ M) upon addition of **DMTU** (0 → 2 equiv), while maintaining the concentration of **Ben** constant, in chloroform at 298 K.

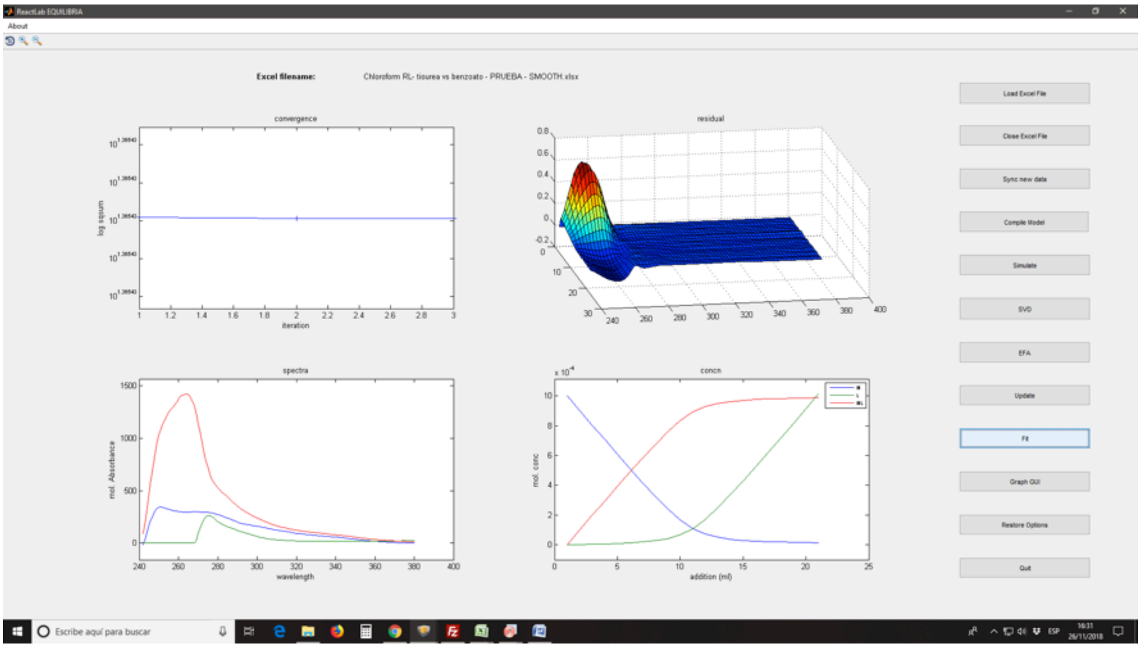


**Figure S40:** UV/Vis titration of **BzO**$^{-}$ with **DMTU** in chloroform, ReactLab Working Window (JplusConsulting, www.jplusconsulting.com). Top left, plot of least-squares optimization during a fit. Top right, a 3D plot of the residuals for the fit. Bottom left, the calculated spectrum for each species. Bottom right, concentration profile for the species in solution.

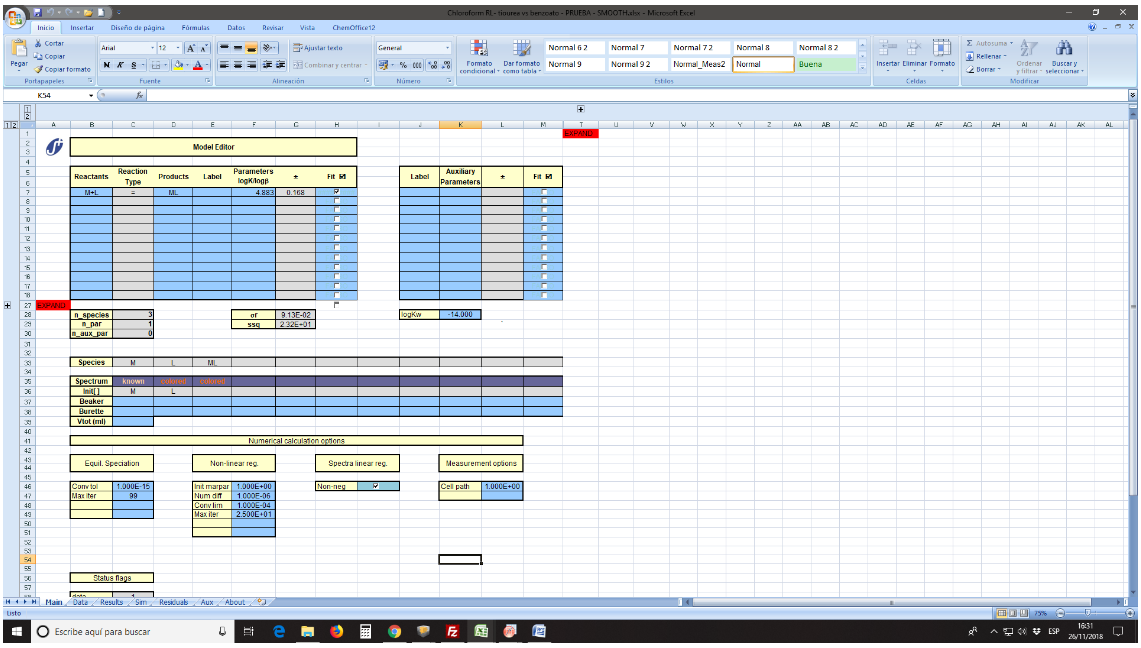


**Figure S41:** UV/Vis titration of **BzO**$^{-}$ with **DMTU** in chloroform, ReactLab Input/Output spreadsheet displaying log($K$) =4.883.

## c) [EMIM][FAP]

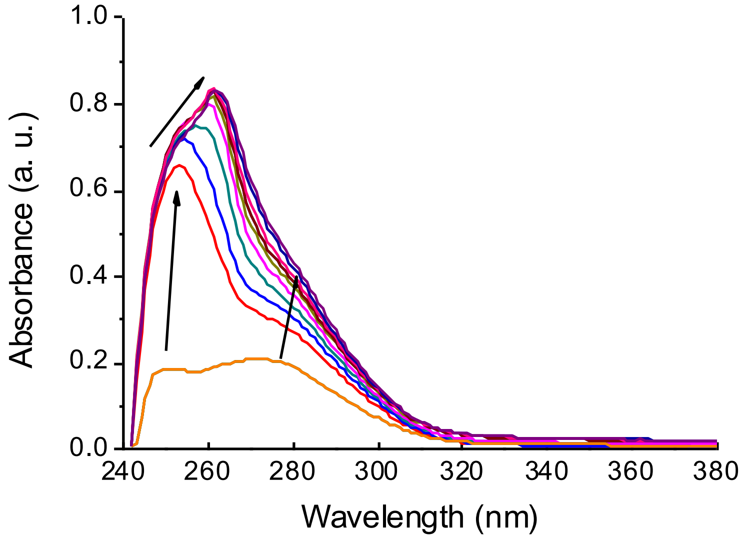


**Figure S42:** UV/Vis spectra of **BzO**$^-$ (ca. 1 x $10^{-3}$ M) upon addition of **DMTU** (0 → 2 equiv), while maintaining the concentration of **Ben** constant, in [EMIM][FAP] at 298 K.

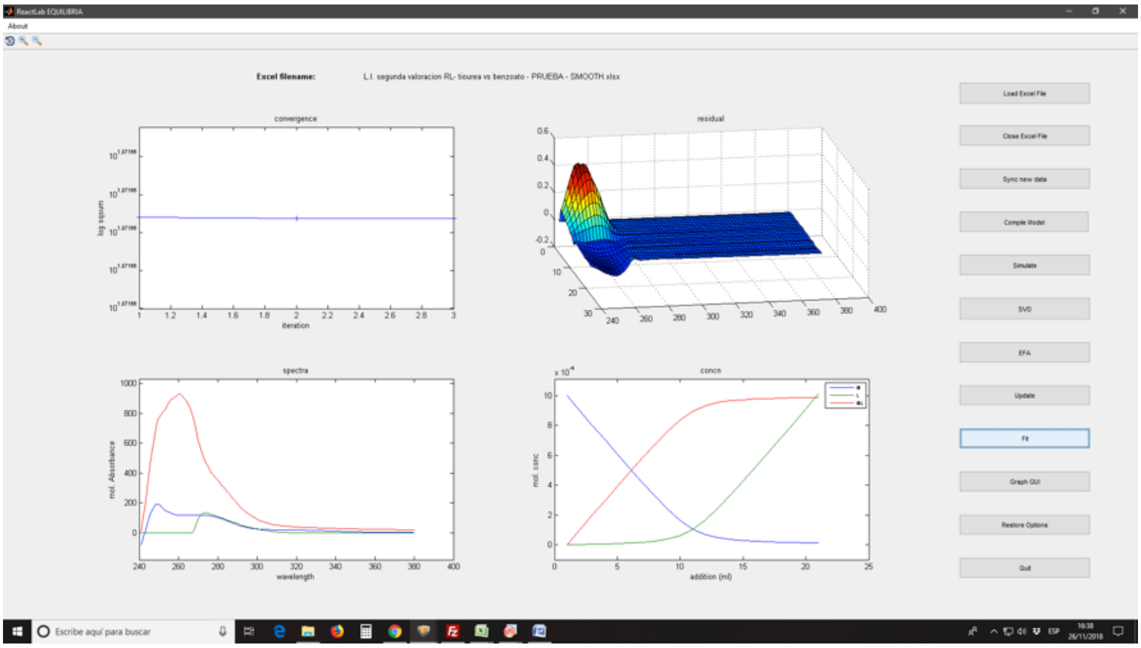


**Figure S43:** UV/Vis titration of **BzO**$^-$ with **DMTU** in [EMIM][FAP], ReactLab Working Window (JplusConsulting, www.jplusconsulting.com). Top left, plot of least-squares optimization during a fit. Top right, a 3D plot of the residuals for the fit. Bottom left, the calculated spectrum for each species. Bottom right, concentration profile for the species in solution.

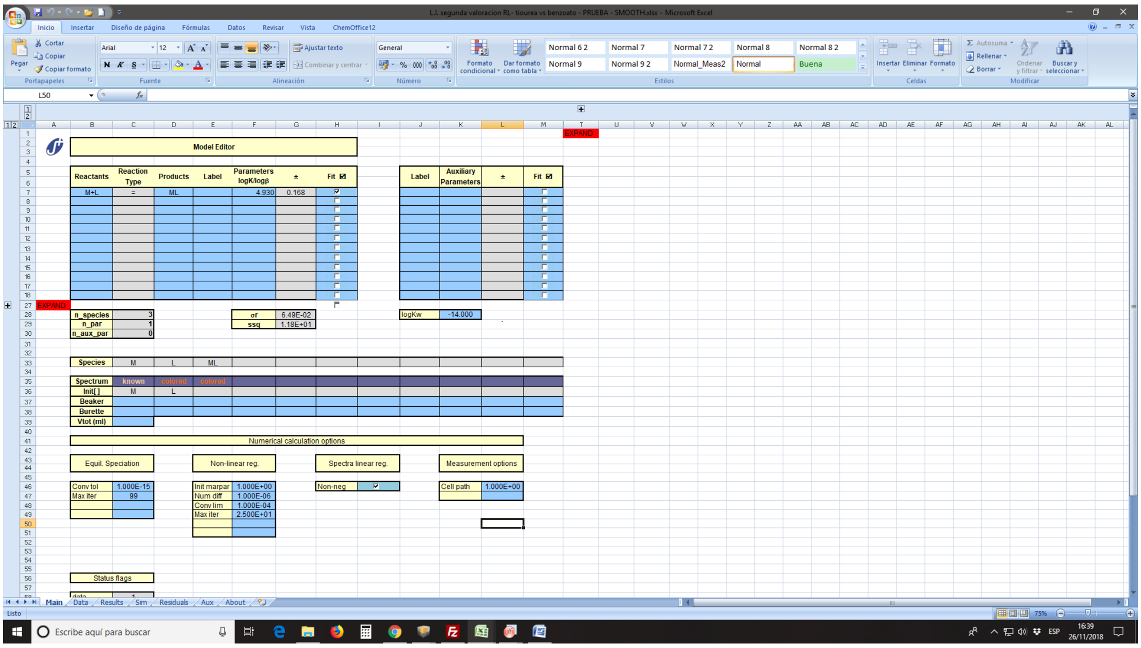


**Figure S44:** UV/Vis titration of **BzO**$^{-}$ with **DMTU** in [EMIM][FAP], ReactLab Input/Output spreadsheet displaying log($K$) =4.930.

## d) [EMIM][$BF_4$]

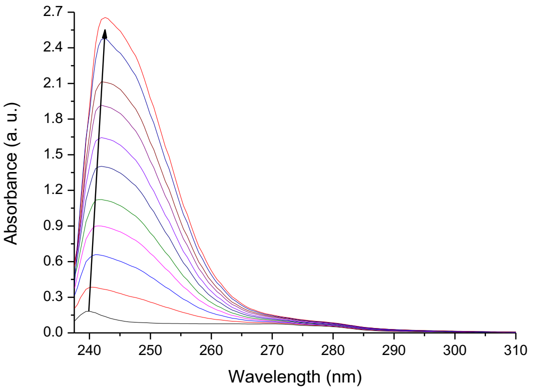


**Figure S45:** UV/Vis spectra of **BzO**$^{-}$ (ca. 1 x $10^{-3}$ M) upon addition of **DMTU** (0 → 2 equiv), while maintaining the concentration of **Ben** constant, in [EMIM][$BF_4$] at 298 K.

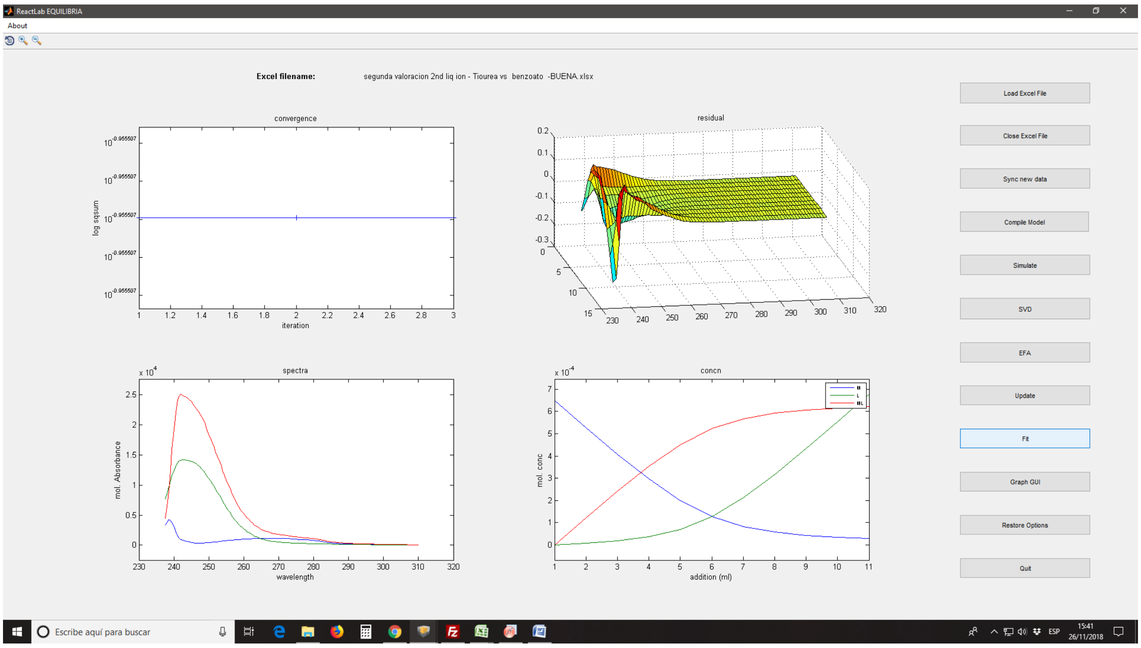


**Figure S46:** UV/Vis titration of **BzO**$^-$ with **DMTU** in [EMIM][$BF_4$], ReactLab Working Window (JplusConsulting, www.jplusconsulting.com). Top left, plot of least-squares optimization during a fit. Top right, a 3D plot of the residuals for the fit. Bottom left, the calculated spectrum for each species. Bottom right, concentration profile for the species in solution.

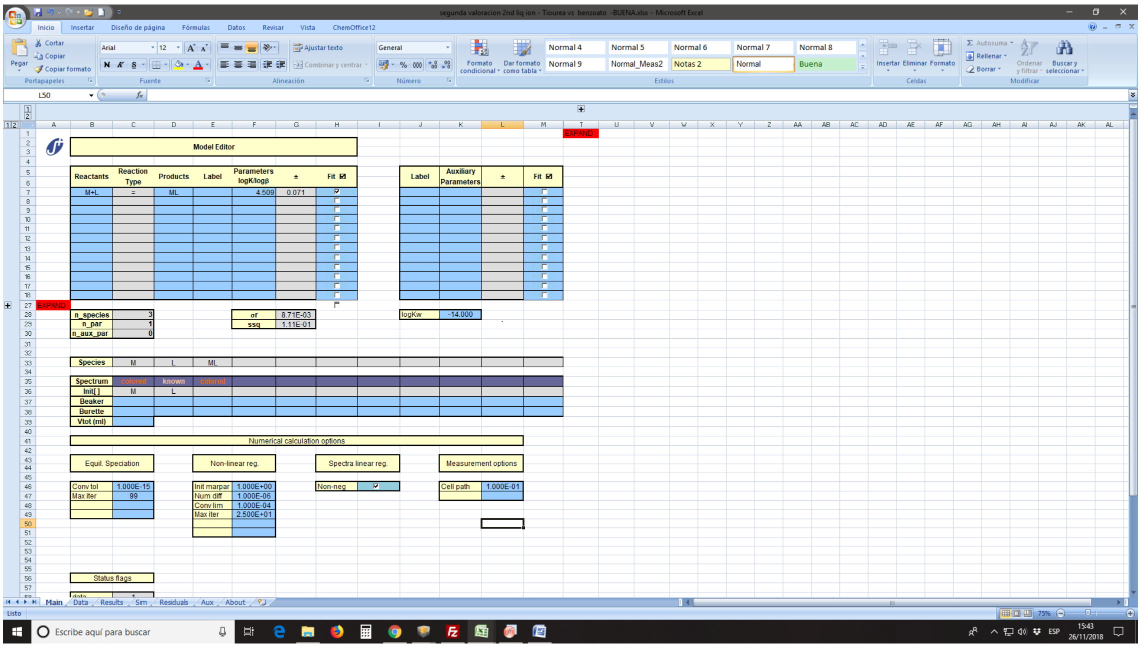


**Figure S47:** UV/Vis titration of **BzO**$^-$ with **DMTU** in [EMIM][$BF_4$], ReactLab Input/Output spreadsheet displaying log($K$) =4.509.

## 6. Description of ATR-IR experiments with RTIL

ATR-IR measurements were carried out on a Bruker's ALPHA-P FTIR spectrometer for the HR-cy and the Ben-AcOH couples. For the first couple, HR-cy, 1.4 x $10^{-3}$ M solutions in RTIL of HR and cy were prepared separately, then 1:1 molar mixture of both compounds, HR-cy at 7.0 $10^{-3}$ M was

For the second couple, Ben-AcOH, 0.2 M solutions in RTIL of Ben and AcOH were prepared separately, then 1:1 molar mixture of both compounds, HR-cy at 0.1 M was measured. The results are shown in Figure S48 below.

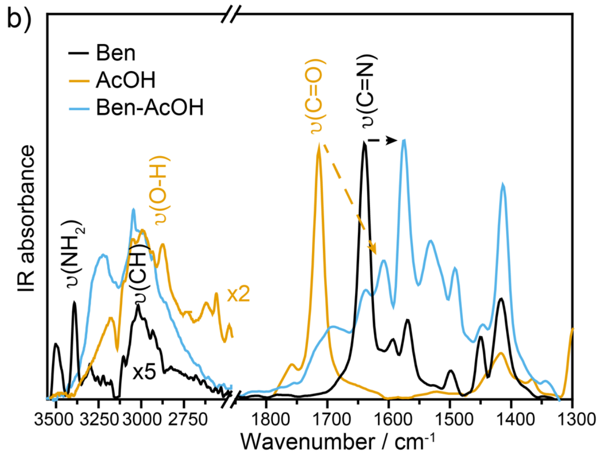


**Figure S48.** ATR-IR spectra in [emim][FAP] of Ben (black), AcOH (orange) and the 1:1 molar mixture of both compounds Ben-AcOH (blue) at 0.1 M. The spectrum of the solvent ([emim][FAP]) has been subtracted.

The solvent-corrected spectra of Ben, AcOH and the 1:1 molar mixture of both compounds, Ben-AcOH at 0.1 M using [emim][FAP] as solvent is depicted in Figure 3b. The IR spectrum of pure acetic acid is the typical spectrum of the homodimer.[9-11] A broad spectral feature around 3045 $cm^{-1}$ is observed, assigned to H-bonded OH stretching of a carboxylic acid. Between 2550 and 2800 $cm^{-1}$, we observe three bands assigned as a combination of low energy modes of a cyclic dimer ring with the C-OH bending overtone. In the fingerprint region, the spectrum is dominated by an intense band centred at 1717 $cm^{-1}$, assigned to C=O stretching of the carboxylic acid. For Ben, in the region of 3500-3000 $cm^{-1}$ we can observe the asymmetric and symmetric N-H stretches of the -$NH_2$ group at 3518 and 3422 $cm^{-1}$ respectively. The broad band around 3060 $cm^{-1}$ is associated to the C-H stretch of the aromatic moiety. In the fingerprint, we observe a strong band associated to C=N stretching at 1640 $cm^{-1}$ and at 1572 $cm^{-1}$ a medium

intensity band assigned to $NH_2$ scissoring. In analogy with the previous example, a new scenario appears for the 1:1 equimolar mixture: the spectrum is not the sum of the parent spectra. The bands associated with a cyclic dimer of AcOH and the N-H stretching of the $-NH_2$ group of Ben are no longer visible. Instead, we observe a broad vibrational spectrum between 3400 and 2700 $cm^{-1}$ as is expected for a doubly H-bonded dimer, with a peak centred at around 3010 $cm^{-1}$.

This broad band is red-shifted with respect to the band of the AcOH homodimer, indicating stronger H-bonds. Both C=X stretching (where X = N or O) undergo a red-shift in frequencies, from 1717 to 1609 $cm^{-1}$ for the C=O and from 1640 to 1578 $cm^{-1}$ for the C=N, as a second clear indication of the formation of strong H-bonds between both compounds.

In all the recorded spectra the solvent (RTIL) has been subtracted. Measurements of a small droplet placed on top of the diamond crystal was carried out by coading 254 scans.

### 6.b ATR-IR experiment of [EMIM][FAP] in presence of water vapor

It is important to compare these results with the case of weaker H-bond donors and acceptors, such as water mentioned previously in the main text. The ATR spectrum of the RTIL in the presence of water vapor is depicted in Figure S49.

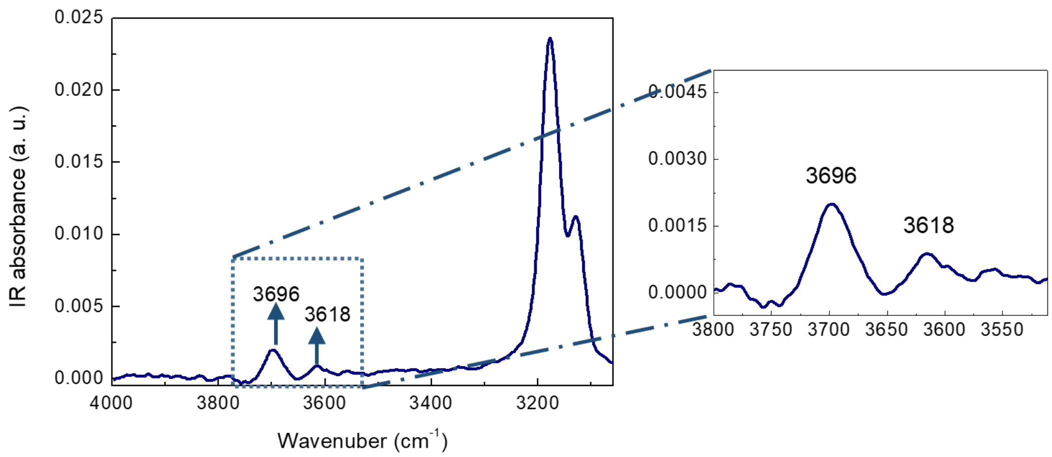


**Figure S49**. ATR-IR spectra of RTIL in the ν(O-H) region showing the bands of water dissolved in RTIL at 3696 and 3618 $cm^{-1}$.

It is important to stress that here we investigated the molecular state of water based on the positions of the IR bands of water rather than by comparing their concentrations based on the intensities of these bands. The IR bands corresponding to the antisymmetric ($\nu_3$) and symmetric ($\nu_1$) stretching modes of water usually lie in the region 3000-3800 $cm^{-1}$, the position and the intensity of these bands are very sensitive to the water environment and to the state of water association via H-bonds. The $\nu_3$ and $\nu_1$ bands

in water vapor absorb at 3756 and 3657 $cm^{-1}$ respectively; [12, 13] these bands shift to lower wavenumber when water interacts with the environment. (e.g. water dissolved in a solvent). The difference in the maximum positions of these bands is ca. 100 $cm^{-1}$ in the case of water bound in symmetric complexes, with both protons of water participating in H-bonds. In the IR spectrum of liquid water the $\nu_3$ and $\nu_1$ bands appear to merge, producing a broad band with a maximum at ca. 3300 $cm^{-1}$. Two distinct bands corresponding to the $\nu_3$ and $\nu_1$ modes of water dissolved in [emim][FAP] are seen at 3696 and 3618 $cm^{-1}$. The position of these two bands of water dissolved in [emim][FAP] indicates that the water molecules are not associated into clusters or pools of water, and can be assigned as "free" water molecules interacting via H-bonds with the [FAP] anion. This assignment is consistent with the literature data on the spectroscopic manifestation of the formation of symmetrical complexes of water molecules with weak bases.

## 7. AFM Tip and surface functionalization

200 nm Gold-coated glass slides (1 cm × 1 cm) were purchased from Ssens. To perform force spectroscopy experiments, the cantilevers were modified with the barbituric acid derivative (compound **22**) and the gold surfaces were functionalized with the Hamilton Receptor (compound **15**).

### 7.a. Barb functionalization of AFM gold-coated Si cantilevers

AFM gold-coated Si cantilevers were modified by dipping them overnight in a solution of compound **18** (1 mM total concentration in anhydrous THF) at room temperature. 15% $Et_3N$ was used to in situ deprotect the thioacetate groups. After overnight immobilization, the cantilevers were rinsed with THF. To estimate the binding of barbituric acid derivative to the gold-coated AFM tips, gold-coated glass slides acting as test samples were placed into the same immobilization solution followed by THF rinsing under the same conditions.

### 7.b. HR functionalization of gold-coated surface

SAM-coated gold surfaces were prepared in two steps:

Step i) Functionalization with 11-azidoundecane-1-thiol (compound **22**): By dipping a preliminary cleaned gold surface in a solution of $N_3C_{11}H_{23}SH$/ $C_{11}H_{23}SH$ (0.1 molar ratio) in absolute ethanol at room temperature. The incubation was done overnight and the total thiol concentration was 1 mM. After overnight SAM formation, the gold surfaces were rinsed with ethanol, dried under nitrogen.

Step ii) Click reaction: azide functionalized gold surfaces were immersed at room temperature in a water/ DMSO (1:2) solution containing 0.5 mM alkyne-Hamilton Receptor (compound **15**), 0.5 mM $CuSO_4$, 0.5 mM TBTA (tris[(1-benzyl-1*H*-1,2,3-triazol-4-yl)methyl]amine, which is a commonly used ligand for stabilizing the Cu(I) catalyst, and

2.8 mM sodium ascorbate (needed for the Cu(I) catalyst generation) for 4 h. Exposure to light was kept to a minimum. After reaction, the monolayers were rinsed with water and DMSO to ensure all physisorbed molecules to be washed off.

## 8. SAM characterization

### 8.a. Contact angle measurements.

The contact angle of water was measured using a drop shape analysis system DSA100S (Krüss). Contact angle measurements were performed directly on the gold surfaces with a 2 µL drop of ultrapure water. The contact angle value (θ) was calculated from the average of five measurements, with drops being positioned on different places on the surface.

### 8.b. ATR experiments

ATR spectra were recorded on a Bruker's ALPHA-P FTIR spectrometer at a resolution of 4 $cm^{-1}$, by coadding 254 scans. ATR experiments were performed using a single-reflection.

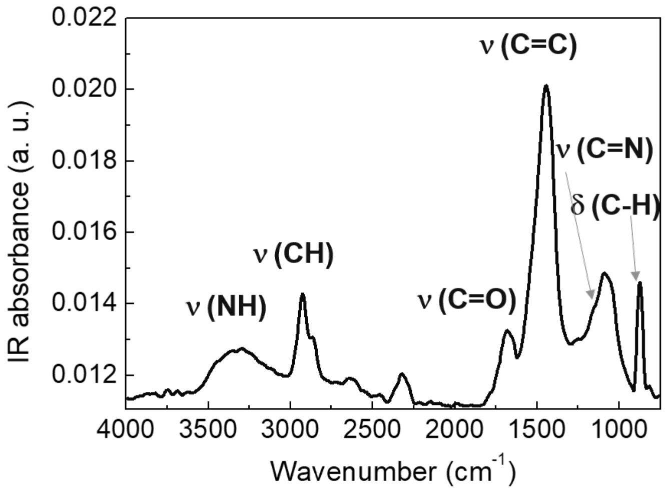


**Figure S50**. ATR-IR spectra of HR-SAM.

## 9. AFM measurements.

AFM measurements were acquired with a Nanoscope Multimode IIIa, (Veeco Instruments, Santa Barbara, CA) using gold-coated Si cantilevers Cont-GB (Budget Sensors) having nominal force constant of 0.2 N/ m and resonant frequency of 13 kHz.

Force spectroscopy measurements were performed in 1,2-dichlorobenzene and in room temperature ionic liquid as indicated at room temperature.

Force-distance measurements were recorded at velocities of 250 nm/s with contact time of 5s. After reaching the maximal force, the two surfaces remained in contact for a time interval of 5 s, after which the direction of the piezo movement was reversed, and the AFM probe was retracted from the surface. Force distance curves were collected from several different positions on the substrate, and about 60 force curves were measured at each position. The spring constants of all AFM cantilevers were defined using the Sader method.[14] Values in the range from 344 to 385 pN/nm were obtained. The same cantilever was used for each set of data, where the interactions between bare or modified AFM tip and bare or functionalized substrates were studied.

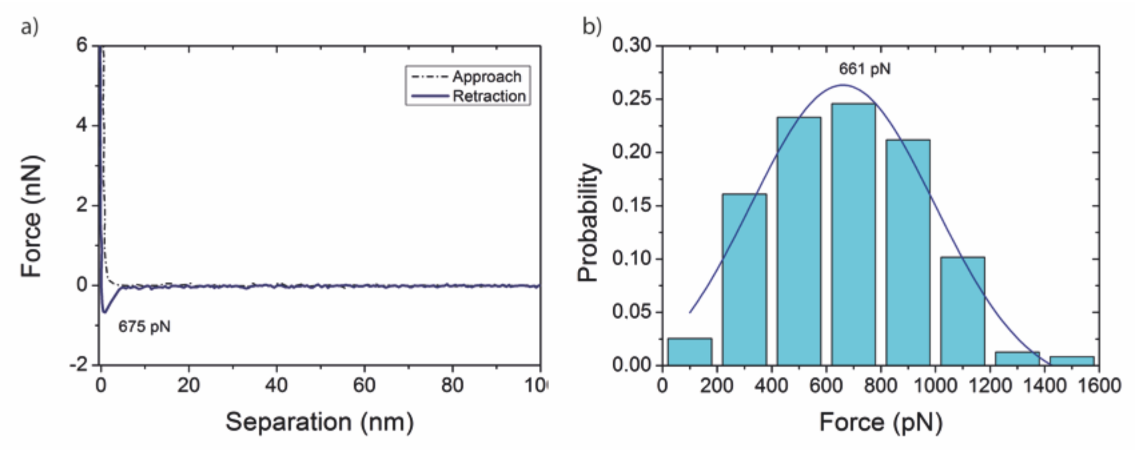


**Figure S51.** a) Representative example of a force/distance curve obtained in [emim][FAP] for the thiol-functionalized tip and HR- functionalized gold surface. b) Force histogram fitting to a Gaussian distribution, obtained for 250 curves.

**Estimation of the dissipation energy in three example force-distance curves:**

The dissipation energy is calculated in the case of force-distance curves by integrating the area in between the approach and retraction curves as shown in graph below (in pink):

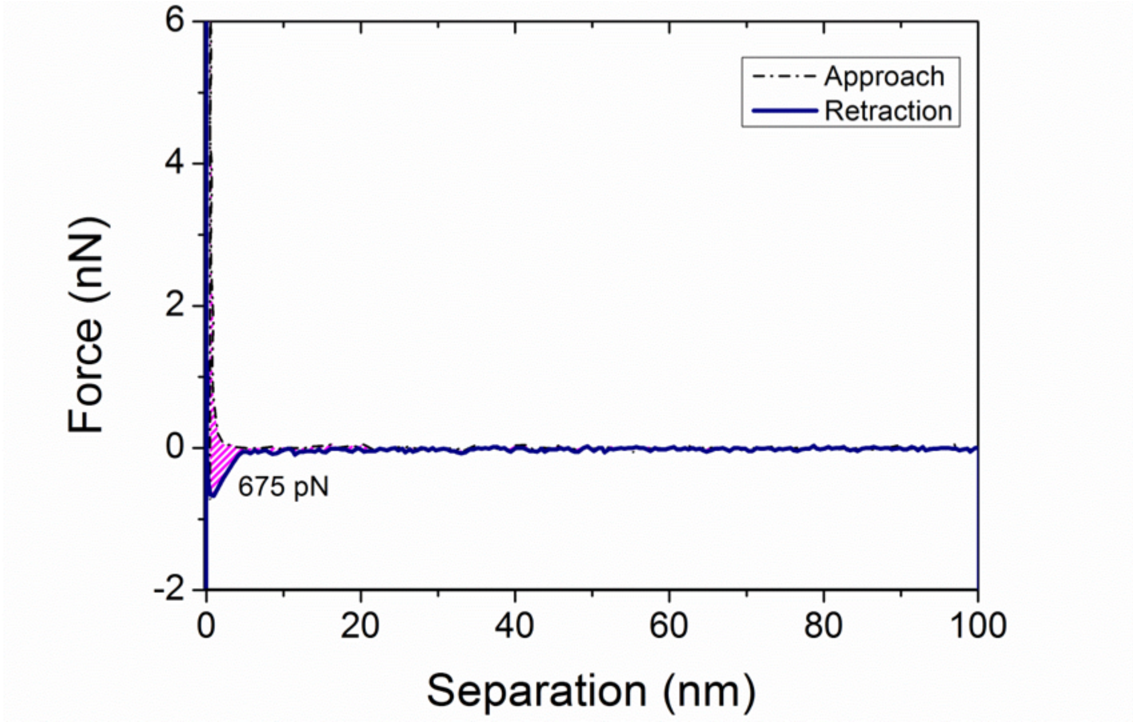


The calculation has been done by using Origin integration tools. However, we decided to take the binding force as a comparative value between the different scenarios as done by Bassani et al. because the calculus of the dissipation energy could lead to more associated errors. In particular, small changes in the limits of the integrated area produce big changes in the final calculation. In the following, the dissipation energy is calculated in the case of three typical force-distance curves at the different studied scenarios:

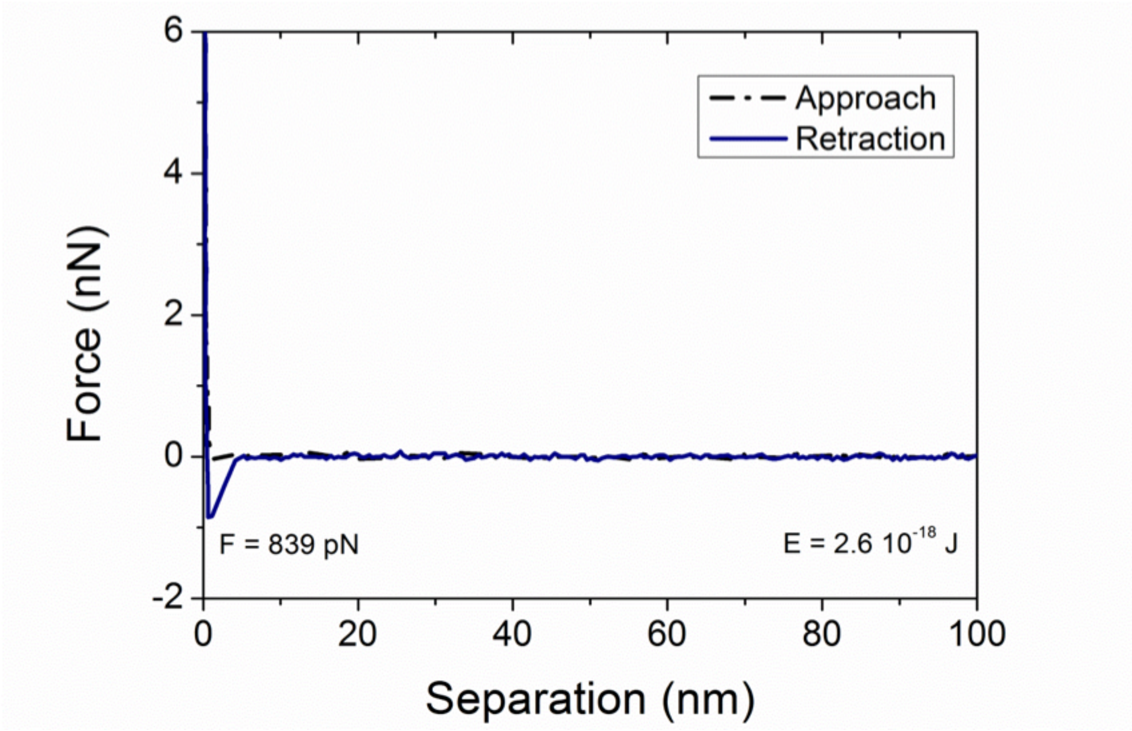


**Figure S52**. Representative force-distance curve in [emim][FAP] for the HR-cy couple (corresponding to one force-distance curve in Figure 4 in the main text).

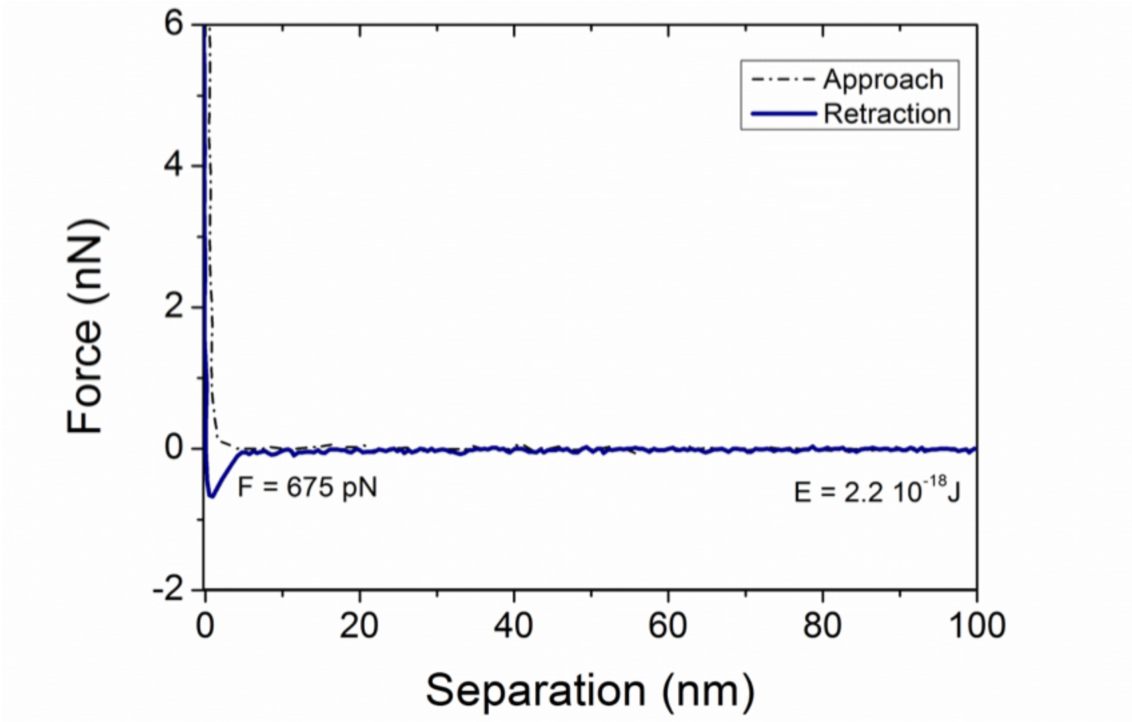


**Figure S53.** Representative force-distance curve in [emim][FAP] for the thiol-functionalized tip and HR-functionalized gold surface (corresponding to force-distance curve in Figure S46).

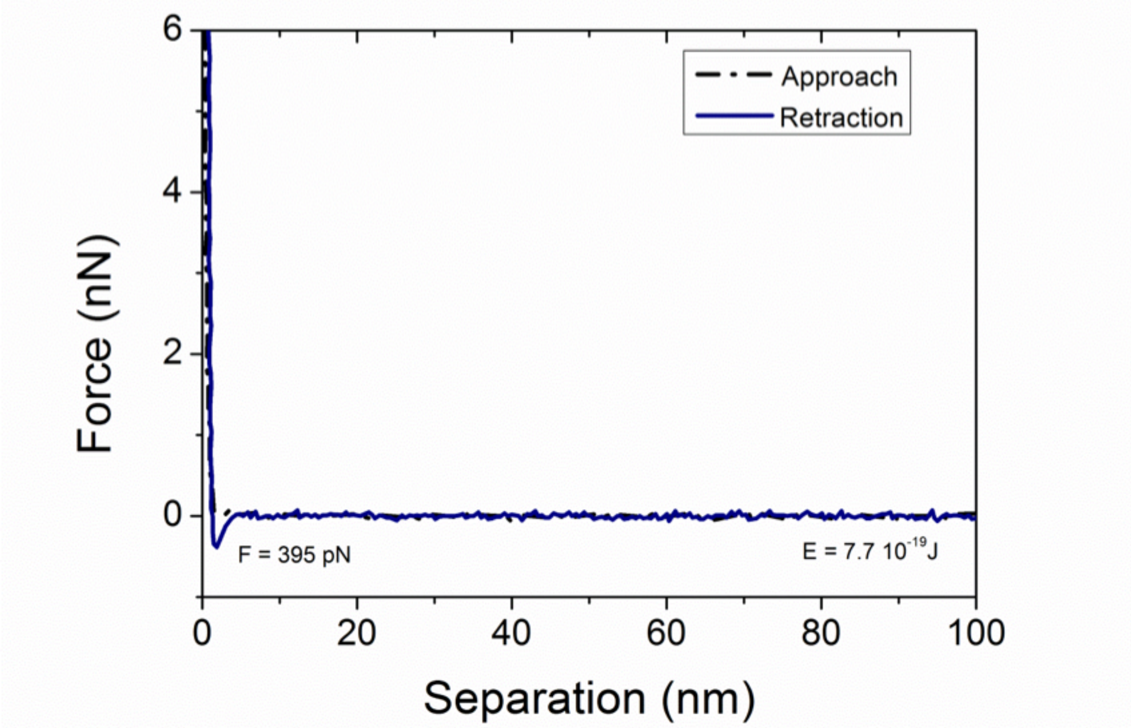


**Figure S54.** Representative force-distance curve in ODCB for the HR-cy system.

References:

1. R. Reifenberger, in Fundam. At. Force Microsc. Part I Foudnations (Eds.: M. Lundstrom, S. Datta), World Scientific Publishing Co. Pte. Ltd., 2016, pp. 49–93.
2. P. Porion, Y. R. Dougassa, C. Tessier, L. El Ouatani, J. Jacquemin and M. Anouti, *Electrochim. Acta*, 2013, **114**, 95-104.
3. V. Aravindan, J. Gnanaraj, S. Madhavi and H.-K. Liu, *Chem. Eur. J.*, 2011, **17**, 14326-14346.
4. M. C. Simoes, K. J. Hughes, D. B. Ingham, L. Ma and M. Pourkashanian, *Inorg. Chem.*, 2017, **56**, 7566-7573.
5. A. Dirksen, U. Hahn, F. Schwanke, M. Nieger, J. N. H. Reek, F. Vögtle and L. De Cola, *Chem. Eur. J.*, 2004, **10**, 2036-2047.
6. R. Gatri, I. Ouerfelli, M. L. Efrit, F. Serein-Spirau, J.-P. Lère-Porte, P. Valvin, T. Roisnel, S. Bivaud, H. Akdas-Kilig and J.-L. Fillaut, *Organometallics*, 2014, **33**, 665-676.
7. C.-H. Huang, N. D. McClenaghan, A. Kuhn, G. Bravic and D. M. Bassani, *Tetrahedron*, 2006, **62**, 2050-2059.
8. X. Liu, Y. Wang, C. Chen, A. Tintaru, Y. Cao, J. Liu, F. Ziarelli, J. Tang, H. Guo, R. Rosas, S. Giorgio, L. Charles, P. Rocchi and L. Peng, *Adv. Funct. Mater.*, 2016, **26**, 8594-8603.
9. C. Emmeluth, M. A. Suhm and D. Luckhaus, *The Journal of Chemical Physics*, 2003, **118**, 2242-2255.
10. S. Fischer, *The Journal of Chemical Physics*, 1971, **55**, 5844-5844.
11. Y. J. Hu, H. B. Fu and E. R. Bernstein, *The Journal of Chemical Physics*, 2006, **125**, 184308.
12. M. P. Conrad and H. L. Strauss, *J. Phys. Chem.* , 1987, **91**, 1668-1673.
13. P. Backx and S. Goldman, *J. Phys. Chem.*, 1981, **85**, 2975-2979.

14. J. E. Sader, J. W. M. Chon and P. Mulvaney, *Rev. Sci. Instrum.*, 1999, **70**, 3967-3969.
